# Development and clinical application of a rapid qPCR instrument featuring three independent temperature modules and a time-based algorithm for respiratory pathogen diagnosis

**DOI:** 10.1186/s12985-025-03003-2

**Published:** 2025-11-21

**Authors:** Yu Wang, Hui Wang, Meizhi Zhou, Mingzhen Wang, Tianyuan Wu, Siyuan Zhou, Xiaohong Liao, Wenkuan Liu, Xiwei Zhuang, Zhichao Zhou, Rong Zhou

**Affiliations:** 1 Guangzhou National Laboratory, Guangzhou, 510005 China; 2 Xiamen United Institutes of Respiratory Health, Xiamen, 361102 China; 3 State Key Laboratory of Respiratory Disease, National Clinical Research Center for Respiratory Disease, Guangzhou Institute of Respiratory Health, the First Affiliated Hospital of Guangzhou Medical University, Guangzhou Medical University, Guangzhou, 510210 China; 4 Foshan Fosun Chancheng Hospital, Foshan, 528031 China; 5 College of Environmental Management and Big Data, Guangdong Polytechnic of Environmental Protection Engineering, Foshan, 528216 China; 6 Wuhan Canvest Biotechnology Co., Ltd, Wuhan, 430074 China

**Keywords:** Respiratory pathogens, Independent temperature modules, Time-based algorithm, On-site, Rapid detection

## Abstract

**Background:**

Acute respiratory infections (ARIs) caused by viruses, bacteria, *Mycoplasma*, and other microorganisms rank among the most serious human health threats, and they typically manifest similar flu-like symptoms during the early stages of infection. The rapid and accurate diagnosis of pathogens causing ARIs is crucial for treating diseases and controlling pathogen transmission. Quantitative real-time polymerase chain reaction (qRT-PCR) serves as the primary detection method, yet current workflows are time-consuming and have limited multiplexing capacity; thus, they fail to meet on-site diagnostic requirements. Our aim is to develop a novel method that enhances heating and cooling rates, thereby significantly shortening PCR cycling time and enabling the on-site rapid detection of ARI pathogens.

**Results:**

A high-efficiency temperature cycling technology controlled by a time-based algorithm was developed. Its core mechanism involves the precise cyclic movement of reaction tubes among three independent temperature modules, utilizing the large temperature differences between the modules to significantly enhance heating and cooling rates. Employing this innovation, a portable fluorescence quantitative PCR (qPCR) instrument FQ-8B was developed that completed amplification in as little as 15 min. While substantially reducing amplification time, the FQ-8B maintains performance comparable to conventional instruments, demonstrating 95–105% amplification efficiency across six-channel fluorescence. The instrument exhibits exceptional specificity and reproducibility, achieving detection sensitivities of 75–100 copies/mL across diverse viruses. Clinical validation of 208 suspected severe acute respiratory syndrome coronavirus 2 (SARS-CoV-2) and 216 suspected influenza A virus (IAV) specimens showed overall concordance rates of 99.04% (kappa = 0.852, *P* < 0.001) and 95.37% (kappa = 0.881, *P* < 0.001), respectively, compared to standard instrument detection. The on-site rapid detection capacity of FQ-8B was validated using 1227 respiratory specimens from a primary hospital, demonstrating 15-pathogen screening capability per specimen within 30 min. Testing results revealed a local epidemic of four pathogens: SARS-CoV-2, influenza B virus, *Mycoplasma pneumoniae*, and IAV, from August 2023 to January 2024.

**Conclusions:**

The FQ-8B, developed based on three independent temperature modules and a time-based algorithm, demonstrates rapid, sensitive, specific, cost-effective, and portable characteristics. It provides timely on-site screening of multiple respiratory pathogens, rendering it a potent tool for infectious disease diagnosis and monitoring.

**Graphical Abstract:**

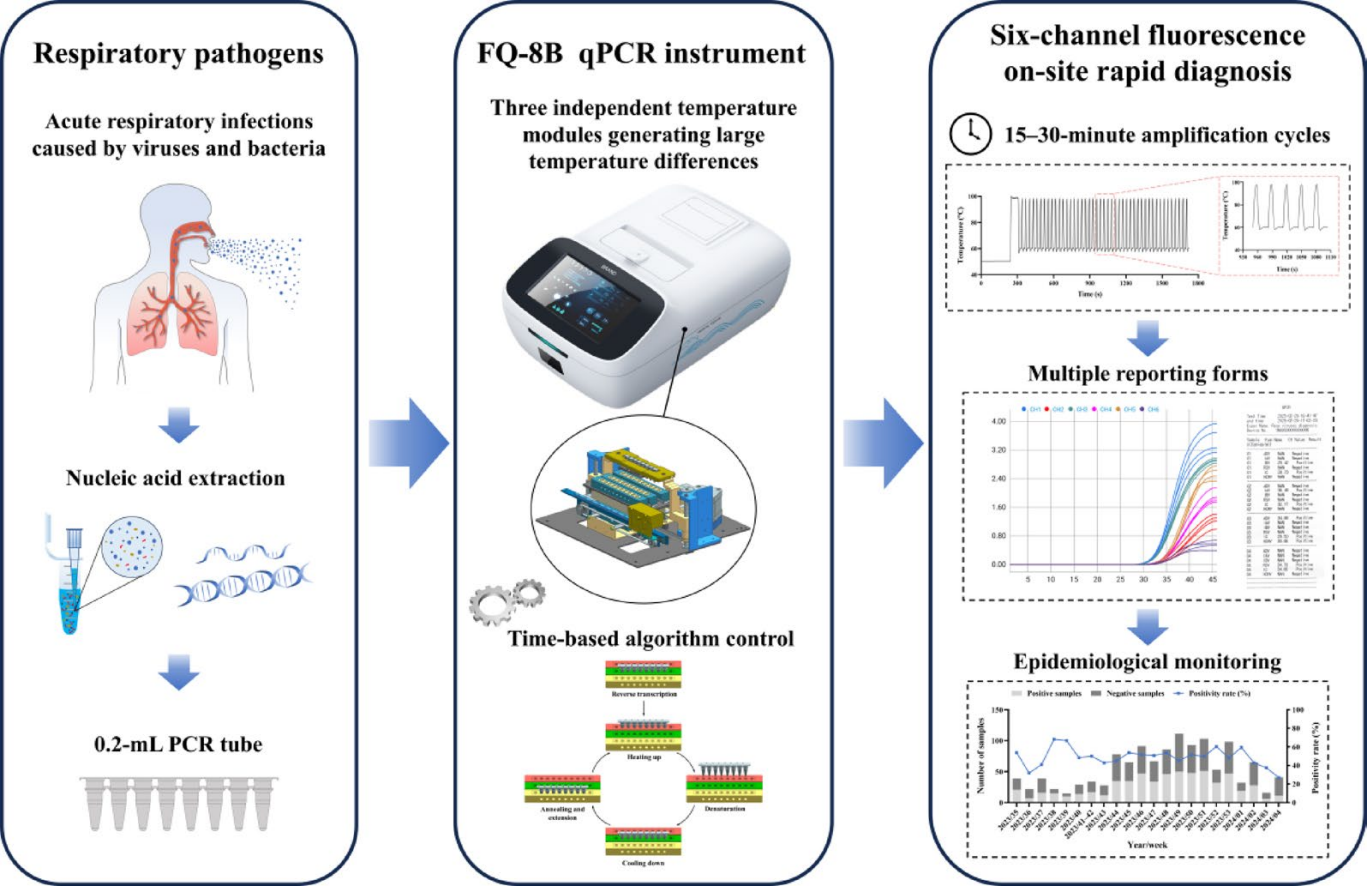

**Supplementary Information:**

The online version contains supplementary material available at 10.1186/s12985-025-03003-2.

## Background

Acute respiratory infections (ARIs) occur frequently with seasonal patterns and are a significant cause of death among children, the elderly, and immunocompromised individuals [1, 2]. The pathogens responsible for these infections are diverse, including viruses, bacteria, *Mycoplasma*, *Chlamydia*, and fungi, with approximately 80% caused by viruses [3, 4]. The Spanish flu pandemic of 1918, the novel H1N1 influenza A virus (IAV) in 2009, and severe acute respiratory syndrome coronavirus 2 (SARS-CoV-2) in 2019 all led to global public health emergencies, posing serious threats to human health and public safety [5–7]. Although treatments are available for pathogens such as IAV, SARS-CoV-2, and *Mycoplasma pneumoniae* (MP), infections caused by viruses and bacteria often exhibit similar symptoms yet tend to require specific drug treatments. Due to the limited timeliness and accuracy of conventional diagnostics, empirical broad-spectrum antibiotic therapy is often administered, raising concerns of drug-resistant bacteria [8, 9]. Furthermore, studies have shown that severe respiratory infection and even death are significantly correlated with the type of infectious pathogen and the timing of therapeutic intervention [10–12]. These factors collectively underscore the critical importance of early, rapid, and accurate pathogen diagnosis.

Currently, diagnostic technologies for respiratory pathogens can be categorized into pathogen isolation and culture, antigen-antibody assays, and molecular diagnostics using nucleic acid amplification tests (NAATs) [13, 14]. Pathogen isolation and culture are generally considered the gold standard for identification and play a crucial role in in-depth pathogen research. However, due to its low sensitivity, long culture times, technical challenges, and biosafety risks, viral culture is now rarely used for diagnostic purposes in clinical laboratories [15]. Antigen-antibody detection relies on the identification of pathogen antigens by an appropriate antibody; techniques such as enzyme-linked immunosorbent assay, chemiluminescence immunoassay, and lateral flow immunoassay have been developed based on this principle [16–19]. These methods are convenient with low technical requirements, but they can also face significant development difficulties, lengthy cycles, suboptimal analytic performance, and delayed detection [20, 21]. NAATs include quantitative real-time polymerase chain reaction (qRT-PCR), isothermal amplification technology, clustered regularly interspaced short palindromic repeat (CRISPR)-associated detection, and next-generation sequencing [22–24]. While each of these has pros and cons, qRT-PCR remains the predominant NAAT methodology due to its superior detection sensitivity, target pathogen flexibility, pathogen quantification capacity, and rapid turnaround time [25]. qRT-PCR technology is also mature and is cost-effective both during development and in actual use. qRT-PCR can be used to quickly establish a detection method for emerging infectious diseases and is widely studied and applied in clinical practice [26].

However, rapidly identifying respiratory pathogens via qRT-PCR still faces numerous challenges. The primary challenge lies in the wide variety of pathogens and the common occurrence of co-infection, while the limited number of fluorescent channels in existing detection instruments, coupled with potential interference between fluorescent signals, often limits detection to no more than four targets in a single reaction [27, 28]. Additionally, traditional fluorescence quantitative PCR (qPCR) instruments rely on a single temperature module for cycling, with a rate not exceeding 4.5 °C/s [29]. Even DNA fragments with an amplification length of no more than 200 base pairs (bp) require more than an hour of cycling and therefore cannot achieve rapid amplification [30]. Furthermore, most current systems are high-throughput, bulky, and require costly instruments and trained technicians in controlled laboratory settings [31]. Yet, field applications such as fever clinics, rural areas, customs quarantine stations, and airports require immediate on-site pathogen analysis, and therefore, there is a critical need for portable, user-friendly devices [32]. Consequently, developing an efficient, cost-effective, and portable qRT-PCR system for rapid, multiplexed detection of respiratory pathogens has become critically imperative.

In this study, we developed a temperature control technology using a time-based algorithm. By cycling reaction tubes among three independent temperature modules (ITMs) and taking advantage of the large temperature differences between the modules, the heating and cooling rates were significantly increased. Using this technology, we developed a rapid qPCR instrument, FQ-8B, which was equipped with six fluorescence channels and was compatible with conventional 0.2-mL PCR tubes. This instrument dramatically reduces reaction time, has broad applicability, and has demonstrated excellent performance in qRT-PCR experiments, providing precise and reliable analysis support for scientific research and clinical applications.

## Materials and methods

### Cells, viruses, and clinical specimens

AD293 cells (from American Type Culture Collection) were stored in our laboratory. Respiratory syncytial virus (RSV; RSV-A2 strain), adenovirus (ADV; ADV3, ADV4, ADV7, and ADV55 strains), IAV (H1N1 strain), influenza B virus (IBV; Victoria strain), herpes simplex virus type 1 (HSV-1), human parainfluenza virus (HPIV; HPIV-1 strain), and MP were stored and provided by our laboratory. Human coronavirus OC43 (HCoV-OC43), human coronavirus 229E (HCoV-229E), human coronavirus NL63 (HCoV-NL63), human rhinovirus (HRV; HRV-A strain), human bocavirus (HBoV; HBoV-1 strain), and human metapneumovirus (HMPV; HMPV-A2 strain) were obtained from the State Key Laboratory of Respiratory Disease at Guangzhou Medical University. A pseudovirus of SARS-CoV-2 was purchased from Sansure Biotech Inc (Changsha, China). Inactivated frozen clinical specimens from 208 suspected SARS-CoV-2-infected patients were provided by the First Affiliated Hospital of Guangzhou Medical University. Inactivated frozen clinical specimens from 216 suspected IAV-infected patients and 1227 suspected cold and fever patients were provided by the Hongshan Street Community Health Service Center in Huangpu District, Guangzhou. All experiments were performed in accordance with biosafety regulations.

### Nucleic acid extraction

A DNA and RNA extraction reagent (magnetic bead method) was purchased from HuYanSuo Medical Technology Co., Ltd. in Guangzhou, China. Briefly, 200 µL of each sample was added to a 96-well plate preloaded with the extraction reagent, and the nucleic acid was isolated from each sample using an E-Five nucleic acid extraction instrument (HuYanSuo Medical Technology Co., Ltd., Guangzhou, China), according to the manufacturer’s instructions. After extraction, 100 µL of elution buffer containing the nucleic acid was stored at − 80 °C for further use.

### Modules and features of the FQ-8B

**Fig. 1:**
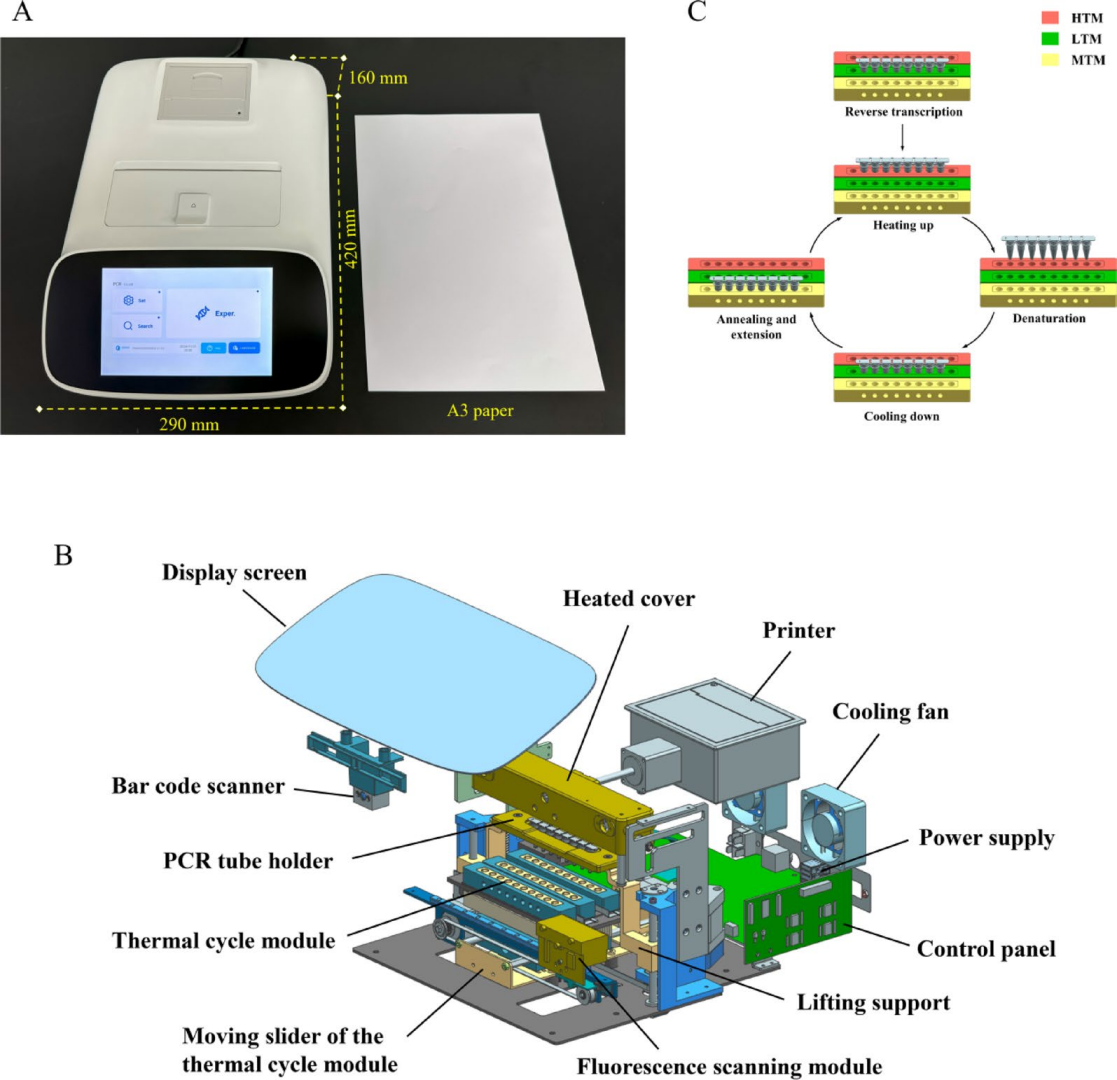
The FQ-8B and its thermal cycling. (**A**) Dimensions of the FQ-8B qPCR instrument. (**B**) Schematic diagram of the functional modules of the instrument. (**C**) Schematic of the thermal cycling amplification using the three independent temperature modules. HTM, high-temperature module; LTM, low-temperature module; MTM, medium-temperature module

The FQ-8B dimensions are 420 mm × 290 mm × 160 mm, occupying approximately the area of A3 paper, and the instrument weighs 6.6 kg (Fig. 1A). It consists of five major modules: a human-computer interaction module, a thermal cycling module, a rack module, a fluorescence detection module, and an electronic system module (Fig. 1B). The human-computer interaction module integrates an 8-inch display screen, a bar code scanner, and a printer for system operation, sample identification, and automated report generation, respectively. The thermal cycle module comprises three thermostatic zones: a high-temperature module (HTM), a low-temperature module (LTM), and a medium-temperature module (MTM) (BYD Company, Ltd., Shenzhen, China). The MTM and LTM have integrated bottom-mounted Peltier elements enabling rapid thermal transitions. Each zone has 10 wells, with the middle eight wells accommodating 0.2-mL PCR eight-tube strips that hold 10–50 µL of a liquid reaction mixture, with the two side wells housing a thermometer probe. The MTM additionally features side-positioned micro-orifices for real-time fluorescence monitoring of the reaction. The rack module incorporates a PCR tube holder and a heated cover. The heated cover maintains a high temperature of 105 °C and fits tightly against the eight-tube lids through a slide. The PCR tube holder moves up and down, allowing the eight-tube strips to move between the three ITMs. The fluorescence detection module comprises a fluorescence scanning module (Hema Medical Instrument Co., Ltd., Zhuhai, China) and a sliding track. Light-emitting diode light sources of different wavelengths are irradiated onto the PCR tubes through lenses and filters, exciting the fluorescence of the amplified product. Subsequently, the fluorescence signals emitted by the amplified product are received by a photomultiplier tube, and the photoelectric signals are converted by a microcontroller to collect and store the fluorescence values after each scan. The fluorescence detection module can simultaneously detect up to six different channels: Atto 425, FAM, VIC, ROX, Cy5, and Quasar 705. The electronic system module includes a power supply and a control panel to deliver operational power while coordinating component functionality through centralized regulation.

### Thermal cycling process of the FQ-8B

The FQ-8B achieves rapid temperature changes of the PCR reaction by transferring PCR tubes between the HTM and LTM and exploiting the resulting large temperature differences. Each of the three temperature modules has a customizable preset temperature, and the PCR tubes enter the corresponding temperature module according to the temperature instructions in the procedure. For a two-step qRT-PCR, the PCR tubes first enter the LTM for reverse transcription, and the temperature of the LTM rises to the set reverse transcription temperature. When reverse transcription completes, the PCR tubes enter the HTM for heating; the temperature of the HTM is usually set to above 100 °C, and it remains unchanged throughout the process. During this time, the temperature of the LTM rapidly decreases to its preset temperature. When the programmed denaturation temperature is reached, the PCR tube holder lifts the tubes, pauses momentarily, and returns the tubes to the HTM. The movements maintain the PCR internal temperature near the set denaturation point, preventing the PCR contents from overshooting to the HTM temperature. After denaturation, the PCR tubes are transferred to the LTM for cooling. When the programmed annealing and extension temperatures are reached, the holder transfers the tube to the MTM, which has a temperature matching the set annealing and extension temperature in the procedure. Once the extension step is complete, the fluorescence scanning module collects fluorescence signals through a small hole on the side of the MTM. Subsequently, the PCR tubes re-enter the HTM to commence the next round of cycling (Fig. 1C).

To control the amount of time over which the temperature would rise and fall during the PCR reaction, we used two methods: a thermometer probe and a time-based algorithm. Probe monitoring determines when heating and cooling end based on the PCR tube temperature measured by the two thermometer probes located on the outermost side of the temperature module. The time-based algorithm is a fitted formula derived from multiple temperature rise and fall tests conducted on different liquid volumes using a specially designed liquid temperature measurement tool (BYD Company, Ltd., Shenzhen, China) and a data collector (Keysight Technologies Inc., CA, USA). This formula is used to calculate the time required for the temperature to rise and fall in the PCR tube reaction mixture.

### Quantitative real-time PCR

A qRT-PCR experiment was conducted using the FQ-8B, an Applied Biosystems QuantStudio 7 (ABI-Q7) Flex real-time PCR system (Thermo Fisher Scientific, MA, USA), and a SLAN-96P real-time PCR system (Hongshi Medical, Shanghai, China). The TaqMan probe-based qRT-PCR kits used in this study are listed in Additional File 1: Table S1. The reaction systems were prepared and procedures for each analysis kit were performed according to the manufacturer’s instructions. Levels of the housekeeping genes glyceraldehyde-3-phosphate dehydrogenase (*gapdh*), *ADV3*, *ADV4*, *ADV7*, and *ADV55* were determined using SYBR Green dye (Tsingke Biotech Co., Ltd., Beijing, China). A 20-µL reaction system was used, which consisted of 10 µL of ArtiCan^ATM^ SYBR qPCR mix, 1.5 µL of each forward and reverse primer (Additional File 1: Table S2), and 7 µL of DNA template. The PCR cycling procedure was as follows: 95 °C for 1 min; 40 cycles of 95 °C for 10 s and 60 °C for 20 s; and a melting curve analysis.

### Amplification effect of the FQ-8B

Using a plasmid containing the full-length gene sequence of enterovirus (EV) 71 as a template, a conventional PCR instrument (LongGene Scientific Instruments Co., Ltd., Hangzhou, China) using both standard and rapid procedures and FQ-8B with its rapid procedure were used with Taq polymerase (Takara Biomedical Technology Co., Ltd., Beijing, China) to amplify DNA fragments with lengths of 100, 200, 500, 1000, 2000, and 5064 bp. The time required to amplify each fragment was recorded, and the DNA fragments amplified by each instrument were analyzed using 1.5% agarose gel electrophoresis.

Next, after extracting the nucleic acid from AD293 cells, four pairs of different primers were used to amplify the *gapdh* gene using SYBR Green dye on the FQ-8B, ABI-Q7, and SLAN-96P instruments. The amplification curves, raw melting curves, derivative melting curves, and corresponding cycle threshold (Ct) values and melting temperature (*T*_*m*_) values obtained from the different instruments were analyzed to evaluate the real-time fluorescence detection and melting curve functions of the FQ-8B.

To evaluate the amplification efficiency (EFF) of the FQ-8B six-channel fluorescence, positive reference samples corresponding to each channel were diluted with diethyl pyrocarbonate (DEPC) water in a 10-fold gradient. During testing, six gradient templates for each channel were subjected to qRT-PCR experiments on the FQ-8B in each round. Standard curves were plotted based on the Ct values obtained from three replicates, and the *R*^*2*^ of each curve and the EFF of each channel were calculated [33].

### Specificity, reproducibility, and sensitivity of the FQ-8B

The specificity of the FQ-8B was evaluated using nucleic acids from 12 prevalent respiratory pathogens (RSV, ADV, IAV, HSV-1, HCoV-229E, HCoV-OC43, HCoV-NL63, HRV, HBoV, HMPV, HPIV-1, and MP) and the corresponding reagents for each pathogen. Each reagent was cross-tested against 12 pathogen nucleic acids, with each nucleic acid tested three times, and the results obtained on the ABI-Q7 served as a control. We also evaluated the virus typing ability of the FQ-8B by distinguishing low-concentration ADV3 from medium- and high-concentration ADV4, ADV7, and ADV55, all of which exhibit a level of homology [34].

The reproducibility of the FQ-8B was evaluated using positive reference samples from a 2019-nCoV nucleic acid diagnostic kit (Sansure Biotech Inc., Changsha, China) at high, medium, and low concentrations. The testing was performed over 3 days, during which two operators conducted repeated tests on three concentrations of samples, each with eight replicates, using one instrument per day in different laboratories. The standard deviation and coefficient of variation (CV) were calculated for all Ct values from each device across the test days for each concentration to evaluate the reproducibility. The average Ct values between the two instruments at each concentration were compared, and statistical analysis was performed to assess any discrepancies.

The nucleic acids of RSV, ADV, IAV, IBV, HCoV-OC43, HCoV-229E, and HBoV were then quantified using digital PCR (Aperbio Technologies Co., Ltd., Suzhou, China). The quantified nucleic acids and SARS-CoV-2 pseudovirus standard nucleic acid (Sansure Biotech Inc., Changsha, China) were diluted with DEPC water to concentrations of 1000, 100, 75, 50, 25, 10, and 0 copies/mL (negative control). The first seven viruses underwent single-gene analysis. Positive results were confirmed by observing the amplification curves and Ct values of the viral genes. For the SARS-CoV-2 standard, dual-gene detection was used, and positive results were confirmed according to the amplification curves and Ct values of both viral genes. Initially, 24 tests were conducted on the FQ-8B at the different concentrations of each virus, and the concentration at which 23 or more positive results were obtained was defined as the detection limit. These results were compared with those obtained from the ABI-Q7 and SLAN-96P instruments. Subsequently, 24 more tests were performed on the FQ-8B with nucleic acid concentrations of 75, 50, and 25 copies/mL to more accurately determine the detection limit of each virus.

### Clinical performance of the FQ-8B

After extracting nucleic acids from the frozen respiratory specimens (throat swabs) collected from suspected SARS-CoV-2- and IAV-infected patients, qRT-PCR tests were conducted on the FQ-8B and ABI-Q7 using a 2019-nCoV nucleic acid diagnostic kit (Sansure Biotech Inc., Changsha, China) and a SARS-CoV-2 and influenza A/B virus nucleic acid diagnostic kit (Sansure Biotech Inc., Changsha, China), respectively. SARS-CoV-2 detection was considered positive if the Ct value of either the FAM channel or the ROX channel was ≤ 40 and the Ct value of the VIC channel was also ≤ 40. IAV detection was considered positive if the Ct values of both the VIC channel and the Cy5 channel were ≤ 40. If either or both of the qPCR instruments identified SARS-CoV-2 or IAV as positive, the specimen was recorded as positive for the corresponding virus; otherwise, it was recorded as negative.

A total of 1227 frozen respiratory specimens were collected over a 23-week period from August 2023 to January 2024 at Hongshan Street Community Health Service Center, a primary healthcare institution. Following nucleic acid re-extraction, qRT-PCR tests were performed on the FQ-8B using a multiplexed nucleic acid diagnostic kit containing pre-loaded 0.2-mL PCR eight-tube strips, with a four-tube combined test design for 15 respiratory pathogens (Huirui Biotechnology Co., Ltd., Zhuhai, China). The four tubes were run simultaneously on the qPCR instrument, with each tube tested for three to four different pathogens or an internal reference gene through the FAM, VIC, ROX, and Cy5 channels. Each specimen underwent nucleic acid analysis for a total of 15 pathogens: RSV, ADV, IAV, IBV, SARS-CoV-2, HSV-1, HMPV, EV, HRV, HBoV, HPIV (including types 1, 2, and 3), HCoV (including OC43, 229E, NL63, and HKU-1), MP, *Chlamydia pneumoniae* (CP), and *Bordetella pertussis* (BP). If the Ct value of any target among these 15 pathogens was ≤ 40 and the internal reference gene Ct value was also ≤ 40, the specimen was recorded as positive for that pathogen; otherwise, it was considered negative. If the Ct values of two or more targets among these 15 pathogens were ≤ 40 and the internal reference gene Ct value was also ≤ 40, the specimen was recorded as co-infected, with the pathogen types noted. Because EV and HRV both belong to the enterovirus genus, they share similarities in the highly conserved sequences of the 5’ non-coding region, and there have been reports of cross-reactivity between EV primers and HRV. Therefore, positive results for HRV, EV, or both were recorded as HRV/EV [35, 36].

### Statistical analysis

SPSS Statistics 20 software (IBM, Armonk, NY, USA) was used to calculate the percentage of positive and negative agreements between the FQ-8B and conventional qPCR instrument, and Cohen’s kappa statistic was calculated [37]. Statistical graphics were generated using GraphPad Prism 8 software (GraphPad Software Inc., San Diego, CA, USA), and statistical analysis was performed using Student’s *t* tests or ANOVA tests. *P* < 0.05 was considered statistically significant.

## Results

### Three independent temperature modules of the FQ-8B qPCR instrument

**Fig. 2:**
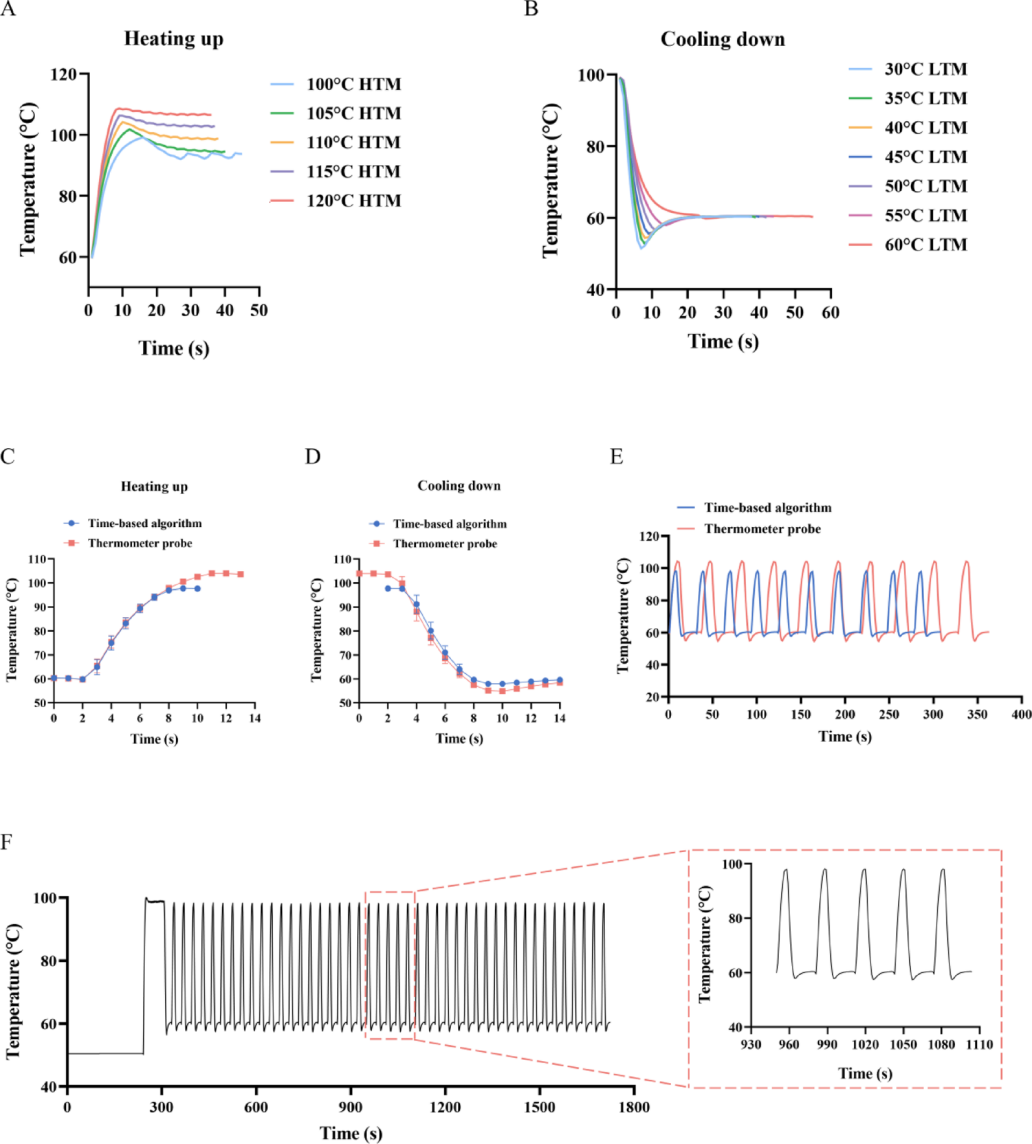
Thermometer probe and time-based algorithm temperature control. (**A**) Temperature curves of heating the reaction mixture using a thermometer probe and HTM at different temperatures. (**B**) Temperature curves of cooling the reaction mixture using a thermometer probe and LTM at different temperatures. (**C**)–(**E**) Comparison of the heating rates, cooling rates, and total cycle time over 10 cycles controlled by either a thermometer probe or time-based algorithm. (**F**) Temperature curve of a complete qRT-PCR procedure using the time-based algorithm. Reaction conditions: reverse transcription at 50 °C for 240 s, pre-denaturation at 95 °C for 30 s, followed by 45 cycles of denaturation at 95 °C for 1 s and annealing and extension at 60 °C for 15 s. HTM, high-temperature module; LTM, low-temperature module

We initially optimized the HTM temperature, considering the thermal stability of DNA polymerase and the use of common 0.2 mL-polypropylene PCR tubes, by setting the HTM range at 100–120 °C. A thermometer probe was also used to control the heating time. When the liquid in the tube was heated from 60 °C to 95 °C and held for 30 s, the higher temperature of the HTM produced a faster heating rate and therefore a shorter time was required for denaturation. Because of the probe control, temperature overshoot occurred at 95 °C, and subsequently raising and lowering the tubes gradually brought the temperature back to a constant value (Fig. 2A). In experiments in which the HTM was set to 120 °C, 115 °C, 110 °C, and 105 °C, the corresponding overshoot/constant temperatures were 108.6 °C/106.5 °C, 106.3 °C/102.6 °C, 104.2 °C/98.5 °C, and 101.8 °C/94.3 °C, respectively. With a 100 °C HTM, the liquid overshoot to 99.2 °C but dropped to 92 °C during the raising and lowering process, which was lower than the 95 °C setpoint, causing temperature fluctuations rather than reaching a stable value. We found that although the set denaturation temperature determined the degree of overshoot, the final constant temperature was solely related to the HTM temperature itself. Considering the faster heating rate and the fact that the denaturation temperature in a typical PCR ranges from 94 °C to 98 °C, we set the HTM to 110 °C.

The annealing and extension temperatures of the two-step process were usually between 55 °C and 65 °C. We then cooled the liquid from 95 °C to 60 °C and explored the optimal LTM temperature. Similarly, when the thermometer probe detected that the liquid reached 60 °C, the PCR tube was moved from the LTM to the MTM. During this process, the temperature of liquid first continued to decrease until the tube was fully in the MTM; then, it gradually rose back to 60 °C. It was observed that a lower LTM temperature produced a faster cooling rate, and therefore, a shorter time was required for the extension step (Fig. 2B). The lowest temperatures of the liquid reached by the LTM at 30, 35, 40, 45, 50, and 55 °C were 51.5, 52.7, 54.4, 55.6, 56.6, and 57.9 °C, respectively, while the LTM at 60 °C only slowly cooled the liquid to 60 °C. With an extension step set to 30 s, the LTM was operated at 30, 35, 40, 45, 50, 55, and 60 °C, during which the actual time required to maintain the liquid temperature between 59 °C and 60 °C was 24, 25, 25, 26, 27, 28, and 36 s, respectively. Taking into account the cooling rate, the duration near 60 °C, and an actual extension temperature typically between 55 °C and 65 °C, we ultimately set the LTM temperature to 40 °C.

During the thermometer probe control, overshoot occurred during both heating and cooling. To minimize the effects of the high temperature overshoot and insufficient extension time at low temperature, we implemented a time-based algorithm to control the rise and fall of the PCR tube temperature. Setting the HTM to 110 °C and the LTM to 40 °C, we fitted an algorithm through extensive testing. After 10 cycles of 95 °C denaturation for 1 s and 60 °C annealing and extension for 15 s using both methods, we observed that the time-based algorithm reduced the degree of overshoot during the heating process, with the average maximum temperature decreasing from 103.9 °C to 97.7 °C. Except for the overshoot, the curves during the heating process almost coincided, indicating a consistent temperature increase rate (Fig. 2C). During cooling, the temperature differences produced by the time-based algorithm were less than those of the temperature probe, resulting in a slightly slower rate of temperature decrease. However, the average minimum temperature increased from 54.8 °C to 57.9 °C, and the average time maintained between 59 °C and 60 °C increased from 12.5 s to 14 s (Fig. 2D). Comparing the complete temperature curves of 10 cycles, the time-based algorithm was 55 s faster than the thermometer probe (Fig. 2E). These results showed that the time-based algorithm provided more precise temperature control than the thermometer probe. Subsequently, we ran a complete 45-cycle qRT-PCR procedure using the time-based algorithm. Due to the longer heating time, the highest temperature reached 100 °C during the first temperature increase from reverse transcription to pre-denaturation but then stabilized at 98 °C. The highest and lowest temperatures for each subsequent cycle were also approximately 98 °C and 58 °C, respectively (Fig. 2F). This temperature control method therefore meets the needs of most PCR reagents and enzyme activities. More importantly, the maximum heating and cooling rates of the entire process were both 11.8 °C/s, with a total time of less than 29 min. In contrast to conventional qPCR instruments that take 1 h to run the same procedure, the efficiency was greatly improved.

### Time-based algorithm controls the amplification effect as the temperature rises and falls

**Fig. 3:**
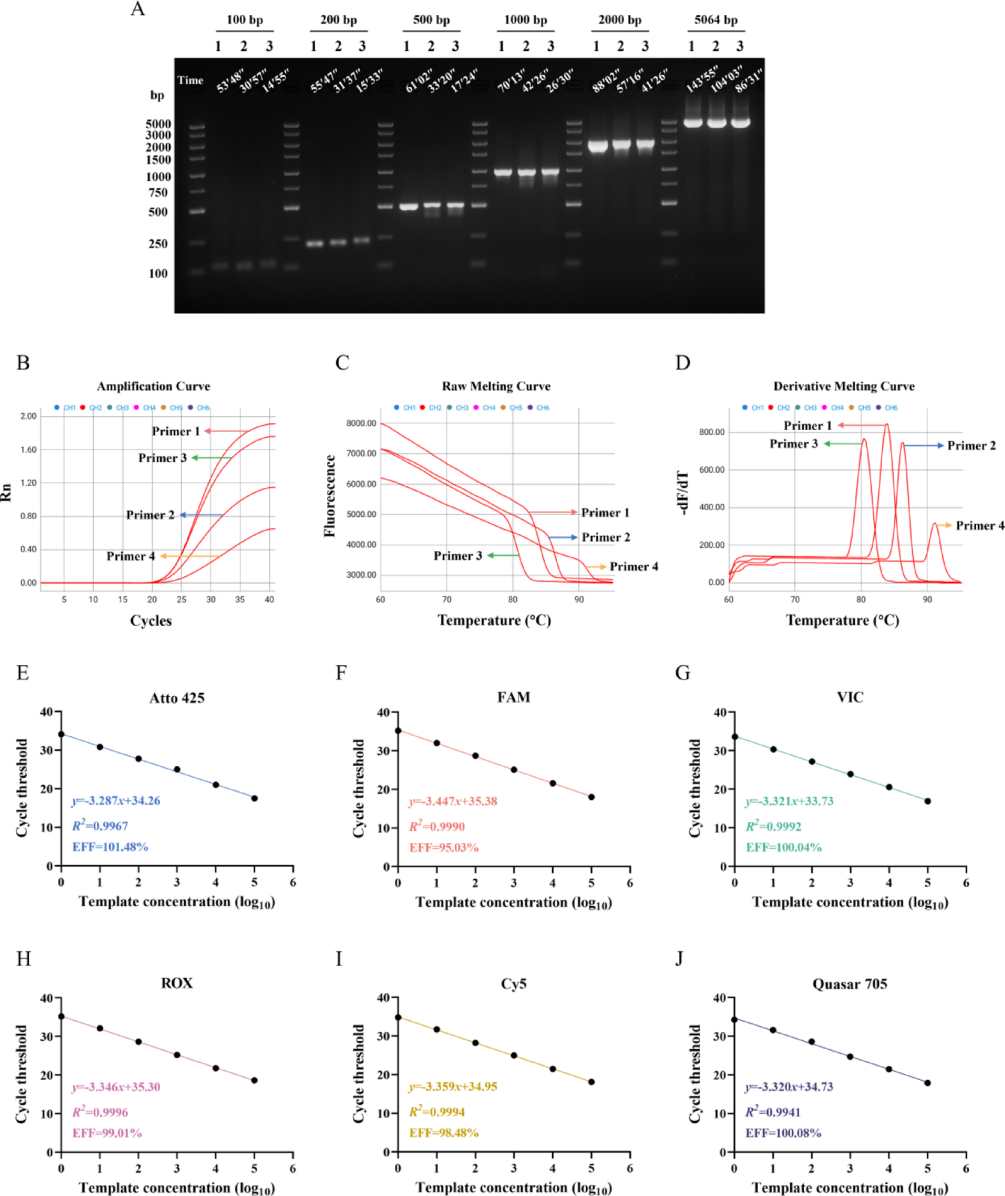
Amplification by the FQ-8B using the time-based algorithm for temperature control during heating and cooling. (**A**) Amplification of 100-, 200-, 500-, 1000-, 2000-, and 5064-bp DNA fragments by the FQ-8B and a conventional PCR instrument. Lane 1: conventional PCR instrument using the standard amplification procedure. Lane 2: conventional PCR instrument using the rapid amplification procedure. Lane 3: FQ-8B using the rapid amplification procedure. The full length of the gel is presented in Additional File 5: Raw image. (**B**)–(**D**) The amplification curves, raw melting curves, and derivative melting curves obtained using with FQ-8B to amplify the *gapdh* gene using four different primer pairs. (**E**)–(**J**) The standard curves for the six fluorescence channels (Atto 425, FAM, VIC, ROX, Cy5, and Quasar 705) obtained using the FQ-8B. *gapdh*, glyceraldehyde-3-phosphate dehydrogenase

To verify the effects of the time-based algorithm used to control the temperature rise and fall and of the three ITMs for cycle amplification, we used the FQ-8B and a conventional PCR instrument to amplify DNA fragments of varying lengths using both the standard three-temperature cycling steps and rapid two-temperature cycling steps (Additional File 1: Table S3). The amplification results of the 100-, 1000-, and 5064-bp fragments showed that the target band brightness obtained by the three methods was almost identical, but the standard procedure exhibited almost no smearing when amplifying the 1000-bp fragment, indicating better specificity. The amplification results of the 200-, 500-, and 2000-bp fragments showed that the band amplified by the standard procedure was the brightest and exhibited the best specificity, while both instruments using the rapid procedure obtained virtually the same results (Fig. 3A). This indicated that, depending on primers and amplification sequences, the standard three-step method may yield better results, but when using the two-step method, there was almost no difference between the FQ-8B and a conventional PCR instrument. Furthermore, the two-step method was significantly less time-consuming than the three-step method [38]. The faster temperature rise and fall of the FQ-8B decreased processing time by 15–20 min compared to the conventional PCR instrument when running the rapid procedure; the shorter the amplified band, the more pronounced the time advantage of the FQ-8B became, which completed the PCR in as little as 15 min.

Next, the FQ-8B qRT-PCR performance was tested. The fluorescence scanning module collected the fluorescence signals from each round of amplification, and the instrument then plotted the real-time amplification curves. When the procedure completed, the FQ-8B automatically analyzed the data and determined the Ct value using a second-order derivative algorithm. Four primer pairs amplified *gapdh* gene in one test, yielding amplification curves and Ct values of 22.87, 22.24, 22.55, and 23.38 (Fig. 3B). After melting curve analysis, the data underwent exponential background removal and normalization to generate negative derivative plots, yielding *T*_*m*_ values of 84.0, 86.4, 80.4, and 91.2 °C, respectively (Fig. 3C and D). Due to the use of different fluorescence detectors and algorithms in the different qPCR instruments, there were slight differences among the Ct values obtained with the four pairs of primers on the FQ-8B, ABI-Q7, and SLAN-96P. Notably, for each pair of primers, the differences in *T*_*m*_ values across the three instruments were within about 0.5 °C (Additional File 2: Figure S1). This confirmed that the FQ-8B delivers qRT-PCR performance on par with conventional qPCR instruments.

Subsequently, we tested the amplification performance of six-channel fluorescence: Atto 425, FAM, VIC, ROX, Cy5, and Quasar 705. The results indicated good consistency among the amplification curves of all channels over three trials (Additional File 3: Figure S2). Standard curves derived from the amplification results of each channel showed correlation coefficients (*R*^*2*^) greater than 0.99, with an EFF ranging from 95% to 105% (Fig. 3E–J). This suggested that the six-channel fluorescence exhibited excellent amplification with the temperature rise and fall controlled by the time-based algorithm.

### Specificity, reproducibility, and sensitivity

**Fig. 4:**
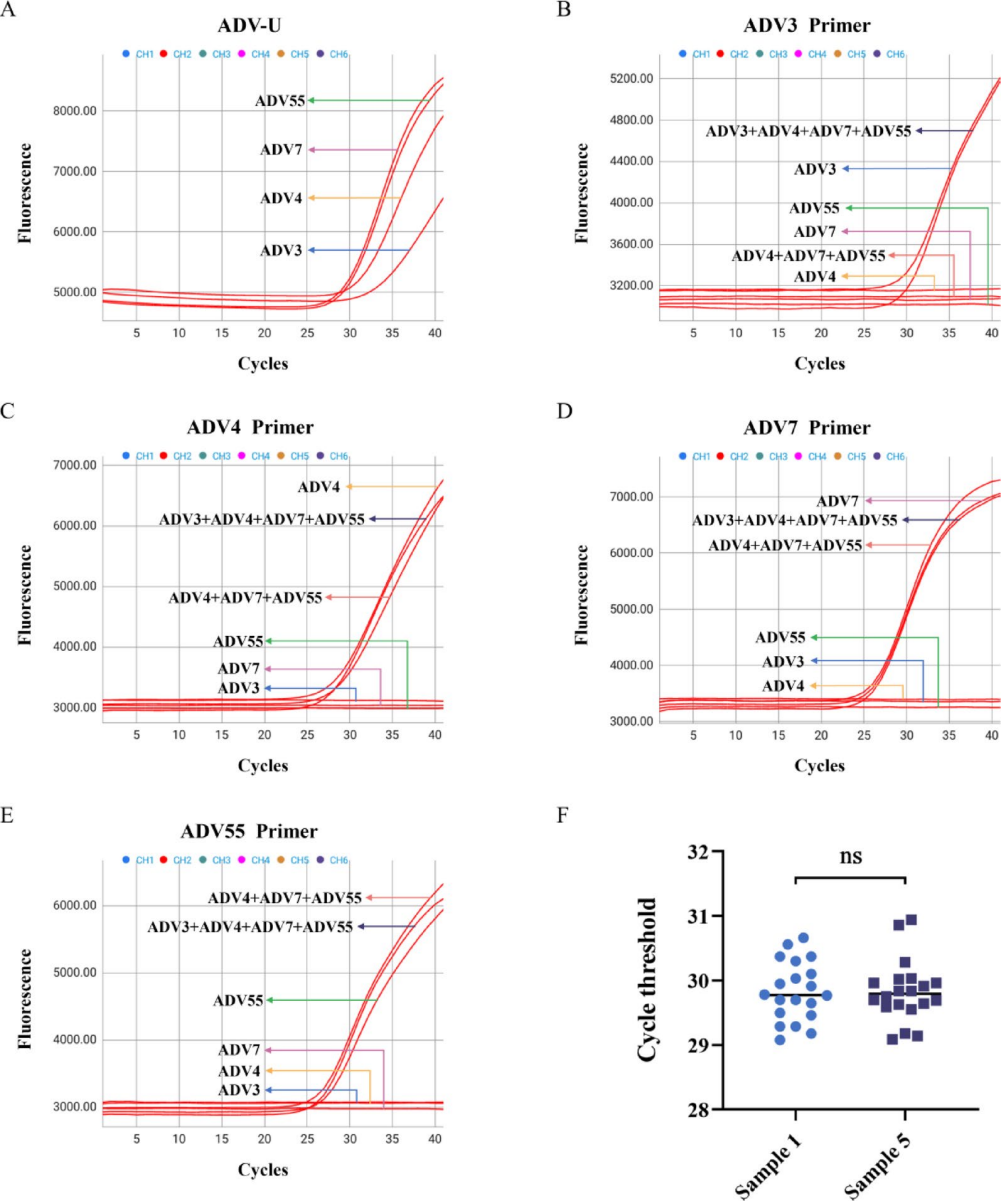
Specificity of the FQ-8B for ADV detection. To verify the specificity of the FQ-8B for ADV, six ADV samples were prepared. Sample 1: low-concentration ADV3 nucleic acid. Sample 2: medium-concentration ADV4 nucleic acid. Sample 3: high-concentration ADV7 nucleic acid. Sample 4: high-concentration ADV55 nucleic acid. Sample 5: mixture of ADV3, ADV4, ADV7, and ADV55 nucleic acids, with concentrations of each ADV corresponding to those in samples 1, 2, 3, and 4, respectively. Sample 6: mixture of ADV4, ADV7, and ADV55 nucleic acids, with concentrations of each ADV corresponding to those in samples 2, 3, and 4, respectively. (**A**) Raw fluorescence curves obtained by analyzing samples 1, 2, 3, and 4 using a universal ADV reagent. Raw fluorescence curves from the analysis of the six samples with primers (**B**) ADV3, (**C**) ADV4, (**D**) ADV7, and (**E**) ADV55. (**F**) Ct values obtained by repeatedly analyzing samples 1 and 5 using ADV3 primers, 20 times each. ADV, adenovirus; Ct, cycle threshold; Statistical significance was calculated using *t* tests; ns *P* > 0.05

The cross-testing results of the 12 pathogens revealed that both the FQ-8B and ABI-Q7 only detected nucleic acids of the corresponding pathogens (Additional File 1: Table S4 and Additional File 6: Raw data) and did not produce false positive results, suggesting that the FQ-8B exhibited superb specificity. Then, to further validate the virus typing capability of the FQ-8B, we prepared six types of ADV samples. Using the ADV universal reagent, four different types of ADV viruses were detected (Fig. 4A), and the primers for ADV3, ADV4, ADV7, and ADV55 specifically detected only the corresponding type of ADV in both single and mixed samples, with no cross-reactivity (Fig. 4B–E), indicating that the FQ-8B demonstrates remarkable virus typing capabilities. Moreover, at the same low concentration, when the FQ-8B was used to detect ADV3 in single samples and in mixed samples containing ADV3, ADV4, ADV7, and ADV55, the Ct values of ADV3 showed no significant difference (Fig. 4F), which further confirmed the specificity of the FQ-8B.

**Fig. 5:**
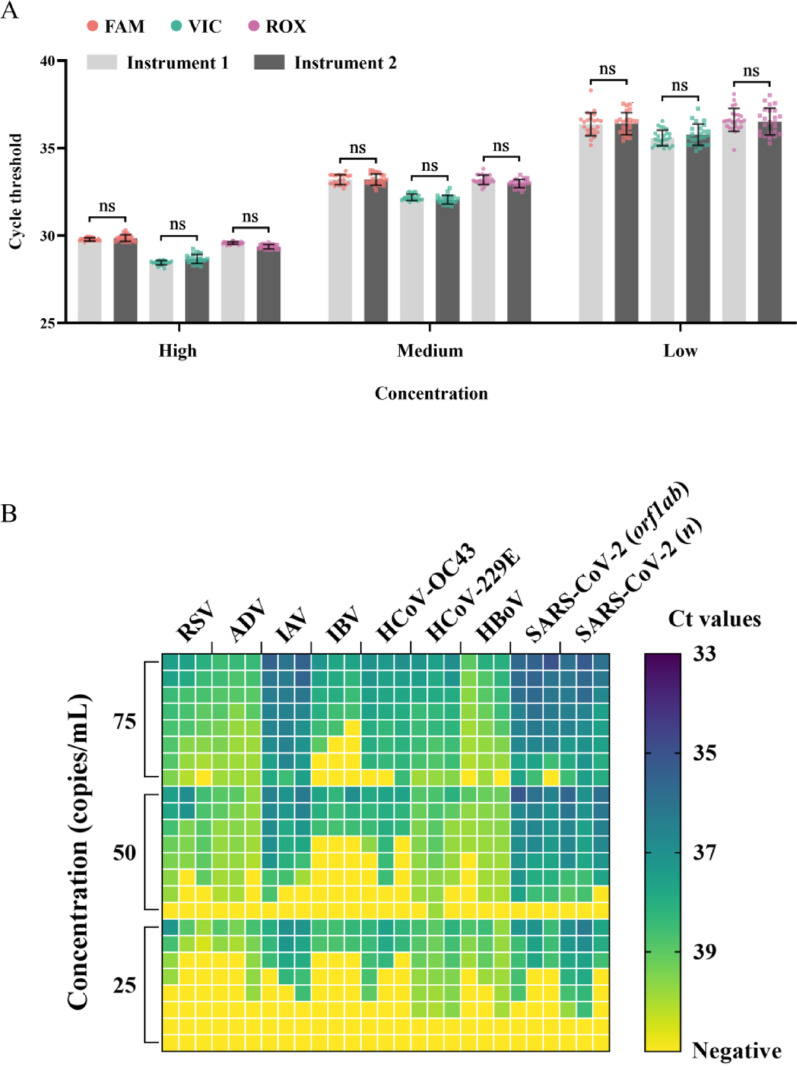
Reproducibility and sensitivity heatmap of the FQ-8B. (**A**) Histogram showing 24 Ct values obtained from testing high, medium, and low concentrations of positive reference samples on the FAM, VIC, and ROX channels using two FQ-8B instruments. (**B**) Nucleic acids from eight viruses were evaluated on the FQ-8B at concentrations of 75, 50, and 25 copies/mL. The recorded Ct values were arranged in ascending order for each replicate set, and results without detectable Ct values were designated as negative. Ct, cycle threshold; RSV, respiratory syncytial virus; ADV, adenovirus; IAV, influenza A virus; IBV, influenza B virus; HCoV-OC43, human coronavirus OC43; HCoV-229E, human coronavirus 229E; HBoV, human bocavirus; SARS-CoV-2, severe acute respiratory syndrome coronavirus 2. Statistical analysis was performed by ANOVA tests; ns *P* > 0.05

The reproducibility results of the FQ-8B (instruments 1 and 2) showed that multiple tests on reference samples of various concentrations using both instruments generated FAM, VIC, and ROX channel amplification curves with good overlap, which consistently improved with higher sample concentrations. This implied minimal inter-well variability among the eight experimental wells per instrument (Additional File 4: Figure S3). An analysis of the CV values showed that when testing the high-concentration templates, the CVs of the three channels (FAM, VIC, and ROX) of instrument 1 were 0.30%, 0.42%, and 0.25%, respectively, while those of instrument 2 were 0.63%, 0.91%, and 0.42%, respectively. When testing the medium-concentration templates, the CVs of the three channels (FAM, VIC, and ROX) of instrument 1 were 0.86%, 0.57%, and 0.80%, respectively, while those of instrument 2 were 0.96%, 0.77%, and 0.71%, respectively. When testing the low-concentration templates, the CVs of the three channels (FAM, VIC, and ROX) of instrument 1 were 1.82%, 1.27%, and 1.79%, respectively, while those of instrument 2 were 1.73%, 1.70%, and 2.10%, respectively. Although the CV values increased with decreasing template concentrations, at the lowest concentration, the CV remained below 3%, implying that the FQ-8B provides highly reproducible results. In addition, there were no significant differences in the results between the two instruments across the three channels at various concentrations (Fig. 5A), indicating consistent reproducibility.

Sensitivity is a vital metric for assessing the detection performance of an instrument, and it is generally defined as the minimum concentration of a sample at which the detection rate is at least 95%, referred to as the 95% limit of detection (LOD_95_). The preliminary sensitivity testing, conducted using each virus at concentrations of 1000, 100, 10, and 0 copies/mL, demonstrated that all three instruments produced negative results on samples with 0 copies/mL, showing no contamination or false positives. The detection rates of the FQ-8B for all eight viruses were over 95% at 100 copies/mL but below 60% at 10 copies/mL, implying an LOD_95_ between 10 and 100 copies/mL. We also noted that the FQ-8B exhibited higher detection rates for RSV and IBV at 100 copies/mL compared to the ABI-Q7 and SLAN-96P (for IBV), confirming that its sensitivity was at least comparable to that of conventional qPCR instruments (Table 1). Subsequently, we further tested the FQ-8B with viral nucleic acid at 75, 50, and 25 copies/mL and found the detection sensitivities for the eight viruses to be as follows: 75 copies/mL for RSV, ADV, IAV, HCoV-229E, and the pseudovirus of SARS-CoV-2, and 100 copies/mL for IBV, HCoV-OC43, and HBoV (Fig. 5B). These results fully confirmed that the FQ-8B detection sensitivity ranged between 75 and 100 copies/mL.

**Table 1:** Analytical sensitivity of the FQ-8B compared to the ABI-Q7 and SLAN-96P

Pathogen and concentration	No. of positives/no. of testing replicates (positive detection rate)		
	FQ-8B	ABI-Q7	SLAN-96P
**RSV**			
1000 copies/mL	24/24 (100%)	24/24 (100%)	24/24 (100%)
100 copies/mL	24/24 (100%)	19/24 (79.2%)	23/24 (95.8%)
10 copies/mL	7/24 (29.2%)	4/24 (16.7%)	7/24 (29.2%)
0 copies/mL	0/24 (0%)	0/24 (0%)	0/24 (0%)
**ADV**			
1000 copies/mL	24/24 (100%)	24/24 (100%)	24/24 (100%)
100 copies/mL	24/24 (100%)	23/24 (95.8%)	24/24 (100%)
10 copies/mL	7/24 (29.2%)	3/24 (12.5%)	4/24 (16.7%)
0 copies/mL	0/24 (0%)	0/24 (0%)	0/24 (0%)
**IAV**			
1000 copies/mL	24/24 (100%)	24/24 (100%)	24/24 (100%)
100 copies/mL	24/24 (100%)	23/24 (95.8%)	23/24 (95.8%)
10 copies/mL	8/24 (33.3%)	11/24 (45.8%)	5/24 (20.8%)
0 copies/mL	0/24 (0%)	0/24 (0%)	0/24 (0%)
**IBV**			
1000 copies/mL	24/24 (100%)	24/24 (100%)	24/24 (100%)
100 copies/mL	23/24 (95.8%)	21/24 (87.5%)	19/24 (79.2%)
10 copies/mL	6/24 (25%)	3/24 (12.5%)	5/24 (20.8%)
0 copies/mL	0/24 (0%)	0/24 (0%)	0/24 (0%)
**HCoV-OC43**			
1000 copies/mL	24/24 (100%)	24/24 (100%)	24/24 (100%)
100 copies/mL	24/24 (100%)	24/24 (100%)	23/24 (95.8%)
10 copies/mL	8/24 (33.3%)	10/24 (41.7%)	6/24 (25%)
0 copies/mL	0/24 (0%)	0/24 (0%)	0/24 (0%)
**HCoV-229E**			
1000 copies/mL	24/24 (100%)	24/24 (100%)	24/24 (100%)
100 copies/mL	24/24 (100%)	24/24 (100%)	24/24 (100%)
10 copies/mL	13/24 (54.2%)	12/24 (50%)	12/24 (50%)
0 copies/mL	0/24 (0%)	0/24 (0%)	0/24 (0%)
**HBoV**			
1000 copies/mL	24/24 (100%)	24/24 (100%)	24/24 (100%)
100 copies/mL	24/24 (100%)	24/24 (100%)	24/24 (100%)
10 copies/mL	9/24 (37.5%)	9/24 (37.5%)	11/24 (45.8%)
0 copies/mL	0/24 (0%)	0/24 (0%)	0/24 (0%)
**Pseudovirus of SARS-CoV-2**			
1000 copies/mL	24/24 (100%)	24/24 (100%)	24/24 (100%)
100 copies/mL	24/24 (100%)	23/24 (100%)	24/24 (100%)
10 copies/mL	1/24 (4.2%)	1/24 (4.2%)	3/24 (12.5%)
0 copies/mL	0/24 (0%)	0/24 (0%)	0/24 (0%)

No., number; RSV, respiratory syncytial virus; ADV, adenovirus; IAV, influenza A virus; IBV, influenza B virus; HCoV-OC43, human coronavirus OC43; HCoV-229E, human coronavirus 229E; HBoV, human bocavirus; SARS-CoV-2, severe acute respiratory syndrome coronavirus 2

### Performance of the FQ-8B in clinical specimen testing

**Fig. 6:**
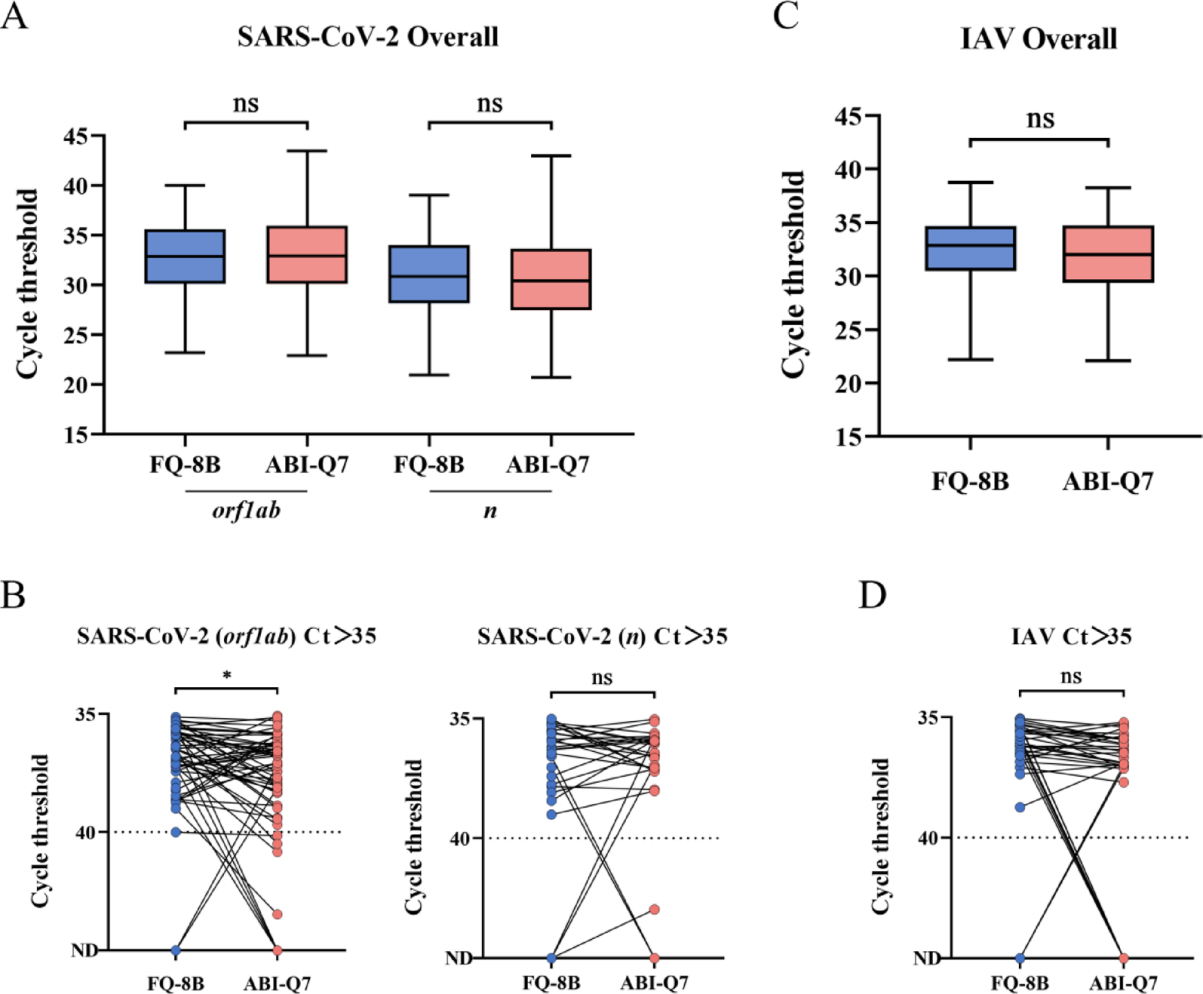
Performance of the FQ-8B for analyzing clinical specimens of SARS-CoV-2 and IAV. (**A**) and (**B**) Nucleic acid from 208 inactivated specimens suspected of SARS-CoV-2 infection was analyzed using the FQ-8B and ABI-Q7, respectively. The results show the overall distribution of the Ct values for the *orf1ab* and *n* genes, along with a comparative analysis of cases in which both instruments yielded Ct values exceeding 35. (**C**) and (**D**) Nucleic acid of 216 inactivated specimens suspected of IAV infection was analyzed using the FQ-8B and ABI-Q7, respectively. The results show the overall distribution of the Ct values, along with a comparative analysis of cases in which both instruments yielded Ct values exceeding 35. Lines within boxes represent medians. Upper and lower boundaries of the boxes represent upper and lower quartiles, respectively. Bars represent minimum and maximum values. Dashed lines indicate the detection limit. SARS-CoV-2, severe acute respiratory syndrome coronavirus 2; IAV, influenza A virus; Ct, cycle threshold; ND, not detected. Statistical significance was calculated using *t* tests; ns *P* > 0.05, *P < 0.05

A total of 208 frozen clinical specimens of suspected SARS-CoV-2 and 216 specimens of suspected IAV were simultaneously tested using the FQ-8B and ABI-Q7. We evaluated the FQ-8B clinical detection performance by comparing Ct values and concordance rates. The results of the FQ-8B for SARS-CoV-2 specimens were as follows: sensitivity of 100.00% (200/200), specificity of 75.00% (6/8), positive predictive value of 99.01% (200/202), negative predictive value of 100.00% (6/6), an overall concordance rate of (200 + 6)/208 = 99.04% (Table 2), and a kappa statistic of 0.852 (*P* < 0.001). The average Ct values of the *orf1ab* and *n* genes were 32.78 and 30.91, respectively, using the FQ-8B, and 32.89 and 30.55, respectively, using the ABI-Q7. Although there was no significant difference in the Ct distributions between the two instruments, the FQ-8B exhibited a lower maximum Ct value than the ABI-Q7 (Fig. 6A). To further evaluate the performance of the FQ-8B with clinical specimens at the LOD, an additional analysis was conducted on specimens with Ct values > 35. The results revealed that the FQ-8B yielded significantly lower average Ct values for the *orf1ab* gene than did the ABI-Q7, signifying superior performance at the LOD. For the *n* gene, no significant difference in Ct values was observed between the two instruments (Fig. 6B).

**Table 2:** Comparison of the FQ-8B and ABI-Q7 in 208 SARS-CoV-2 and 216 IAV clinical specimens

Pathogen	FQ-8B	ABI-Q7		Total
		Positive (+)	Negative (−)	
**SARS-CoV-2**	Positive (+)	200	2	202
	Negative (−)	0	6	6
	Total	200	8	208
**IAV**	Positive (+)	154	8	162
	Negative (−)	2	52	54
	Total	156	60	216

SARS-CoV-2, severe acute respiratory syndrome coronavirus 2; IAV, influenza A virus

The results of the FQ-8B for IAV specimens were as follows: sensitivity of 98.72% (154/156), specificity of 86.67% (52/60), positive predictive value of 95.06% (154/162), negative predictive value of 96.30% (52/54), an overall concordance rate of (154 + 52)/216 = 95.37% (Table 2), and a kappa statistic for the two methods of 0.881 (*P* < 0.001). The average Ct values were 32.18 using the FQ-8B and 31.75 using the ABI-Q7. No significant difference was found in the Ct value distribution between the two instruments. However, the FQ-8B exhibited narrower upper and lower quartiles of the Ct values compared to the ABI-Q7 (Fig. 6C). A further analysis of specimens with Ct > 35 revealed no significant difference in the LOD between the two systems (Fig. 6D).

We identified 12 specimens with discrepancies between the FQ-8B and ABI-Q7 results. Of these, 10 cases (including two SARS-CoV-2 specimens and eight IAV specimens) tested positive on the FQ-8B but negative on the ABI-Q7 (Additional File 1: Table S5), while two IAV specimens tested negative on the FQ-8B but positive on the ABI-Q7 (Additional File 1: Table S6). All these cases had Ct values > 33, and nine cases had Ct values > 35. For such borderline positive results, factors such as the amplification method, fluorescence signal collection, fluorescence processing, and algorithms applied by the different instruments can influence the final detection rates. Overall, the performance of the FQ-8B for analyzing clinical specimens was comparable to that of the conventional ABI-Q7 instrument.

### FQ-8B enables rapid epidemiological detection of multiple respiratory pathogens

To thoroughly evaluate the clinical utility of the FQ-8B system, we developed a 30-min multi-pathogen detection method (FQ-8B rapid test) based on the FQ-8B system. This method enabled the simultaneous detection of 15 respiratory pathogens in a single sample within 30 min. Using this method, we conducted on-site testing of 1227 clinical specimens in non-laboratory settings (Additional File 1: Table S7). A retrospective analysis of the detection results revealed the local epidemiological distribution characteristics of respiratory pathogens during the sampling period, thus demonstrating the practical value of the FQ-8B for real-time rapid screening and multi-pathogen monitoring.

Notably, all 1227 clinical specimens were derived from respiratory specimens collected during patient visits. Because these patients had already been tested for IAV, IBV, and SARS-CoV-2 using the hospital standard qRT-PCR platform, we first compared the results of the FQ-8B rapid test for these three viruses with those obtained from the hospital tests. The results showed a 100% concordance for SARS-CoV-2 (kappa = 1, *P* < 0.001), 99.82% for IAV with two extra positives determined by the FQ-8B (kappa = 0.991, *P* < 0.001), and 98.85% for IBV with 13 additional positives determined by the FQ-8B (kappa = 0.949, *P* < 0.001) (Table 3). These discrepancies originated from samples with low viral loads, and they may be potentially exacerbated by differences in sensitivity among the nucleic acid extraction reagents, diagnostic kits, and qPCR instruments used in the two detection systems.

**Table 3:** Comparison of FQ-8B and standard qRT-PCR detecting IAV, IBV, SARS-CoV-2 in 1227 fever specimens

Pathogen	FQ-8B Rapid test	Standard qRT-PCR test		Total
		Positive (+)	Negative (−)	
**SARS-CoV-2**	Positive (+)	44	0	44
	Negative (−)	0	1183	1183
	Total	44	1183	1227
**IAV**	Positive (+)	132	2	134
	Negative (−)	0	993	993
	Total	132	995	1127
**IBV**	Positive (+)	140	13	153
	Negative (−)	0	974	974
	Total	140	987	1127

SARS-CoV-2, severe acute respiratory syndrome coronavirus 2; IAV, influenza A virus; IBV, influenza B virus; qRT-PCR, quantitative real-time polymerase chain reaction

**Fig. 7:**
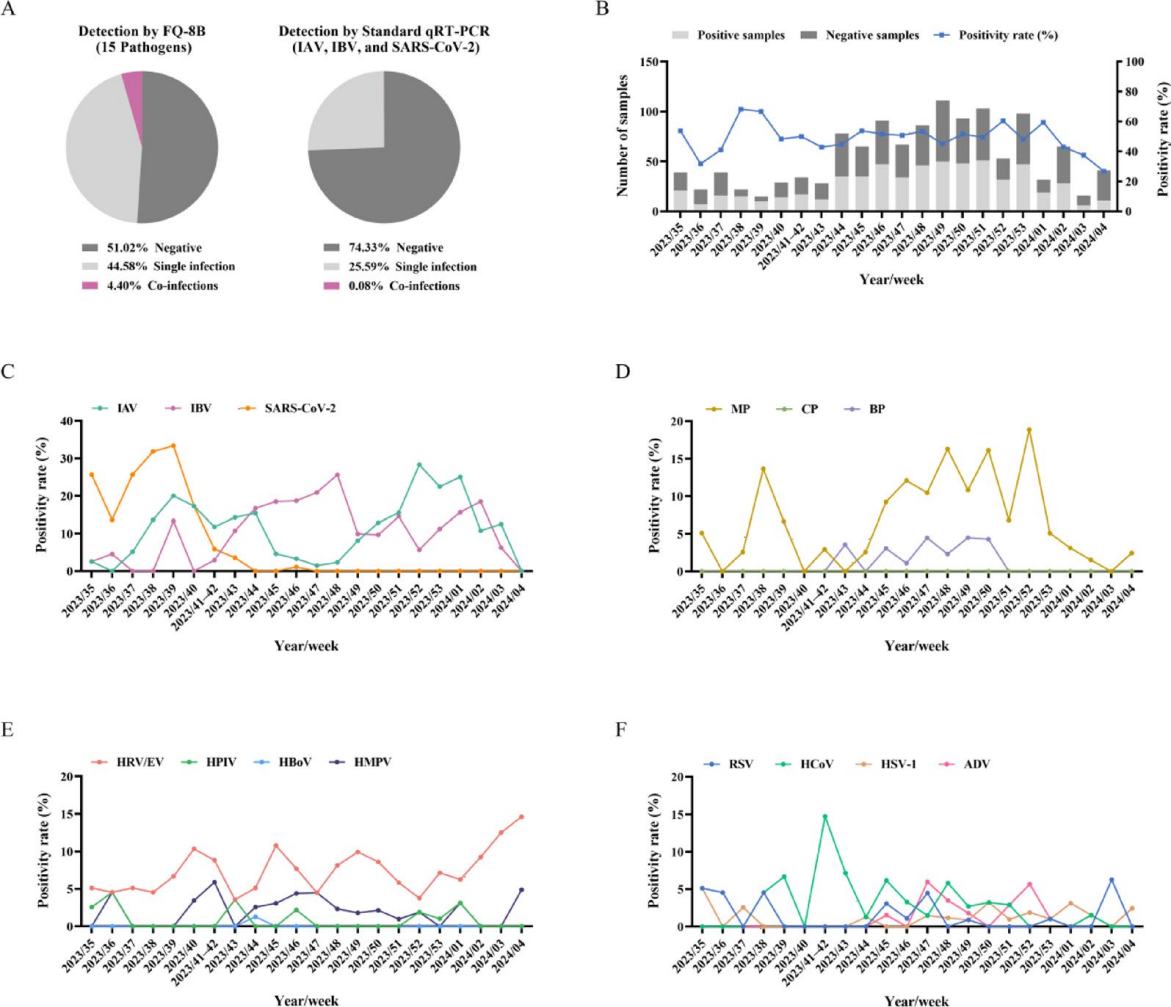
Analysis of the detection data from 1227 respiratory specimens. (**A**) Proportions of negative, single infection, and co-infections results based on the FQ-8B rapid test and hospital standard qRT-PCR test. (**B**) Number of specimens processed each week from week 35 of 2023 to week 4 of 2024, categorized into positive (light gray bars) and negative (dark gray bars) specimens, with the weekly positivity rate depicted by a line graph. Positivity rates of multiple pathogens over 23 consecutive weeks: (**C**) IAV, IBV, and SARS-CoV-2; (**D**) MP, CP, and BP; (**E**) HRV/EV, HPIV, HBoV, and HMPV; and (**F**) RSV, HCoV, HSV-1 and ADV. qRT-PCR, quantitative real-time polymerase chain reaction; IAV, influenza A virus; IBV, influenza B virus; SARS-CoV-2, severe acute respiratory syndrome coronavirus 2; MP, *Mycoplasma pneumoniae*; CP, *Chlamydia pneumoniae*; BP, *Bordetella pertussis*; HRV/EV, human rhinovirus or enterovirus; HPIV, human parainfluenza virus; HBoV, human bocavirus; HMPV, human metapneumovirus; RSV, respiratory syncytial virus; HCoV, human coronavirus; HSV-1, herpes simplex virus type 1; ADV, adenovirus

Owing to its broader pathogen detection coverage, the FQ-8B rapid test identified an additional 286 positive cases compared to the hospital’s triple-virus panel, which had detected only 315 positives (25.67%, including one co-infection). The overall positivity rate with FQ-8B reached 48.98%, including 547 cases of single infections (44.58%) and 54 cases of co-infections with two or more pathogens (4.40%) (Fig. 7A). A total of 30 different co-infection patterns were observed using the FQ-8B assay, among which IBV + MP was the most frequent, occurring in eight cases (0.65%). All pathogens except CP were detected. IBV was the most prevalent pathogen, identified in 153 cases, followed by IAV with 134 cases, MP with 100 cases, HRV/EV with 92 cases, SARS-CoV-2 with 44 cases, and HCoV with 34 cases. The number of positive cases of the remaining pathogens was fewer than 30 (Table 4).

**Table 4:** Common types of respiratory pathogen infections include both single pathogens and co-infections

Infection types	Number	Percentage (%)
**Single infection**	547	44.58
RSV	9	0.73
ADV	14	1.14
IAV	126	10.27
IBV	133	10.84
SARS-CoV-2	41	3.34
HSV-1	10	0.81
HMPV	19	1.55
HRV/EV	75	6.11
HBoV	1	0.08
HPIV	7	0.57
HCoV	25	2.04
MP	74	6.03
CP	0	0.00
BP	13	1.06
**Double infections**	51	4.16
RSV + HMPV	1	0.08
RSV + HRV/EV	2	0.16
RSV + MP	1	0.08
ADV + IAV	1	0.08
ADV + IBV	1	0.08
ADV + HRV/EV	1	0.08
ADV + MP	2	0.16
IAV + IBV	1	0.08
IAV + HSV-1	1	0.08
IAV + HCoV	2	0.16
IAV + MP	2	0.16
IBV + HSV-1	3	0.24
IBV + HMPV	1	0.08
IBV + HCoV	4	0.33
IBV + MP	8	0.65
IBV + BP	1	0.08
SARS-CoV-2 + HRV/EV	1	0.08
SARS-CoV-2 + MP	2	0.16
HSV-1 + HRV/EV	1	0.08
HMPV + HRV/EV	1	0.08
HMPV + HPIV	1	0.08
HMPV + MP	1	0.08
HMPV + BP	1	0.08
HRV/EV + MP	6	0.49
HRV/EV + HCoV	1	0.08
HRV/EV + BP	3	0.24
HCoV + MP	1	0.08
**Triple infections**	2	0.16
IAV + HMPV + MP	1	0.08
IBV + HRV/EV + MP	1	0.08
**Quadruple infections**	1	0.08
HSV-1 + HCoV + MP + BP	1	0.08
**Total**	601	48.98

RSV, respiratory syncytial virus; ADV, adenovirus; IAV, influenza A virus; IBV, influenza B virus; SARS-CoV-2, severe acute respiratory syndrome coronavirus 2; HSV-1, herpes simplex virus type 1; HMPV, human metapneumovirus; HRV/EV, human rhinovirus or enterovirus; HBoV, human bocavirus; HPIV, human parainfluenza virus; HCoV, human coronavirus; MP, *Mycoplasma pneumoniae*; CP, *Chlamydia pneumoniae*; BP, *Bordetella pertussis*

An epidemiological analysis was conducted by aligning the detection results with the specimen collection time (week 35 of 2023 to week 4 of 2024). Because of a public holiday, weeks 41 and 42 were merged into a single time point, as specimen numbers in week 41 were limited. We observed that within the 23-week period, beginning with week 44, the testing volume rose markedly and reached its highest point during week 49. From week 37 to week 4 of the following year, the positivity rate consistently ranged between 41% and 68% (Fig. 7B). During this interval, sequential outbreaks of four pathogens were observed: SARS-CoV-2, IBV, MP, and IAV. Specifically, SARS-CoV-2 exhibited a high positivity rate prior to week 42 and peaked at 33.33%, after which the positivity rate dropped sharply and remained at zero for a prolonged duration. As the incidence of SARS-CoV-2 declined, the rates of IAV and IBV began to increase. Interestingly, from week 38 to week 2 of the following year, IAV and IBV exhibited alternating peak positivity rates, a trend that continued until week 4 of 2024 (Fig. 7C). In the autumn and winter of 2023, China witnessed a significant outbreak of MP, which corresponds to the two peaks observed in week 38 and from weeks 45 to 52 (Fig. 7D) [39]. Additionally, HRV/EV, the most common respiratory virus, had a sustained positivity rate of 5%–10% during this period, with a gradual upward trend observed after the beginning of 2024 (Fig. 7E) [40]. The positivity rate of HCoV showed a transient rise in weeks 41 and 42, followed by a swift decline to below 6% (Fig. 7F). Other pathogens, including BP, HPIV, HMPV, RSV, HSV-1, and ADV, all demonstrated low positivity rates at various times, all less than 10%, and no distinct epidemiological patterns were discerned (Fig. 7D–F).

## Discussion

Since the outbreak of the coronavirus disease 2019 (COVID-19) pandemic in 2019, molecular diagnostic technologies for respiratory pathogens have rapidly evolved. In recent years, numerous innovative NAAT products have emerged, primarily based on PCR testing platforms with integrated extraction and amplification processes, or with all-in-one microfluidic chips [41–43]. These products aim for a “sample in, result out” approach to complete the entire nucleic acid testing process within 1 h or even within 30 min [44]. However, these products face challenges such as limited throughput, bulky instruments, high consumable costs, and restricted reagent compatibility. These limitations impede their widespread use in scientific research and clinical diagnostics. NAATs performed in hospitals are relatively time-consuming, often requiring more than 3–6 h to obtain reliable results, increasing the risk of cross-infection of patients during the waiting period [45]. Antigen self-tests can only detect a minimum viral load of 10^5^–10^6^ copies/mL, which heightens the risk of virus transmission among asymptomatic carriers because of the high false negative rate [46]. Our system, as a complement to standard qRT-PCR clinical testing methods, was designed to be compatible with most marketed diagnostic kits but with a significantly shorter PCR cycle time. We developed a compact, rapid eight-well qPCR instrument with six-channel fluorescence.

Ultra-fast real-time PCR technology primarily relies on extreme reaction conditions or thin-film reaction chambers. The core concepts for shortening the reaction time in these devices involve modifying the chamber medium, reducing the reaction volume to accelerate heat transfer, and optimizing the procedure [47, 48]. Nevertheless, such devices generally incur additional production costs, and most reagents are incompatible with their harsh reaction conditions, making these devices difficult to directly apply in clinical diagnostics. In our study, the common 0.2-mL PCR tube was cycled among three ITMs, leveraging the large temperature differences between the modules to significantly enhance heating and cooling rates. First, optimal temperatures for the HTM and LTM during the PCR cycle were determined using a thermometer probe (Fig. 2A and B). Then, a time-based algorithm was instead implemented to control the heating and cooling processes, which optimized the overshoot at both high and low temperatures compared to the thermometer probe and further shortened the total analysis time (Fig. 2C–E). It should be noted that, because of the lifting speed of the PCR tube holder and the delay in heat transfer, even with the time-based algorithm, a temperature overshoot of approximately 2–3 °C occurred after the tube left the HTM and LTM (Fig. 2F). This also suggested that the FQ-8B may be best suited for DNA polymerases with thermal stability.

The results of qRT-PCR assays depend on various factors, including the timing of specimen collection, specimen type, nucleic acid extraction quality, primer and probe design for viral RNA, and the analytical instruments [49]. Given the unique cyclic amplification strategy of the FQ-8B, we then comprehensively evaluated its performance. When amplifying DNA fragments of varying lengths, the FQ-8B demonstrated comparable results to conventional PCR instruments using a two-step method, but with a significantly shorter processing time (Fig. 3A). In qRT-PCR experiments, the FQ-8B generated typical amplification curves using different primers when amplifying the *gapdh* gene, with Ct values and *T*_*m*_ values showing minimal deviation from those obtained with conventional qPCR instruments, and all six-channel fluorescence of the FQ-8B had high EFF values (Fig. 3). Moreover, the FQ-8B exhibited excellent specificity, reproducibility, and sensitivity. Non-specific amplifications were not observed, and the instrument demonstrated excellent viral typing capabilities (Fig. 4). In repeated experiments using high-, medium-, and low-concentration templates, the CV values were all below 3%, indicating minimal inter-well and inter-instrument variability and high experimental reproducibility (Fig. 5A). The LOD_95_ of the FQ-8B of eight viruses ranged from 75 to 100 copies/mL (Fig. 5B). The FQ-8B demonstrated satisfactory performance in clinical specimen testing of SARS-CoV-2 and IAV, indicating its ability to fulfill clinical testing requirements (Fig. 6). These findings showed that the three-ITM design concept and a time-based algorithm to control the temperature rise and fall enabled effective PCR cyclic amplification.

Thereafter, we combined the FQ-8B with a multiplexed detection reagent to establish a 30-min multi-pathogen detection method. Using this method, we tested 1227 respiratory specimens, and obtained a positive rate of 48.98%, which was higher than the 25.67% positive rate from the hospital’s triple-virus panel (Fig. 7A). The 286 additional positive specimens detected were mostly beyond the scope of the hospital’s triple-virus test and involved infections by other pathogens. The 48.98% positive rate achieved by our method also exceeded a previously reported 35.8% positive rate of another study [50]; its co-infection rate was 4.40%, greater than the 2.2% rate described by Krishnan but close to the 5.0% rate noted by Kim [51, 52]. These results indicated that the FQ-8B rapid test enabled more comprehensive pathogen screening within a short time, and therefore may provide strong support for clinical diagnosis, treatment, and precise medication. Furthermore, by analyzing the local pathogen distribution characteristics during the sampling period, we identified a sequential trend of a high prevalence of four pathogens (Fig. 7C–F). Among them, SARS-CoV-2, as an emerging infectious virus, exhibited an epidemic trend that may be related to the antibody levels present in the local population and the emergence of newly mutated variants of the virus [53, 54]. IAV and IBV, as seasonal epidemic viruses, showed a high prevalence in winter, consistent with previous reports [55]. The MP outbreak mainly occurred from August to January, with a positivity rate of approximately 6.16% and a particularly high peak in November in our study [56]. Conversely, RSV, a winter-prevalent virus, did not show high positivity in our results [57]. HRV, traditionally more common in spring or autumn, demonstrated an increasing winter positivity trend in this study [2, 58]. It is noteworthy that data collection began in the ninth month after the end of non-pharmaceutical interventions for the COVID-19 pandemic. These epidemiological statistics therefore provide an important references for monitoring the prevalence of multiple pathogens and formulating epidemic prevention strategies in the post-COVID-19 era [59, 60]. However, the study used a single-center, retrospective approach, focusing on one hospital, which is inherently limited. Nevertheless, the 30-min multi-pathogen detection method established by the FQ-8B demonstrated advantages such as rapid detection, broad target coverage, and instrument portability, facilitating its clinical use across different types of healthcare institutions. In addition, compared to existing respiratory pathogen rapid diagnostic platforms (Additional File 1: Table S8), FQ-8B offers greater reagent versatility and lower detection costs, rendering it more practical for use in resource-limited laboratories or primary healthcare settings [41, 42, 61–63].

However, the FQ-8B exhibited certain limitations. For example, its throughput is limited, making it unsuitable for large-scale screening applications. During the movement of the PCR tube among the ITMs, precise alignment between the tube walls and the modules is essential, thus requiring strict manufacturing precision and long-term operational stability. The key to enhancing the instrument speed lies in efficient heating and cooling processes, which significantly reduced the processing time over multiple amplification cycles. Nevertheless, the melting curve step remains time-consuming as it requires gradual heating within a single module. Notably, studies have suggested that accelerated melting rates may paradoxically improve the resolution of short amplicons, implying that optimized melting speeds could streamline this step [64]. The emerging multicolor melting curve analysis enables the simultaneous detection of dozens of targets in a single reaction by using multiplexed fluorescence channels. Yet, these methods require multiple extension temperatures or post-denaturation fluorescence acquisition, which are incompatible with the design of the FQ-8B, limiting its applicability to these advanced approaches [65, 66].

## Conclusion

In summary, this study developed a novel method for PCR temperature control, which employed a time-based algorithm to cycle reaction tubes among three ITMs. By utilizing the large temperature differences between these modules, the heating and cooling rates were significantly improved. Based on this technology, a six-channel fluorescence qPCR instrument FQ-8B was developed that operated with common 0.2-mL PCR tubes. Its advantages over traditional systems include cost-effectiveness, portability, user-friendly operation, superior performance, and fast processing. The FQ-8B overcomes the limitations of conventional PCR platforms to enable precise molecular diagnostics outside specialized laboratory settings, and therefore, it is particularly useful for resource-constrained operations. The system facilitates timely infection control and evidence-based therapeutic decisions by rapidly detecting nucleic acids from multiple pathogens. It provides a reliable platform for clinical management of ARIs, and it has significant potential to improve on-site diagnostics.

## Supplementary Information

Supplementary Material 1

Supplementary Material 2

Supplementary Material 3

Supplementary Material 4

Supplementary Material 5

Supplementary Material 6

## Data Availability

All data generated or analyzed during this study are included in this published article and its supplementary information files.

## Abbreviations

ARIs: Acute respiratory infections
IAV: Influenza A virus
SARS-CoV-2: Severe acute respiratory syndrome coronavirus 2
MP: Mycoplasma pneumoniae
NAATs: Nucleic acid amplification tests
qRT-PCR: Quantitative real-time polymerase chain reaction
CRISPR: Clustered regularly interspaced short palindromic repeat
qPCR: Quantitative PCR
bp: Base pairs
ITMs: Independent temperature modules
RSV: Respiratory syncytial virus
ADV: Adenovirus
IBV: Influenza B virus
HSV-1: Herpes simplex virus type 1
HPIV: Human parainfluenza virus
HCoV-OC43: Human coronavirus OC43
HCoV-229E: Human coronavirus 229E
HCoV-NL63: Human coronavirus NL63
HRV: Human rhinovirus
HBoV: Human bocavirus
HMPV: Human metapneumovirus
HTM: High-temperature module
LTM: Low-temperature module
MTM: Medium-temperature module
gapdh: glyceraldehyde-3-phosphate dehydrogenase
EV: Enterovirus
Ct: Cycle threshold
T_m_: Melting temperature
EFF: Amplification efficiency
DEPC: Diethyl pyrocarbonate
CV: Coefficient of variation
CP: Chlamydia pneumoniae
BP: Bordetella pertussis
LOD_95_: 95% limit of detection
COVID-19: Coronavirus disease 2019
