# Supplementary material for: Development and clinical application of a rapid qPCR instrument featuring three independent temperature modules and a time-based algorithm for respiratory pathogen diagnosis
Source: Virol J. 2025 Nov 21;22:381. doi: 10.1186/s12985-025-03003-2 (PMC12639651; doi:10.1186/s12985-025-03003-2)
Supplement: Supplementary file 1 — Supplementary Material 1 [file 12985_2025_3003_MOESM1_ESM.docx]

**Table S1** TaqMan real-time PCR kits used in this study

| **Testing targets** | **TaqMan Real-Time PCR kits** | **Manufacturer** |
| --- | --- | --- |
| Atto425, FAM, VIC, ROX, CY5, Quasar 705 | Respiratory syncytial virus, adenovirus, influenza A, influenza B and cytomegalovirus Nucleic Acid Diagnostic kit | Huirui Biotechnology Co., Ltd., Zhuhai, China |
| ADV | Human adenovirus Nucleic Acid Diagnostic kit | HuYanSuo Medical Technology Co., Ltd., Guangzhou, China |
| RSV | Respiratory syncytial virus Nucleic Acid Diagnostic kit | HuYanSuo Medical Technology Co., Ltd., Guangzhou, China |
| SARS-CoV-2 | 2019-nCoV Nucleic Acid Diagnostic kit | Sansure Biotech Inc., Changsha, China |
| IAV | SARS-CoV-2 and influenza A/B Virus Nucleic Acid Diagnostic kit | Sansure Biotech Inc., Changsha, China |
| IBV | Influenza B Virus Nucleic Acid Diagnostic kit | Huirui Biotechnology Co., Ltd., Zhuhai, China |
| HCoV-OC43 | Human coronavirus OC43 Nucleic Acid Diagnostic kit | Huirui Biotechnology Co., Ltd., Zhuhai, China |
| HCoV-229E | Human coronavirus 229E Nucleic Acid Diagnostic kit | Huirui Biotechnology Co., Ltd., Zhuhai, China |
| HCoV-NL63 | Human coronavirus NL63 Nucleic Acid Diagnostic kit | Huirui Biotechnology Co., Ltd., Zhuhai, China |
| HBoV | Human Bocavirus Nucleic Acid Diagnostic kit | Huirui Biotechnology Co., Ltd., Zhuhai, China |
| HRV | Human rhinovirus Nucleic Acid Diagnostic kit | Huirui Biotechnology Co., Ltd., Zhuhai, China |
| HPIV-1 | Human parainfluenza virus type 1/2/3/4 Nucleic Acid Diagnostic kit | Huirui Biotechnology Co., Ltd., Zhuhai, China |
| HSV-1 | Herpes simplex virus type 1 Nucleic Acid Diagnostic kit | Huirui Biotechnology Co., Ltd., Zhuhai, China |
| HMPV | Human metapneumovirus Nucleic Acid Diagnostic kit | HuYanSuo Medical Technology Co., Ltd., Guangzhou, China |
| MP | Mycoplasma pneumoniae DNA Diagnostic kit | HuYanSuo Medical Technology Co., Ltd., Guangzhou, China |
| Fifteen respiratory pathogens | Fifteen respiratory pathogens multiplex Nucleic Acid Diagnostic kit | Huirui Biotechnology Co., Ltd., Zhuhai, China |

Abbreviations: ADV, adenovirus; RSV, respiratory syncytial virus; SARS-CoV-2, severe acute respiratory syndrome coronavirus 2; IAV, influenza A virus; IBV, influenza B virus; HCoV-OC43, human coronavirus OC43; HCoV-229E, human coronavirus 229E; HCoV-NL63, human coronavirus NL63; HBoV, human bocavirus; HRV, human rhinovirus; HPIV-1, human parainfluenza virus type 1; HSV-1, herpes simplex virus type 1; HMPV, human metapneumovirus; MP, *Mycoplasma pneumoniae*.

**Table S2** Primers used in this study

| **Primer name** | **Description** | **Primer sequence (5’–3’)** |
| --- | --- | --- |
| 100-FP | 100 bp PCR primer, forward | TGCTTACTGGCTTATCGAAATT |
| 100-RP | 100 bp PCR primer, reverse | CTGTTTTAAGAACCCGGGATA |
| 200-FP | 200 bp PCR primer, forward | TATCCCGGGTTCTTAAAACAG |
| 200-RP | 200 bp PCR primer, reverse | CAGTCCGGGGAAACAGAA |
| 500-FP | 500 bp PCR primer, forward | GCGGTTGAAGGAGAAAACG |
| 500-RP | 500 bp PCR primer, reverse | GGTGGTCACAGAAATCAAAG |
| 1000-FP | 1000 bp PCR primer, forward | GCCTTTTGATTCTGCCTTGAA |
| 1000-RP | 1000 bp PCR primer, reverse | TCGATGACTGCTCACCTG |
| 2000-FP | 2000 bp PCR primer, forward | AACGCTAGTGACGAGAGTAT |
| 2000-RP | 2000 bp PCR primer, reverse | TCATCCATTACTGTAACCACC |
| 5064-FP | 5064 bp PCR primer, forward | ACAGTACAGCAGAGACTACT |
| 5064-RP | 5064 bp PCR primer, reverse | GTTCTTTCCGCCTCAGAAG |
| Primer 1-FP | *gapdh* Real-time PCR primer 1, forward | TGCCCTCAACGACCACTTTG |
| Primer 1-RP | *gapdh* Real-time PCR primer 1, reverse | CGCCAGACCCTGCACTTTTT |
| Primer 2-FP | *gapdh* Real-time PCR primer 2, forward | CATGCCTTAAGCCAGGCCAG |
| Primer 2-RP | *gapdh* Real-time PCR primer 2, reverse | TATGTGAGCAGCCCTAGGCC |
| Primer 3-FP | *gapdh* Real-time PCR primer 3, forward | AGTGAGTGGAAGACAGAATGG |
| Primer 3-RP | *gapdh* Real-time PCR primer 3, reverse | GTTACCATATACCCAAGGGAGC |
| Primer 4-FP | *gapdh* Real-time PCR primer 4, forward | CGCTCTGCGGGGTCACGTG |
| Primer 4-RP | *gapdh* Real-time PCR primer 4, reverse | CCGCAGGCGCAGCATCCG |
| ADV3-FP | ADV3 Real-time PCR primer, forward | TATGTAACGCTAATGGGAGC |
| ADV3-RP | ADV3 Real-time PCR primer, reverse | ACCTCTTGCACTAAAGTGAG |
| ADV4-FP | ADV4 Real-time PCR primer, forward | TGTATGATCTGGAGGTGGAT |
| ADV4-RP | ADV4 Real-time PCR primer, reverse | CTTTAAGCCAGGGGATCTTT |
| ADV7-FP | ADV7 Real-time PCR primer, forward | ACAAAACCTCCAGGATACG |
| ADV7-RP | ADV7 Real-time PCR primer, reverse | CCCGAGAACTGAGTGTTCTA |
| ADV55-FP | ADV55 Real-time PCR primer, forward | GTTTCCACTACCACATTCCT |
| ADV55-RP | ADV55 Real-time PCR primer, reverse | CCGTTGCAGAAAAATCTACC |

Abbreviations: *gapdh*, glyceraldehyde-3-phosphate dehydrogenase; ADV, adenovirus.

**Table S3** Standard and rapid PCR amplification procedures

| **Step** | **Temperature (°C)** | **Time (s)** | **Number of cycles** |
| --- | --- | --- | --- |
| **Standard three-temperature cycling** | | | |
| Pre-degeneration | 98 | 120 | 1 |
| Degeneration | 98 | 10 | 35 |
| Annealing | 60 | 15 | 35 |
| Extension | 72 | 3/6/15/30/60/150 | 35 |
| **Rapid two-temperature cycling** | | | |
| Pre-degeneration | 98 | 30 | 1 |
| Degeneration | 98 | 1 | 35 |
| Annealing and Extension | 60 | 1/2/5/20/45/120 | 35 |

**Table S4** The specificity performance of the FQ-8B in comparison to an ABI-Q7

| **Viral nucleic acid** | **Ct values**  **(mean±SD)** | |
| --- | --- | --- |
|  | FQ-8B | ABI-Q7 |
| RSV | 30.24±0.19 | 30.58±0.19 |
| ADV | 27.72±0.02 | 28.33±0.11 |
| IAV | 27.61±0.05 | 27.51±0.28 |
| HSV-1 | 28.67±0.37 | 28.93±0.84 |
| HCoV-229E | 30.42±0.07 | 31.06±0.16 |
| HCoV-OC43 | 27.62±0.04 | 27.01±0.14 |
| HCoV-NL63 | 29.14±0.62 | 29.39±0.11 |
| HRV | 28.68±0.11 | 29.15±0.09 |
| HBoV | 27.02±0.14 | 27.52±0.11 |
| HMPV | 26.49±0.55 | 26.62±0.21 |
| HPIV-1 | 26.87±0.04 | 27.18±0.03 |
| MP | 33.32±0.29 | 33.81±0.40 |

Abbreviations: Ct, cycle threshold; SD, standard deviation; RSV, respiratory syncytial virus; ADV, adenovirus; IAV, influenza A virus; HSV-1, herpes simplex virus type 1; HCoV-229E, human coronavirus 229E; HCoV-OC43, human coronavirus OC43; HCoV-NL63, human coronavirus NL63; HRV, human rhinovirus; HBoV, human bocavirus; HMPV, human metapneumovirus; HPIV-1, human parainfluenza virus type 1; MP, *Mycoplasma pneumoniae*.

**Table S5** Clinical data of SARS-CoV-2 specimens

| Serial No. | Specimen | FQ-8B  (Ct values) | | | ABI-Q7  (Ct values) | | |
| --- | --- | --- | --- | --- | --- | --- | --- |
|  |  | SARS-CoV-2 (*orf1ab*) | IC (*rnase p*) | SARS-CoV-2 (*n*) | SARS-CoV-2 (*orf1ab*) | IC (*rnase p*) | SARS-CoV-2 (*n*) |
| 1 | 10001 | 32.24 | 24.62 | 29.94 | 31.75 | 25.02 | 28.83 |
| 2 | 10002 | 40.00 | 25.00 | 36.20 | 40.14 | 25.44 | 35.78 |
| 3 | 10008 | 31.02 | 28.20 | 27.78 | 30.75 | 28.58 | 27.00 |
| 4 | 10009 | 27.71 | 29.05 | 25.14 | 26.67 | 29.60 | 24.19 |
| 5 | 10018 | 28.92 | 27.76 | 26.99 | 27.39 | 27.78 | 26.05 |
| 6 | 10020 | - | 32.39 | - | - | 32.78 | - |
| 7 | 10026 | 27.97 | 28.43 | 24.91 | 29.42 | 29.02 | 26.29 |
| 8 | 10033 | 34.75 | 30.05 | 31.52 | 32.30 | 29.53 | 29.62 |
| 9 | 10034 | 36.84 | 29.81 | 35.50 | 39.68 | 30.70 | 35.04 |
| 10 | 10035 | 29.94 | 26.00 | 26.93 | 29.85 | 26.64 | 26.18 |
| 11 | 10036 | 32.71 | 29.05 | 30.65 | 32.43 | 29.94 | 30.21 |
| 12 | 10037 | 30.48 | 27.84 | 28.11 | 30.10 | 28.54 | 27.23 |
| 13 | 10038 | 27.47 | 26.00 | 24.45 | 27.09 | 27.08 | 23.88 |
| 14 | 10039 | 31.86 | 32.09 | 29.65 | 31.38 | 33.20 | 29.00 |
| 15 | 10040 | 37.43 | 26.10 | - | 37.76 | 27.41 | 35.79 |
| 16 | 10041 | 28.46 | 27.03 | 25.46 | 27.85 | 27.18 | 24.48 |
| 17 | 10042 | 29.96 | 27.06 | 27.75 | 30.18 | 27.61 | 27.44 |
| 18 | 10043 | 36.87 | 26.25 | 33.70 | 36.49 | 27.18 | 33.55 |
| 19 | 10045 | 25.46 | 24.63 | 22.06 | 24.88 | 25.49 | 21.64 |
| 20 | 10046 | 25.55 | 26.51 | 22.76 | 25.17 | 27.69 | 22.21 |
| 21 | 10047 | 36.98 | 24.20 | 35.87 | 37.44 | 25.23 | 37.11 |
| 22 | 10048 | 28.47 | 27.70 | 26.41 | 28.24 | 28.63 | 25.83 |
| 23 | 10049 | 37.24 | 27.73 | 34.45 | 36.77 | 28.69 | 34.24 |
| 24 | 10050 | 28.90 | 28.08 | 26.74 | 28.65 | 28.69 | 26.15 |
| 25 | 10051 | 28.54 | 27.68 | 26.55 | 29.04 | 28.42 | 26.15 |
| 26 | 10052 | 28.88 | 27.85 | 26.73 | 28.98 | 28.37 | 26.10 |
| 27 | 10053 | 33.71 | 23.17 | 31.14 | 33.69 | 24.44 | 30.44 |
| 28 | 10054 | 33.44 | 27.64 | 32.09 | 34.09 | 28.75 | 31.01 |
| 29 | 10055 | 27.58 | 24.77 | 25.72 | 27.09 | 25.82 | 25.13 |
| 30 | 10057 | 35.13 | 25.20 | 33.45 | 35.29 | 26.53 | 33.65 |
| 31 | 10058 | 33.97 | 23.75 | 32.67 | 34.91 | 24.99 | 31.94 |
| 32 | 10059 | 30.14 | 26.70 | 28.58 | 30.14 | 27.18 | 28.31 |
| 33 | 10069 | 32.50 | 27.61 | 28.17 | 33.36 | 28.03 | 27.98 |
| 34 | 10070 | 33.43 | 29.01 | 30.67 | 34.38 | 29.98 | 30.44 |
| 35 | 10071 | 31.65 | 25.61 | 28.07 | 32.24 | 26.94 | 27.86 |
| 36 | 10072 | 32.28 | 25.49 | 29.19 | 32.52 | 26.96 | 29.20 |
| 37 | 10073 | 29.04 | 27.18 | 26.17 | 29.09 | 28.14 | 26.11 |
| 38 | 10074 | 31.52 | 28.15 | 28.69 | 31.47 | 29.39 | 28.46 |
| 39 | 10075 | 27.03 | 26.46 | 24.48 | 26.96 | 27.08 | 24.17 |
| 40 | 10076 | 32.77 | 26.66 | 29.67 | 32.91 | 27.13 | 29.49 |
| 41 | 10077 | 32.24 | 26.71 | 30.49 | 32.60 | 27.21 | 30.49 |
| 42 | 10078 | 38.62 | 28.50 | 34.73 | 38.88 | 29.29 | 35.32 |
| 43 | 10079 | 31.81 | 29.17 | 29.59 | 32.43 | 30.03 | 29.75 |
| 44 | 10080 | 31.93 | 27.01 | 29.17 | 32.46 | 27.81 | 29.18 |
| 45 | 10081 | 35.58 | 25.25 | 33.10 | 36.36 | 26.38 | 33.67 |
| 46 | 10082 | 35.82 | 25.78 | 36.29 | 38.97 | 26.99 | 35.84 |
| 47 | 10083 | 36.08 | 24.52 | 32.95 | 36.51 | 25.44 | 33.47 |
| 48 | 10084 | 37.23 | 27.25 | 34.91 | 40.83 | 27.76 | 36.27 |
| 49 | 10085 | 29.07 | 26.58 | 26.46 | 28.78 | 27.08 | 26.49 |
| 50 | 10086 | 30.07 | 30.09 | 27.06 | 30.52 | 31.70 | 27.02 |
| 51 | 10087 | 30.82 | 23.94 | 28.65 | 31.29 | 25.91 | 28.54 |
| 52 | 10088 | 25.08 | 26.14 | 21.84 | 25.45 | 28.36 | 21.91 |
| 53 | 10089 | 31.55 | 28.01 | 28.49 | 32.35 | 29.75 | 28.81 |
| 54 | 10090 | 30.93 | 28.13 | 28.79 | 31.09 | 29.42 | 28.72 |
| 55 | 10091 | 27.43 | 23.77 | 25.76 | 27.39 | 25.03 | 25.80 |
| 56 | 10092 | 30.09 | 26.01 | 26.99 | 30.21 | 26.31 | 26.60 |
| 57 | 10093 | 33.17 | 30.20 | 30.81 | 33.86 | 30.76 | 30.62 |
| 58 | 10094 | 29.91 | 25.13 | 26.42 | 30.43 | 26.48 | 26.30 |
| 59 | 10095 | 33.82 | 25.53 | 32.45 | 34.56 | 27.45 | 32.94 |
| 60 | 10096 | 35.91 | 26.17 | 34.91 | 40.49 | 28.24 | 34.68 |
| 61 | 10097 | 32.18 | 23.89 | 30.57 | 32.81 | 25.61 | 30.31 |
| 62 | 10100 | 32.54 | 28.46 | 30.22 | 32.71 | 30.08 | 30.35 |
| 63 | 10101 | 28.94 | 25.08 | 26.60 | 29.38 | 26.30 | 26.59 |
| 64 | 10102 | 34.20 | 24.15 | 32.84 | 35.35 | 25.31 | 34.21 |
| 65 | 10103 | 34.07 | 26.18 | 31.59 | 34.27 | 27.01 | 31.00 |
| 66 | 10105 | 26.44 | 26.03 | 24.18 | 26.76 | 27.27 | 24.14 |
| 67 | 10106 | 29.13 | 26.53 | 27.00 | 29.19 | 27.67 | 29.07 |
| 68 | 10107 | 37.26 | 24.96 | 35.97 | - | 27.45 | 36.57 |
| 69 | 10170 | 30.60 | 25.72 | 28.07 | 31.02 | 27.55 | 28.04 |
| 70 | 10172 | 33.16 | 27.48 | 31.16 | 34.11 | 29.04 | 31.13 |
| 71 | 10173 | 34.25 | 24.97 | 32.54 | 35.44 | 26.90 | 32.97 |
| 72 | 10174 | 23.18 | 24.46 | 20.96 | 22.90 | 25.26 | 20.69 |
| 73 | 10175 | 25.54 | 24.28 | 21.88 | 25.76 | 24.51 | 21.47 |
| 74 | 10176 | 34.18 | 26.63 | 31.80 | 36.01 | 27.62 | 32.38 |
| 75 | 10177 | 33.52 | 25.93 | 31.62 | 34.67 | 27.25 | 31.08 |
| 76 | 10178 | 34.10 | 26.48 | 30.29 | 35.13 | 28.04 | 30.38 |
| 77 | 10179 | 35.80 | 28.36 | 35.24 | 38.87 | 29.75 | 37.16 |
| 78 | 10180 | 34.39 | 30.64 | 31.12 | 35.42 | 31.05 | 31.29 |
| 79 | 10181 | 35.28 | 28.91 | 30.81 | 37.46 | 30.14 | 30.69 |
| 80 | 10182 | 38.67 | 26.14 | 37.84 | 39.39 | 27.21 | 37.97 |
| 81 | 10183 | 38.67 | 26.21 | 35.57 | 35.84 | 26.92 | 36.47 |
| 82 | 10184 | 28.47 | 26.62 | 25.05 | 27.33 | 27.26 | 24.39 |
| 83 | 10185 | 35.72 | 27.15 | 33.83 | 37.93 | 28.21 | 32.54 |
| 84 | 10186 | 34.68 | 25.21 | 32.77 | 35.33 | 26.34 | 31.33 |
| 85 | 10187 | 38.75 | 24.75 | 34.45 | 36.12 | 26.32 | 33.25 |
| 86 | 10188 | 38.95 | 22.96 | 34.84 | 34.88 | 23.98 | 33.44 |
| 87 | 10189 | 37.05 | 25.73 | 35.57 | 35.08 | 26.35 | 33.68 |
| 88 | 10190 | 28.50 | 26.63 | 25.52 | 25.93 | 26.26 | 24.28 |
| 89 | 10191 | 37.22 | 27.90 | 31.98 | 36.44 | 28.10 | 30.52 |
| 90 | 10192 | 31.00 | 27.04 | 28.47 | 30.05 | 28.07 | 27.12 |
| 91 | 10193 | 32.42 | 31.05 | 28.65 | 31.70 | 31.82 | 27.51 |
| 92 | 10194 | 37.26 | 29.22 | 35.23 | 36.68 | 30.76 | 36.06 |
| 93 | 10195 | 33.94 | 25.62 | 32.30 | 33.48 | 27.20 | 31.27 |
| 94 | 10196 | 35.28 | 27.95 | 35.65 | 33.22 | 29.81 | 33.90 |
| 95 | 10197 | 33.03 | 28.01 | 31.19 | 31.60 | 29.46 | 30.09 |
| 96 | 10198 | 36.33 | 24.51 | 33.91 | 36.23 | 25.39 | 33.22 |
| 97 | 10199 | 35.89 | 27.10 | 35.99 | 35.86 | 27.79 | 35.14 |
| 98 | 10200 | - | 27.60 | 37.76 | - | 29.38 | 36.44 |
| 99 | 10201 | 36.86 | 26.02 | - | 36.52 | 27.91 | - |
| 100 | 10202 | 32.64 | 23.65 | 30.48 | 31.69 | 25.07 | 29.11 |
| 101 | 10203 | 38.22 | 24.58 | 36.35 | 36.09 | 26.58 | 34.76 |
| 102 | 10204 | - | 27.74 | 35.18 | - | 28.98 | 35.62 |
| 103 | 10206 | 33.29 | 26.39 | 31.20 | 32.92 | 26.76 | 29.81 |
| 104 | 10207 | 35.24 | 24.61 | 33.02 | 35.91 | 25.64 | 31.94 |
| 105 | 10208 | 32.63 | 26.68 | 29.09 | 32.15 | 27.62 | 27.61 |
| 106 | 10209 | 35.57 | 26.84 | 34.00 | 35.80 | 28.66 | 33.28 |
| 107 | 10210 | - | 26.54 | - | - | 27.21 | - |
| 108 | 10212 | 34.64 | 26.49 | 33.16 | 35.04 | 28.57 | 33.22 |
| 109 | 10213 | 33.21 | 27.92 | 31.71 | 32.73 | 30.10 | 30.66 |
| 110 | 10214 | 34.65 | 24.96 | 34.22 | 35.97 | 27.27 | 33.21 |
| 111 | 10215 | 29.04 | 27.66 | 26.19 | 27.91 | 29.26 | 25.39 |
| 112 | 10216 | 38.66 | 28.40 | - | 37.06 | 29.11 | 34.55 |
| 113 | 10217 | 30.18 | 27.06 | 28.26 | 28.71 | 27.18 | 27.05 |
| 114 | 10218 | 29.58 | 27.55 | 26.57 | 28.98 | 27.89 | 25.21 |
| 115 | 10219 | 37.42 | 28.51 | 35.28 | 35.95 | 29.42 | 36.64 |
| 116 | 10220 | - | 22.47 | 37.22 | - | 23.63 | 34.96 |
| 117 | 10221 | 34.96 | 28.07 | 34.19 | 36.90 | 29.63 | 34.42 |
| 118 | 10222 | - | 25.70 | 35.24 | 37.14 | 27.04 | 35.97 |
| 119 | 10225 | 32.31 | 24.69 | 30.03 | 32.33 | 25.98 | 28.93 |
| 120 | 10226 | 28.45 | 29.06 | 25.17 | 27.24 | 29.68 | 23.81 |
| 121 | 10227 | 34.23 | 25.13 | 30.63 | 34.67 | 25.58 | 28.98 |
| 122 | 10228 | - | 25.57 | - | - | 26.46 | - |
| 123 | 10229 | 38.46 | 24.18 | - | 37.20 | 25.26 | 42.96 |
| 124 | 10230 | 36.48 | 24.04 | 32.94 | 38.02 | 25.29 | 30.69 |
| 125 | 10231 | - | 26.25 | 34.68 | - | 27.79 | 41.45 |
| 126 | 10232 | 34.37 | 23.74 | 31.08 | 33.88 | 25.09 | 29.39 |
| 127 | 10233 | 32.45 | 24.79 | 30.02 | 36.83 | 26.61 | 35.88 |
| 128 | 10234 | 32.73 | 24.80 | 30.77 | 31.98 | 25.01 | 29.18 |
| 129 | 10235 | 36.50 | 24.86 | 32.82 | 35.56 | 25.67 | 32.73 |
| 130 | 10236 | 28.59 | 26.55 | 24.58 | 28.28 | 27.23 | 23.90 |
| 131 | 10237 | 39.00 | 27.23 | 32.47 | 43.47 | 28.86 | 34.24 |
| 132 | 10238 | - | 25.44 | - | - | 26.56 | - |
| 133 | 10239 | 37.90 | 26.50 | 34.29 | 37.08 | 27.51 | 34.17 |
| 134 | 10240 | 33.65 | 28.87 | 30.24 | 34.82 | 29.78 | 29.59 |
| 135 | 10241 | 32.31 | 26.66 | 28.81 | 32.54 | 27.34 | 28.35 |
| 136 | 10242 | 34.05 | 24.80 | 30.78 | 33.72 | 24.94 | 29.68 |
| 137 | 10243 | 31.62 | 24.18 | 29.27 | 31.21 | 25.07 | 28.40 |
| 138 | 10244 | 34.76 | 26.17 | 32.52 | 35.32 | 26.96 | 31.36 |
| 139 | 10245 | 36.50 | 23.24 | 34.18 | 38.34 | 24.33 | 33.44 |
| 140 | 10246 | 35.69 | 26.44 | 33.00 | 37.69 | 27.91 | 32.15 |
| 141 | 10247 | 34.23 | 24.88 | 31.19 | 34.38 | 25.83 | 30.33 |
| 142 | 10248 | 36.44 | 23.46 | 35.48 | 36.37 | 24.02 | 34.72 |
| 143 | 10249 | 31.94 | 26.76 | 29.79 | 31.69 | 27.15 | 29.02 |
| 144 | 10253 | 30.72 | 24.14 | 28.76 | 30.36 | 24.36 | 27.87 |
| 145 | 10256 | 34.76 | 23.90 | 32.57 | 36.22 | 24.96 | 32.12 |
| 146 | 10257 | - | 23.29 | 35.87 | - | 24.55 | 35.75 |
| 147 | 10258 | 38.14 | 24.10 | 36.58 | - | 25.24 | - |
| 148 | 10259 | 28.89 | 27.75 | 26.58 | 28.73 | 28.10 | 25.91 |
| 149 | 10260 | 28.65 | 27.69 | 26.72 | 27.78 | 28.18 | 25.99 |
| 150 | 10261 | 24.81 | 26.32 | 21.83 | 23.96 | 27.73 | 21.20 |
| 151 | 10262 | 32.43 | 26.89 | 30.19 | 31.50 | 27.47 | 29.70 |
| 152 | 10263 | 32.10 | 23.62 | 30.42 | 31.61 | 24.25 | 29.22 |
| 153 | 10264 | 28.57 | 25.79 | 26.62 | 28.34 | 26.15 | 26.22 |
| 154 | 10265 | 37.11 | 27.20 | 38.09 | 38.04 | 27.85 | 37.11 |
| 155 | 10266 | 33.73 | 24.90 | 32.02 | 34.48 | 25.97 | 30.87 |
| 156 | 10267 | 29.64 | 26.25 | 27.15 | 29.79 | 27.14 | 26.57 |
| 157 | 10268 | 30.53 | 25.83 | 28.63 | 30.60 | 26.79 | 28.16 |
| 158 | 10269 | 34.99 | 25.30 | 32.56 | 35.56 | 26.83 | 32.45 |
| 159 | 10270 | 32.86 | 29.27 | 31.70 | 33.07 | 29.82 | 31.59 |
| 160 | 10271 | 36.86 | 29.71 | 34.73 | 39.44 | 30.55 | 35.07 |
| 161 | 10272 | 35.79 | 26.48 | 35.49 | 36.37 | 27.32 | 34.85 |
| 162 | 10273 | 37.09 | 29.47 | 35.02 | 38.20 | 30.35 | 37.22 |
| 163 | 10274 | 35.93 | 25.42 | 33.82 | 36.80 | 26.89 | 33.20 |
| 164 | 10275 | 35.74 | 25.79 | 34.01 | 36.76 | 26.95 | 34.42 |
| 165 | 10276 | 30.47 | 23.93 | 27.68 | 30.36 | 25.33 | 27.04 |
| 166 | 10277 | 36.02 | 25.98 | 33.56 | 34.74 | 26.98 | 33.15 |
| 167 | 10278 | 33.51 | 25.40 | 31.82 | 33.24 | 26.04 | 30.89 |
| 168 | 10279 | 30.11 | 26.79 | 27.73 | 29.11 | 26.51 | 26.89 |
| 169 | 10280 | 32.88 | 24.61 | 31.08 | 33.28 | 25.29 | 30.16 |
| 170 | 10281 | 31.01 | 26.56 | 28.47 | 30.93 | 23.28 | 27.64 |
| 171 | 10282 | 35.32 | 25.17 | 33.49 | 38.03 | 26.49 | 33.60 |
| 172 | 10283 | 28.83 | 25.73 | 26.29 | 28.55 | 26.84 | 25.81 |
| 173 | 10284 | 34.05 | 27.85 | 31.39 | 33.86 | 29.01 | 30.44 |
| 174 | 10285 | 32.01 | 26.64 | 29.49 | 32.02 | 27.71 | 28.83 |
| 175 | 10286 | 32.30 | 23.77 | 30.08 | 32.31 | 24.52 | 29.39 |
| 176 | 10287 | 31.87 | 28.56 | 29.01 | 31.13 | 28.33 | 27.97 |
| 177 | 10288 | 35.54 | 24.24 | 36.14 | 35.15 | 24.97 | 34.90 |
| 178 | 10289 | 27.58 | 24.93 | 27.68 | 27.44 | 25.18 | 26.52 |
| 179 | 10290 | 35.33 | 25.25 | 35.45 | 35.49 | 25.98 | 34.97 |
| 180 | 10291 | 31.15 | 26.89 | 31.20 | 30.79 | 27.43 | 30.44 |
| 181 | 10292 | 27.90 | 25.04 | 28.55 | 27.89 | 25.34 | 27.35 |
| 182 | 10293 | 29.75 | 22.79 | 30.64 | 29.50 | 23.45 | 28.94 |
| 183 | 10294 | 29.99 | 25.68 | 30.87 | 30.05 | 26.35 | 30.25 |
| 184 | 10295 | 36.37 | 25.93 | - | - | 26.24 | 36.96 |
| 185 | 10296 | 30.66 | 26.30 | 31.69 | 30.87 | 26.60 | 30.44 |
| 186 | 10297 | 35.47 | 22.41 | 38.43 | - | 23.12 | 35.89 |
| 187 | 10298 | 36.80 | 24.90 | 37.04 | 38.27 | 25.57 | - |
| 188 | 10299 | 33.94 | 23.68 | 33.92 | 34.60 | 24.51 | 33.49 |
| 189 | 10300 | 30.70 | 25.96 | 30.55 | 30.45 | 26.39 | 29.32 |
| 190 | 10301 | 35.79 | 26.07 | 36.31 | 36.67 | 26.74 | 35.94 |
| 191 | 10302 | 31.50 | 23.28 | 32.56 | 31.62 | 24.10 | 31.58 |
| 192 | 10303 | 37.20 | 26.57 | 39.01 | 36.58 | 26.80 | 38.03 |
| 193 | 10304 | 27.57 | 24.91 | 28.04 | 27.56 | 25.31 | 26.98 |
| 194 | 10305 | 28.00 | 27.03 | 28.64 | 27.84 | 27.39 | 27.29 |
| 195 | 10306 | 28.56 | 22.28 | 28.74 | 28.40 | 22.95 | 27.55 |
| 196 | 10307 | 35.75 | 21.73 | 37.41 | 36.70 | 22.68 | 35.80 |
| 197 | 10308 | 26.45 | 23.33 | 27.51 | 26.39 | 23.92 | 26.54 |
| 198 | 10309 | 32.60 | 23.68 | 33.62 | 32.43 | 24.42 | 32.40 |
| 199 | 10310 | 30.19 | 24.20 | 30.77 | 30.13 | 24.81 | 29.16 |
| 200 | 10311 | - | 22.40 | - | - | 22.85 | - |
| 201 | 10312 | 33.03 | 23.98 | 34.24 | 33.26 | 24.83 | 33.52 |
| 202 | 10313 | 26.22 | 26.10 | 27.33 | 25.90 | 26.33 | 26.01 |
| 203 | 10314 | 35.62 | 26.23 | 35.62 | 35.83 | 26.82 | 36.10 |
| 204 | 10315 | 29.02 | 23.87 | 29.57 | 28.75 | 24.49 | 28.43 |
| 205 | 10317 | 33.97 | 25.30 | 35.18 | 35.65 | 25.80 | 34.24 |
| 206 | 10318 | - | 25.60 | 36.46 | 38.11 | 26.12 | 37.06 |
| 207 | 10319 | - | 25.41 | - | - | 26.06 | - |
| 208 | 10320 | 34.78 | 25.67 | 35.13 | 35.68 | 25.83 | 34.88 |

Abbreviations: No., number; Ct, cycle threshold; SARS-CoV-2, severe acute respiratory syndrome coronavirus 2; IC, internal control; -, negative.

**Table S6** Clinical data of IAV specimens

| Serial No. | Specimen | FQ-8B  (Ct values) | | ABI-Q7  (Ct values) | |
| --- | --- | --- | --- | --- | --- |
|  |  | IAV | IC (*rnase p*) | IAV | IC (*rnase p*) |
| 1 | 20001 | 32.59 | 29.76 | 31.54 | 29.97 |
| 2 | 20002 | 37.08 | 31.62 | 37.70 | 30.86 |
| 3 | 20003 | 35.32 | 32.21 | - | 32.74 |
| 4 | 20004 | 35.06 | 31.03 | 35.40 | 31.82 |
| 5 | 20005 | - | 30.30 | - | 30.68 |
| 6 | 20006 | - | 32.57 | - | 33.58 |
| 7 | 20007 | - | 30.48 | - | 30.30 |
| 8 | 20008 | - | 28.98 | - | 29.65 |
| 9 | 20009 | 31.01 | 30.04 | 29.82 | 30.39 |
| 10 | 20010 | - | 30.52 | - | 31.17 |
| 11 | 20011 | 30.76 | 29.23 | 29.47 | 30.07 |
| 12 | 20012 | 29.80 | 32.56 | 28.25 | 33.29 |
| 13 | 20013 | 34.99 | 29.17 | 36.91 | 29.01 |
| 14 | 20014 | 38.74 | 29.60 | 36.91 | 29.91 |
| 15 | 20015 | 30.56 | 31.70 | 29.36 | 32.01 |
| 16 | 20016 | 36.54 | 28.91 | 35.78 | 29.24 |
| 17 | 20017 | 35.45 | 29.74 | 33.98 | 29.53 |
| 18 | 20018 | 33.59 | 29.72 | 33.99 | 30.05 |
| 19 | 20019 | - | 28.91 | - | 29.10 |
| 20 | 20020 | 35.57 | 31.83 | 35.69 | 31.98 |
| 21 | 20021 | 33.16 | 32.12 | 32.98 | 32.60 |
| 22 | 20022 | 34.11 | 30.59 | 34.30 | 30.80 |
| 23 | 20023 | 36.21 | 32.64 | 36.35 | 33.07 |
| 24 | 20024 | 32.20 | 30.73 | 31.63 | 30.87 |
| 25 | 20025 | 26.69 | 28.69 | 25.59 | 29.28 |
| 26 | 20026 | - | 30.29 | - | 30.76 |
| 27 | 20027 | - | 28.77 | - | 29.23 |
| 28 | 20028 | - | 30.58 | - | 30.90 |
| 29 | 20029 | 28.55 | 31.05 | 27.24 | 31.36 |
| 30 | 20030 | 28.80 | 29.89 | 27.87 | 30.33 |
| 31 | 20031 | 30.48 | 28.95 | 29.50 | 29.12 |
| 32 | 20032 | 33.21 | 33.74 | 32.41 | 35.63 |
| 33 | 20033 | - | 34.75 | - | 34.62 |
| 34 | 20034 | 36.20 | 34.50 | - | 34.88 |
| 35 | 20035 | 31.13 | 34.38 | 29.81 | 34.57 |
| 36 | 20036 | 28.48 | 31.89 | 27.42 | 31.81 |
| 37 | 20037 | 33.66 | 30.17 | 34.12 | 30.48 |
| 38 | 20038 | 36.08 | 34.22 | 36.90 | 34.79 |
| 39 | 20039 | 35.34 | 35.47 | 35.95 | 36.54 |
| 40 | 20040 | 33.42 | 32.18 | 33.51 | 32.10 |
| 41 | 20041 | 31.06 | 33.85 | 29.66 | 34.95 |
| 42 | 20042 | 30.71 | 28.65 | 29.42 | 28.85 |
| 43 | 20043 | 33.26 | 29.99 | 34.24 | 29.95 |
| 44 | 20044 | 34.15 | 34.18 | 34.84 | 34.53 |
| 45 | 20045 | 36.11 | 31.11 | 34.41 | 31.52 |
| 46 | 20046 | - | 32.67 | - | 32.19 |
| 47 | 20047 | 33.00 | 30.58 | 31.81 | 30.31 |
| 48 | 20048 | - | 28.97 | - | 28.88 |
| 49 | 20049 | - | 30.77 | - | 30.35 |
| 50 | 20050 | 32.89 | 33.88 | 32.27 | 34.67 |
| 51 | 20051 | 36.56 | 36.03 | 37.04 | 37.55 |
| 52 | 20052 | - | 28.52 | - | 29.28 |
| 53 | 20053 | 32.55 | 32.14 | 32.97 | 33.06 |
| 54 | 20054 | 33.75 | 31.58 | 34.07 | 32.45 |
| 55 | 20055 | 33.86 | 34.22 | 36.58 | 36.04 |
| 56 | 20056 | - | 33.19 | - | 33.94 |
| 57 | 20057 | 36.20 | 35.68 | 36.89 | 35.37 |
| 58 | 20058 | 32.87 | 33.83 | 32.94 | 35.20 |
| 59 | 20059 | 31.91 | 30.97 | 30.95 | 31.42 |
| 60 | 20060 | 31.75 | 30.93 | 31.18 | 31.76 |
| 61 | 20061 | 27.43 | 31.89 | 25.88 | 32.42 |
| 62 | 20062 | 29.15 | 30.49 | 27.88 | 30.96 |
| 63 | 20063 | - | 29.94 | - | 30.09 |
| 64 | 20064 | 23.68 | 31.93 | 22.09 | 32.43 |
| 65 | 20065 | - | 31.67 | - | 31.90 |
| 66 | 20066 | 34.53 | 31.51 | 36.87 | 31.91 |
| 67 | 20067 | 36.16 | 29.89 | 36.92 | 30.00 |
| 68 | 20068 | 29.92 | 29.71 | 28.68 | 30.15 |
| 69 | 20069 | - | 30.47 | - | 30.90 |
| 70 | 20070 | 27.06 | 28.22 | 25.74 | 28.87 |
| 71 | 20071 | 30.50 | 28.56 | 29.77 | 28.90 |
| 72 | 20072 | - | 31.19 | - | 31.39 |
| 73 | 20073 | 31.76 | 29.79 | 30.72 | 29.68 |
| 74 | 20074 | 29.55 | 28.60 | 27.97 | 28.43 |
| 75 | 20075 | 34.80 | 33.52 | 34.45 | 34.62 |
| 76 | 20076 | - | 28.47 | - | 28.99 |
| 77 | 20077 | 32.16 | 31.73 | 31.29 | 32.62 |
| 78 | 20078 | 34.66 | 32.05 | 35.31 | 32.21 |
| 79 | 20079 | 32.55 | 32.47 | 30.59 | 33.26 |
| 80 | 20080 | 32.59 | 29.49 | 31.29 | 29.55 |
| 81 | 20081 | - | 27.38 | - | 27.91 |
| 82 | 20082 | 34.79 | 29.77 | 34.51 | 30.18 |
| 83 | 20083 | 35.96 | 30.80 | 35.28 | 30.75 |
| 84 | 20084 | 36.37 | 31.63 | 37.00 | 30.62 |
| 85 | 20085 | 22.86 | 30.00 | 25.51 | 29.61 |
| 86 | 20086 | 22.21 | 29.88 | 24.66 | 29.36 |
| 87 | 20087 | 27.95 | 27.87 | 27.11 | 27.27 |
| 88 | 20088 | - | 29.35 | - | 28.67 |
| 89 | 20089 | - | 30.80 | - | 30.26 |
| 90 | 20090 | - | 28.76 | - | 31.36 |
| 91 | 20091 | 31.58 | 30.53 | 30.80 | 30.64 |
| 92 | 20092 | 30.83 | 30.86 | 30.20 | 32.84 |
| 93 | 20093 | - | 24.43 | - | 27.76 |
| 94 | 20094 | - | 25.70 | - | 28.58 |
| 95 | 20095 | 32.78 | 31.60 | 32.74 | 31.28 |
| 96 | 20096 | 28.40 | 30.97 | 27.31 | 30.72 |
| 97 | 20097 | 33.66 | 25.42 | 33.80 | 28.71 |
| 98 | 20098 | 28.90 | 26.72 | 28.27 | 28.66 |
| 99 | 20099 | 35.57 | 30.57 | 35.23 | 30.16 |
| 100 | 20100 | 24.11 | 30.97 | 23.28 | 30.79 |
| 101 | 20101 | - | 30.92 | - | 30.41 |
| 102 | 20102 | - | 31.69 | - | 31.60 |
| 103 | 20103 | 32.89 | 27.12 | 32.65 | 30.04 |
| 104 | 20104 | - | 30.09 | - | 29.54 |
| 105 | 20105 | - | 30.01 | - | 29.65 |
| 106 | 20106 | - | 30.09 | - | 30.07 |
| 107 | 20107 | 34.60 | 29.57 | 34.79 | 32.07 |
| 108 | 20108 | 23.82 | 33.01 | 26.36 | 34.71 |
| 109 | 20109 | 27.56 | 28.62 | 26.49 | 28.96 |
| 110 | 20110 | - | 32.60 | - | 33.83 |
| 111 | 20111 | 33.03 | 29.60 | 33.00 | 32.02 |
| 112 | 20112 | - | 31.58 | 36.97 | 31.00 |
| 113 | 20113 | 35.11 | 34.01 | 35.94 | 32.54 |
| 114 | 20114 | 32.98 | 33.13 | 32.09 | 33.51 |
| 115 | 20115 | 33.48 | 31.96 | - | 34.96 |
| 116 | 20116 | - | 31.79 | - | 32.17 |
| 117 | 20117 | 24.19 | 33.94 | 25.89 | 33.87 |
| 118 | 20118 | 34.36 | 33.77 | 33.72 | 33.89 |
| 119 | 20119 | 33.67 | 28.69 | 33.89 | 30.51 |
| 120 | 20120 | 36.57 | 33.77 | 34.02 | 34.19 |
| 121 | 20121 | 29.85 | 31.37 | 29.10 | 31.14 |
| 122 | 20122 | 32.46 | 27.67 | 31.28 | 27.89 |
| 123 | 20123 | 31.90 | 28.87 | 31.13 | 30.70 |
| 124 | 20124 | 32.10 | 27.28 | 31.12 | 29.90 |
| 125 | 20125 | - | 30.04 | - | 29.67 |
| 126 | 20126 | 35.38 | 31.49 | 37.12 | 30.53 |
| 127 | 20127 | 35.53 | 29.68 | 36.15 | 30.72 |
| 128 | 20128 | 32.87 | 36.97 | 31.68 | 37.14 |
| 129 | 20129 | 30.11 | 31.18 | 29.25 | 31.67 |
| 130 | 20130 | 28.45 | 31.55 | 27.29 | 31.57 |
| 131 | 20131 | 35.81 | 31.44 | 36.67 | 32.63 |
| 132 | 20132 | 36.85 | 34.40 | 37.13 | 29.90 |
| 133 | 20133 | 27.28 | 31.64 | 26.41 | 30.22 |
| 134 | 20134 | 33.99 | 33.97 | 34.85 | 34.00 |
| 135 | 20135 | - | 27.54 | 37.11 | 30.34 |
| 136 | 20136 | - | 31.85 | - | 32.40 |
| 137 | 20137 | - | 29.20 | - | 30.41 |
| 138 | 20138 | 36.21 | 29.49 | 36.22 | 31.87 |
| 139 | 20139 | 30.02 | 30.65 | 29.41 | 32.90 |
| 140 | 20140 | 30.74 | 31.29 | 30.17 | 33.20 |
| 141 | 20141 | 35.56 | 32.78 | 36.45 | 33.25 |
| 142 | 20142 | 29.13 | 31.64 | 28.44 | 33.67 |
| 143 | 20143 | 33.13 | 31.89 | 32.24 | 32.94 |
| 144 | 20144 | 35.98 | 31.79 | - | 32.12 |
| 145 | 20145 | 34.12 | 29.65 | 34.00 | 30.21 |
| 146 | 20146 | 32.25 | 31.09 | 32.30 | 31.78 |
| 147 | 20147 | - | 29.15 | - | 30.37 |
| 148 | 20148 | 36.85 | 30.06 | 35.45 | 31.14 |
| 149 | 20149 | 28.82 | 30.79 | 28.12 | 31.75 |
| 150 | 20150 | - | 30.99 | - | 32.59 |
| 151 | 20151 | 33.24 | 30.78 | 33.55 | 31.38 |
| 152 | 20152 | - | 32.51 | - | 33.69 |
| 153 | 20153 | 28.55 | 29.89 | 27.59 | 30.48 |
| 154 | 20154 | 30.82 | 30.24 | 30.09 | 31.00 |
| 155 | 20155 | 26.47 | 31.25 | 25.26 | 32.26 |
| 156 | 20156 | 33.13 | 31.53 | 33.48 | 33.17 |
| 157 | 20157 | - | 31.40 | - | 32.97 |
| 158 | 20158 | 36.47 | 27.21 | - | 33.04 |
| 159 | 20159 | 34.93 | 31.55 | 38.22 | 28.90 |
| 160 | 20160 | 30.81 | 30.80 | 29.98 | 30.97 |
| 161 | 20161 | - | 28.62 | - | 32.39 |
| 162 | 20162 | 32.00 | 31.59 | 32.19 | 32.72 |
| 163 | 20163 | 33.24 | 32.42 | 32.44 | 34.38 |
| 164 | 20164 | - | 34.03 | - | 36.99 |
| 165 | 20165 | - | 30.18 | - | 32.31 |
| 166 | 20166 | 33.75 | 33.81 | 34.29 | 34.66 |
| 167 | 20167 | 31.45 | 30.97 | 30.63 | 31.70 |
| 168 | 20168 | 27.72 | 30.64 | 26.61 | 33.57 |
| 169 | 20169 | 30.92 | 30.86 | 29.69 | 31.60 |
| 170 | 20170 | 31.46 | 29.23 | 30.05 | 30.62 |
| 171 | 20171 | 28.58 | 29.96 | 26.83 | 29.49 |
| 172 | 20172 | 31.46 | 34.32 | 30.82 | 29.88 |
| 173 | 20173 | 33.57 | 32.67 | 34.43 | 32.59 |
| 174 | 20174 | 31.64 | 31.15 | 30.84 | 31.13 |
| 175 | 20175 | 32.05 | 30.88 | 31.69 | 31.32 |
| 176 | 20176 | 26.57 | 31.46 | 25.16 | 32.01 |
| 177 | 20177 | 34.42 | 32.06 | 33.62 | 33.09 |
| 178 | 20178 | 30.48 | 30.92 | 29.42 | 31.51 |
| 179 | 20179 | 33.24 | 31.81 | 32.81 | 32.54 |
| 180 | 20180 | 26.40 | 28.68 | 25.35 | 29.49 |
| 181 | 20181 | 33.14 | 30.43 | 31.91 | 31.08 |
| 182 | 20182 | 35.34 | 32.69 | - | 33.58 |
| 183 | 20183 | 32.46 | 31.15 | 31.84 | 32.29 |
| 184 | 20184 | 34.14 | 30.47 | 35.09 | 31.51 |
| 185 | 20185 | 35.83 | 29.76 | - | 30.46 |
| 186 | 20186 | 32.78 | 31.92 | 32.68 | 32.66 |
| 187 | 20187 | 28.54 | 30.72 | 27.19 | 32.28 |
| 188 | 20188 | 32.86 | 30.97 | 33.08 | 32.68 |
| 189 | 20189 | 35.29 | 33.25 | 36.11 | 34.33 |
| 190 | 20190 | 33.95 | 29.49 | 35.40 | 30.50 |
| 191 | 20191 | 28.06 | 30.89 | 26.92 | 31.69 |
| 192 | 20192 | 27.29 | 29.23 | 25.94 | 30.63 |
| 193 | 20193 | 36.60 | 28.73 | 36.48 | 30.11 |
| 194 | 20194 | 34.17 | 32.07 | - | 33.08 |
| 195 | 20195 | 35.67 | 29.07 | 35.88 | 29.99 |
| 196 | 20196 | 33.03 | 32.15 | 32.64 | 33.04 |
| 197 | 20197 | 32.08 | 29.41 | 31.45 | 29.15 |
| 198 | 20198 | - | 32.70 | - | 33.08 |
| 199 | 20199 | 31.84 | 31.11 | 30.71 | 31.99 |
| 200 | 20200 | - | 32.93 | - | 34.06 |
| 201 | 20201 | 31.88 | 32.27 | 31.88 | 33.17 |
| 202 | 20202 | - | 30.55 | - | 31.89 |
| 203 | 20203 | 37.37 | 29.51 | 36.95 | 30.38 |
| 204 | 20204 | 33.04 | 29.25 | 33.56 | 30.36 |
| 205 | 20205 | 28.07 | 30.21 | 26.93 | 31.24 |
| 206 | 20206 | 23.99 | 26.99 | 23.21 | 28.30 |
| 207 | 20207 | - | 32.61 | - | 34.11 |
| 208 | 20208 | 35.21 | 30.58 | 35.94 | 31.99 |
| 209 | 20209 | - | 31.16 | - | 32.13 |
| 210 | 20210 | - | 32.00 | - | 32.61 |
| 211 | 20211 | - | 30.22 | - | 31.14 |
| 212 | 20212 | - | 30.71 | - | 31.25 |
| 213 | 20213 | - | 32.06 | - | 32.83 |
| 214 | 20214 | 31.92 | 30.07 | 31.57 | 30.79 |
| 215 | 20215 | 33.56 | 31.35 | 33.31 | 32.35 |
| 216 | 20216 | 26.49 | 31.42 | 25.33 | 32.77 |

Abbreviations: No., number; Ct, cycle threshold; IAV, influenza A virus; IC, internal control; -, negative.

**Table S7** Clinical data of 1227 patient specimens with fever and cold symptoms

| **Serial No.** | **Date** | **Gender** | **Age** | **FQ-8B Rapid test**  **(Ct values)** | | | | | | | | | | | | | | | | **Standard qRT-PCR test**  **(Ct values)** | | | |
| --- | --- | --- | --- | --- | --- | --- | --- | --- | --- | --- | --- | --- | --- | --- | --- | --- | --- | --- | --- | --- | --- | --- | --- |
|  |  |  |  | IBV | ADV | IAV | IC (rnase p) | SARS-CoV-2 | HSV-1 | EV | HMPV | HRV | BP | MP | CP | RSV | HBoV | HPIV | HCoV | SARS-CoV-2 | IAV | IBV | IC (rnase p) |
| 1 | 23.08.21 | Female | 52 | - | - | - | 23.05 | - | - | - | - | - | - | - | - | - | - | - | - | - | - | - | 23.8 |
| 2 | 23.08.21 | Male | 53 | - | - | - | 29.53 | - | - | - | - | - | - | - | - | 26.49 | - | - | - | - | - | - | 29.88 |
| 3 | 23.08.21 | Male | 49 | - | - | - | 29.49 | - | - | - | - | 28.19 | - | - | - | - | - | - | - | - | - | - | 30.1 |
| 4 | 23.08.21 | Male | 43 | - | - | - | 28.16 | 25.86 | - | - | - | - | - | - | - | - | - | - | - | 25.07 | - | - | 28.48 |
| 5 | 23.08.21 | Female | 30 | 29.86 | - | - | 29.47 | - | - | - | - | - | - | - | - | - | - | - | - | - | - | 34.51 | 31.9 |
| 6 | 23.08.21 | Male | 54 | - | - | - | 27.18 | 23.8 | - | - | - | - | - | - | - | - | - | - | - | 23.54 | - | - | 27.5 |
| 7 | 23.08.22 | Female | 35 | - | - | - | 23.09 | 26.4 | - | - | - | - | - | - | - | - | - | - | - | 28.06 | - | - | 24.58 |
| 8 | 23.08.22 | Female | 64 | - | - | - | 25.13 | 30 | - | - | - | - | - | - | - | - | - | - | - | 30.28 | - | - | 25.29 |
| 9 | 23.08.22 | Female | 58 | - | - | - | 30.73 | - | - | - | - | - | - | - | - | - | - | - | - | - | - | - | 31.23 |
| 10 | 23.08.22 | Female | 15 | - | - | 28.77 | 30.8 | - | - | - | - | - | - | - | - | - | - | - | - | - | 33.24 | - | 32.69 |
| 11 | 23.08.22 | Female | 31 | - | - | - | 29.85 | - | - | - | - | - | - | - | - | - | - | - | - | - | - | - | 29 |
| 12 | 23.08.22 | Male | 38 | - | - | - | 29.44 | - | - | - | - | - | - | - | - | - | - | - | - | - | - | - | 30.18 |
| 13 | 23.08.22 | Female | 7 | - | - | - | 31.17 | - | - | - | - | - | - | 28.58 | - | - | - | - | - | - | - | - | 31.85 |
| 14 | 23.08.22 | Female | 35 | - | - | - | 28.08 | 28.55 | - | - | - | - | - | - | - | - | - | - | - | 32.86 | - | - | 31.63 |
| 15 | 23.08.23 | Female | 87 | - | - | - | 31.34 | - | - | - | - | - | - | - | - | - | - | 34.2 | - | - | - | - | 31.34 |
| 16 | 23.08.23 | Male | 12 | - | - | - | 28.8 | 28.39 | - | - | - | - | - | - | - | - | - | - | - | 27.95 | - | - | 29.43 |
| 17 | 23.08.23 | Female | 8 | - | - | - | 30.3 | - | - | - | - | - | - | - | - | - | - | - | - | - | - | - | 30.17 |
| 18 | 23.08.23 | Male | 27 | - | - | - | 29.87 | 28.92 | - | - | - | - | - | - | - | - | - | - | - | 31.75 | - | - | 31.06 |
| 19 | 23.08.23 | Male | 21 | - | - | - | 31.68 | - | 28.24 | - | - | - | - | - | - | - | - | - | - | - | - | - | 31.96 |
| 20 | 23.08.23 | Female | 58 | - | - | - | 27.54 | - | - | - | - | - | - | - | - | - | - | - | - | - | - | - | 27.82 |
| 21 | 23.08.23 | Male | 33 | - | - | - | 29.03 | - | - | - | - | - | - | - | - | - | - | - | - | - | - | - | 29.79 |
| 22 | 23.08.23 | Female | 46 | - | - | - | 24.99 | - | - | - | - | - | - | - | - | - | - | - | - | - | - | - | 24.8 |
| 23 | 23.08.23 | Male | 51 | - | - | - | 29.26 | - | - | - | - | - | - | - | - | - | - | - | - | - | - | - | 28.91 |
| 24 | 23.08.24 | Female | 55 | - | - | - | 26.15 | - | - | - | - | - | - | - | - | - | - | - | - | - | - | - | 26.79 |
| 25 | 23.08.24 | Female | 52 | - | - | - | 24.98 | - | - | - | - | - | - | - | - | - | - | - | - | - | - | - | 25.61 |
| 26 | 23.08.24 | Male | 31 | - | - | - | 27.66 | - | - | - | - | - | - | - | - | - | - | - | - | - | - | - | 27.29 |
| 27 | 23.08.24 | Male | 19 | - | - | - | 30.92 | - | - | - | - | - | - | - | - | - | - | - | - | - | - | - | 30.6 |
| 28 | 23.08.24 | Female | 49 | - | - | - | 29.7 | - | - | - | - | - | - | - | - | - | - | - | - | - | - | - | 31.41 |
| 29 | 23.08.24 | Male | 49 | - | - | - | 29.77 | - | - | - | - | - | - | 30.9 | - | - | - | - | - | - | - | - | 31.09 |
| 30 | 23.08.24 | Female | 56 | - | - | - | 29.21 | - | - | - | - | - | - | - | - | 26.96 | - | - | - | - | - | - | 31.51 |
| 31 | 23.08.25 | Female | 46 | - | - | - | 31.42 | 27.77 | - | - | - | - | - | - | - | - | - | - | - | 30.6 | - | - | 31.93 |
| 32 | 23.08.25 | Male | 71 | - | - | - | 24.57 | - | - | - | - | - | - | - | - | - | - | - | - | - | - | - | 25.36 |
| 33 | 23.08.25 | Female | 75 | - | - | - | 27.24 | - | - | - | - | - | - | - | - | - | - | - | - | - | - | - | 28.06 |
| 34 | 23.08.25 | Female | 30 | - | - | - | 28.7 | - | - | - | - | 26.73 | - | - | - | - | - | - | - | - | - | - | 27.8 |
| 35 | 23.08.25 | Female | 19 | - | - | - | 25.09 | - | 25.05 | - | - | - | - | - | - | - | - | - | - | - | - | - | 26.27 |
| 36 | 23.08.25 | Female | 43 | - | - | - | 32.82 | 30.32 | - | - | - | - | - | - | - | - | - | - | - | 33.96 | - | - | 32.3 |
| 37 | 23.08.25 | Male | 52 | - | - | - | 31.68 | - | - | - | - | - | - | - | - | - | - | - | - | - | - | - | 33.77 |
| 38 | 23.08.25 | Female | 51 | - | - | - | 28.96 | - | - | - | - | - | - | - | - | - | - | - | - | - | - | - | 29.39 |
| 39 | 23.08.25 | Female | 25 | - | - | - | 29.87 | 25.36 | - | - | - | - | - | - | - | - | - | - | - | 26.77 | - | - | 31.29 |
| 40 | 23.08.28 | Female | 8 | 29.47 | - | - | 29.25 | - | - | - | - | - | - | - | - | - | - | - | - | - | - | 33.51 | 30.94 |
| 41 | 23.08.28 | Male | 21 | - | - | - | 29.75 | - | - | - | - | - | - | - | - | - | - | - | - | - | - | - | 29.43 |
| 42 | 23.08.29 | Female | 61 | - | - | - | 27.96 | - | - | - | - | - | - | - | - | - | - | - | - | - | - | - | 29.08 |
| 43 | 23.08.29 | Male | 74 | - | - | - | 28.18 | - | - | - | - | - | - | - | - | - | - | - | - | - | - | - | 29.19 |
| 44 | 23.08.29 | Female | 49 | - | - | - | 27.43 | - | - | - | - | - | - | - | - | - | - | 28.81 | - | - | - | - | 27.5 |
| 45 | 23.08.29 | Female | 15 | - | - | - | 32.56 | - | - | - | - | - | - | - | - | - | - | - | - | - | - | - | 34.62 |
| 46 | 23.08.30 | Female | 17 | - | - | - | 31.69 | - | - | - | - | - | - | - | - | - | - | - | - | - | - | - | 33.62 |
| 47 | 23.08.30 | Female | 4 | - | - | - | 29.07 | - | - | - | - | - | - | - | - | - | - | - | - | - | - | - | 29.73 |
| 48 | 23.08.30 | Female | 23 | - | - | - | 31.57 | - | - | - | - | - | - | - | - | - | - | - | - | - | - | - | 32.99 |
| 49 | 23.08.30 | Male | 3 | - | - | - | 32.52 | - | - | - | 29.62 | - | - | - | - | 37 | - | - | - | - | - | - | 34.34 |
| 50 | 23.08.30 | Male | 49 | - | - | - | 25.49 | - | - | - | - | - | - | - | - | - | - | - | - | - | - | - | 26.16 |
| 51 | 23.08.30 | Male | 23 | - | - | - | 27.43 | - | - | - | - | - | - | - | - | - | - | - | - | - | - | - | 27.28 |
| 52 | 23.08.30 | Male | 15 | - | - | - | 32.85 | - | - | - | - | - | - | - | - | - | - | - | - | - | - | - | 31.21 |
| 53 | 23.08.30 | Male | 8 | - | - | - | 33.42 | - | - | - | - | - | - | - | - | - | - | - | - | - | - | - | 31.95 |
| 54 | 23.08.30 | Female | 15 | - | - | - | 32.91 | 30.73 | - | - | - | - | - | - | - | - | - | - | - | 30.51 | - | - | 31.71 |
| 55 | 23.08.30 | Female | 72 | - | - | - | 26.15 | - | - | - | - | - | - | - | - | - | - | - | - | - | - | - | 25.19 |
| 56 | 23.08.31 | Female | 34 | - | - | - | 30.72 | - | - | - | - | - | - | - | - | - | - | - | - | - | - | - | 32.02 |
| 57 | 23.08.31 | Male | 34 | - | - | - | 31.58 | 32.55 | - | - | - | - | - | - | - | - | - | - | - | 31.75 | - | - | 30.35 |
| 58 | 23.08.31 | Female | 40 | - | - | - | 27.44 | - | - | - | - | - | - | - | - | - | - | - | - | - | - | - | 27.26 |
| 59 | 23.08.31 | Female | 37 | - | - | - | 30.85 | 32.24 | - | - | - | - | - | - | - | - | - | - | - | 31.89 | - | - | 31 |
| 60 | 23.08.31 | Female | 46 | - | - | - | 25.68 | - | - | - | - | - | - | - | - | - | - | - | - | - | - | - | 26.26 |
| 61 | 23.08.31 | Male | 26 | - | - | - | 27.65 | - | - | 29.62 | - | 27.21 | - | - | - | - | - | - | - | - | - | - | 27.02 |
| 62 | 23.09.04 | Female | 33 | - | - | - | 29.6 | - | - | - | - | - | - | - | - | - | - | - | - | - | - | - | 29.66 |
| 63 | 23.09.04 | Male | 7 | - | - | 35.01 | 33.4 | - | - | - | - | - | - | - | - | - | - | - | - | - | 36.78 | - | 32.08 |
| 64 | 23.09.04 | Female | 30 | - | - | - | 32.02 | 27.32 | - | - | - | - | - | - | - | - | - | - | - | 25.73 | - | - | 31.01 |
| 65 | 23.09.04 | Female | 20 | - | - | - | 31.18 | 31.62 | - | - | - | - | - | - | - | - | - | - | - | 29.68 | - | - | 29.95 |
| 66 | 23.09.04 | Male | 25 | - | - | - | 29.61 | - | - | - | - | - | - | - | - | - | - | - | - | - | - | - | 29.14 |
| 67 | 23.09.04 | Male | 23 | - | - | - | 28.93 | - | - | - | - | - | - | - | - | - | - | - | - | - | - | - | 28.06 |
| 68 | 23.09.04 | Female | 21 | - | - | - | 28.44 | - | - | - | - | - | - | - | - | - | - | - | - | - | - | - | 28.63 |
| 69 | 23.09.04 | Female | 58 | - | - | - | 28.63 | 28.03 | - | - | - | - | - | - | - | - | - | - | - | 25.45 | - | - | 27.95 |
| 70 | 23.09.05 | Male | 24 | - | - | - | 30.75 | - | - | - | - | - | - | - | - | - | - | - | - | - | - | - | 31.88 |
| 71 | 23.09.05 | Female | 21 | - | - | - | 31.8 | - | - | - | - | - | - | - | - | - | - | - | - | - | - | - | 30.12 |
| 72 | 23.09.05 | Female | 41 | - | - | - | 26.17 | - | - | - | - | - | - | - | - | - | - | - | - | - | - | - | 26.72 |
| 73 | 23.09.05 | Male | 33 | - | - | 24.21 | 26.88 | - | - | - | - | - | - | - | - | - | - | - | - | - | 35.21 | - | 26.31 |
| 74 | 23.09.05 | Male | 27 | - | - | - | 32.49 | - | - | - | - | - | - | - | - | - | - | - | - | - | - | - | 32.4 |
| 75 | 23.09.05 | Female | 25 | - | - | - | 27.8 | - | - | - | - | - | - | - | - | - | - | - | - | - | - | - | 27.08 |
| 76 | 23.09.05 | Female | 40 | - | - | - | 26.65 | - | - | - | - | - | - | - | - | - | - | - | - | - | - | - | 26.61 |
| 77 | 23.09.05 | Female | 40 | - | - | - | 30.19 | - | - | - | - | - | - | - | - | - | - | - | - | - | - | - | 30.06 |
| 78 | 23.09.05 | Female | 23 | - | - | - | 27 | - | - | - | - | - | - | - | - | - | - | - | - | - | - | - | 27.7 |
| 79 | 23.09.05 | Female | 50 | - | - | - | 28.62 | - | - | - | - | - | - | - | - | - | - | - | - | - | - | - | 27.42 |
| 80 | 23.09.06 | Female | 35 | - | - | - | 31.93 | - | - | - | - | - | - | 33.34 | - | - | - | - | - | - | - | - | 31.61 |
| 81 | 23.09.06 | Female | 40 | - | - | - | 32.08 | - | - | - | - | - | - | - | - | - | - | - | - | - | - | - | 29.98 |
| 82 | 23.09.06 | Female | 49 | - | - | - | 30.1 | 28.5 | - | - | - | - | - | - | - | - | - | - | - | 27.28 | - | - | 29.35 |
| 83 | 23.09.06 | Female | 21 | - | - | - | 31.78 | - | - | - | - | - | - | - | - | - | - | - | - | - | - | - | 30.58 |
| 84 | 23.09.06 | Female | 25 | - | - | - | 28.14 | - | - | - | - | - | - | - | - | - | - | - | - | - | - | - | 27.51 |
| 85 | 23.09.07 | Male | 46 | - | - | - | 33.88 | - | - | - | - | - | - | - | - | - | - | - | - | - | - | - | 33.23 |
| 86 | 23.09.07 | Female | 13 | - | - | - | 31.67 | 31.97 | - | - | - | - | - | - | - | - | - | - | - | 31.02 | - | - | 30.81 |
| 87 | 23.09.07 | Male | 16 | - | - | - | 33.63 | 34.06 | - | - | - | - | - | - | - | - | - | - | - | 32.38 | - | - | 32.69 |
| 88 | 23.09.07 | Male | 17 | - | - | - | 33.69 | - | - | 29.55 | - | 30.17 | - | - | - | - | - | - | - | - | - | - | 33.74 |
| 89 | 23.09.07 | Male | 15 | - | - | - | 30.6 | 31.98 | - | - | - | - | - | - | - | - | - | - | - | 30.59 | - | - | 29.17 |
| 90 | 23.09.07 | Female | 26 | - | - | - | 28.02 | - | - | - | - | - | - | - | - | - | - | - | - | - | - | - | 27.27 |
| 91 | 23.09.07 | Male | 15 | - | - | - | 32.78 | 28.56 | - | - | - | - | - | - | - | - | - | - | - | 26.45 | - | - | 31.92 |
| 92 | 23.09.07 | Male | 21 | - | - | - | 29.14 | - | - | - | - | - | - | - | - | - | - | - | - | - | - | - | 28.64 |
| 93 | 23.09.08 | Male | 35 | - | - | - | 31.73 | 30.09 | - | - | - | - | - | - | - | - | - | - | - | 28.07 | - | - | 31.04 |
| 94 | 23.09.08 | Male | 49 | - | - | - | 30.99 | - | - | - | - | - | - | - | - | - | - | - | - | - | - | - | 30.57 |
| 95 | 23.09.08 | Female | 64 | - | - | - | 28.13 | 27.05 | - | - | - | - | - | - | - | - | - | - | - | 24.73 | - | - | 27.18 |
| 96 | 23.09.08 | Male | 42 | - | - | - | 31.61 | - | - | - | - | - | - | - | - | - | - | - | - | - | - | - | 30.61 |
| 97 | 23.09.08 | Female | 57 | - | - | - | 31.05 | - | - | - | - | - | - | - | - | - | - | - | - | - | - | - | 30.17 |
| 98 | 23.09.08 | Male | 45 | - | - | - | 31.46 | - | - | 26.22 | - | 25.44 | - | - | - | - | - | - | - | - | - | - | 30.81 |
| 99 | 23.09.08 | Female | 61 | - | - | - | 26.03 | - | - | - | - | - | - | - | - | - | - | - | - | - | - | - | 27.59 |
| 100 | 23.09.08 | Male | 15 | - | - | - | 29.11 | - | 26.52 | - | - | - | - | - | - | - | - | - | - | - | - | - | 29.98 |
| 101 | 23.09.11 | Female | 11 | - | - | - | 33.92 | - | - | - | - | - | - | - | - | - | - | - | - | - | - | - | 33.68 |
| 102 | 23.09.11 | Female | 3 | - | - | - | 32.75 | - | - | - | - | - | - | - | - | 31.29 | - | - | - | - | - | - | 32.66 |
| 103 | 23.09.11 | Female | 6 | - | - | - | 32.17 | - | - | - | - | - | - | 30 | - | - | - | - | - | - | - | - | 33.03 |
| 104 | 23.09.11 | Female | 35 | - | - | 30.76 | 31.68 | - | - | - | - | - | - | - | - | - | - | - | - | - | 32.4 | - | 31.92 |
| 105 | 23.09.11 | Male | 21 | - | - | 25.23 | 31.23 | - | - | - | - | - | - | - | - | - | - | - | - | - | 23.97 | - | 31.98 |
| 106 | 23.09.11 | Female | 32 | - | - | - | 32.77 | - | - | - | - | - | - | - | - | - | - | - | - | - | - | - | 33.96 |
| 107 | 23.09.12 | Female | 20 | - | - | - | 31.5 | - | - | - | - | - | - | - | - | - | - | - | - | - | - | - | 32.03 |
| 108 | 23.09.12 | Female | 11 | - | - | - | 30 | 26.97 | - | - | - | - | - | - | - | - | - | - | - | 27.43 | - | - | 30.59 |
| 109 | 23.09.13 | Female | 27 | - | - | - | 31.15 | 29.57 | - | - | - | - | - | - | - | - | - | - | - | 29.1 | - | - | 31.31 |
| 110 | 23.09.13 | Male | 16 | - | - | - | 30.97 | - | - | - | - | - | - | - | - | - | - | - | - | - | - | - | 32.01 |
| 111 | 23.09.13 | Male | 20 | - | - | - | 30.91 | 22.02 | - | - | - | - | - | - | - | - | - | - | - | 20.03 | - | - | 32.38 |
| 112 | 23.09.13 | Female | 42 | - | - | - | 28.29 | 26.9 | - | - | - | - | - | - | - | - | - | - | - | 30.04 | - | - | 28.5 |
| 113 | 23.09.13 | Male | 19 | - | - | - | 31.46 | - | - | - | - | - | - | - | - | - | - | - | - | - | - | - | 33.53 |
| 114 | 23.09.13 | Male | 20 | - | - | - | 30.91 | 28.56 | - | - | - | - | - | - | - | - | - | - | - | 29.26 | - | - | 31.38 |
| 115 | 23.09.13 | Male | 61 | - | - | 30.01 | 30.67 | - | - | - | - | - | - | - | - | - | - | - | - | - | 30.57 | - | 30.93 |
| 116 | 23.09.13 | Female | 48 | - | - | - | 29.97 | - | - | - | - | - | - | 31.98 | - | - | - | - | - | - | - | - | 30.27 |
| 117 | 23.09.14 | Female | 28 | - | - | - | 31.85 | - | - | - | - | - | - | - | - | - | - | - | 33.76 | - | - | - | 32.79 |
| 118 | 23.09.14 | Female | 42 | - | - | - | 32.25 | 27.8 | - | - | - | - | - | 27.91 | - | - | - | - | - | 29.42 | - | - | 33.96 |
| 119 | 23.09.14 | Male | 8 | - | - | - | 32.81 | - | - | - | - | 31.04 | - | - | - | - | - | - | - | - | - | - | 35.05 |
| 120 | 23.09.15 | Male | 14 | - | - | - | 31.49 | - | - | - | - | - | - | - | - | - | - | - | - | - | - | - | 33.24 |
| 121 | 23.09.15 | Male | 29 | - | - | - | 30.23 | - | - | - | - | - | - | - | - | - | - | - | - | - | - | - | 30.31 |
| 122 | 23.09.15 | Male | 16 | - | - | - | 29.64 | 27.73 | - | - | - | - | - | - | - | - | - | - | - | 27.66 | - | - | 29.73 |
| 123 | 23.09.18 | Female | 21 | - | - | - | 30.57 | 28.91 | - | - | - | - | - | - | - | - | - | - | - | 32.57 | - | - | 30.15 |
| 124 | 23.09.18 | Male | 15 | - | - | - | 29.63 | - | - | - | - | - | - | - | - | - | - | - | - | - | - | - | 29.96 |
| 125 | 23.09.19 | Female | 67 | - | - | - | 30.5 | 30.74 | - | 37.84 | - | - | - | - | - | - | - | - | - | 32.39 | - | - | 30.79 |
| 126 | 23.09.20 | Male | 39 | - | - | - | 26.7 | - | - | - | - | - | - | - | - | - | - | - | - | - | - | - | 26.24 |
| 127 | 23.09.21 | Female | 38 | - | - | - | 29.89 | 30.06 | - | - | - | - | - | 27 | - | - | - | - | - | 28.79 | - | - | 30.17 |
| 128 | 23.09.21 | Male | 20 | - | - | - | 29.03 | 26.46 | - | - | - | - | - | - | - | - | - | - | - | 26.71 | - | - | 29.83 |
| 129 | 23.09.21 | Male | 20 | 30.94 | - | - | 30.23 | - | - | - | - | - | - | - | - | - | - | - | - | - | - | 33.8 | 29.84 |
| 130 | 23.09.21 | Female | 18 | 32.62 | - | - | 32.48 | - | - | - | - | - | - | - | - | - | - | - | - | - | - | 36.31 | 31.33 |
| 131 | 23.09.21 | Female | 20 | - | - | 34.72 | 33.08 | - | - | - | - | - | - | - | - | - | - | - | - | - | 35.23 | - | 32 |
| 132 | 23.09.21 | Female | 13 | - | - | - | 31.51 | - | - | - | - | - | - | - | - | - | - | - | - | - | - | - | 29.83 |
| 133 | 23.09.21 | Male | 40 | - | - | - | 26.49 | - | - | - | - | - | - | - | - | - | - | - | - | - | - | - | 25.29 |
| 134 | 23.09.21 | Male | 20 | - | - | 29.82 | 30.67 | - | - | - | - | - | - | - | - | - | - | - | - | - | 30.29 | - | 29.98 |
| 135 | 23.09.21 | Male | 21 | - | - | 32.44 | 34.67 | - | - | - | - | - | - | - | - | - | - | - | 32.73 | - | 31.43 | - | 32.95 |
| 136 | 23.09.21 | Female | 39 | - | - | - | 32.24 | - | - | - | - | - | - | - | - | - | - | - | - | - | - | - | 31.39 |
| 137 | 23.09.22 | Female | 55 | - | - | - | 28.72 | 27.46 | - | - | - | - | - | - | - | - | - | - | - | 25.02 | - | - | 27.46 |
| 138 | 23.09.25 | Male | 18 | - | - | - | 31.05 | - | - | 30.16 | - | 27.12 | - | - | - | - | - | - | - | - | - | - | 31.3 |
| 139 | 23.09.25 | Female | 55 | - | - | - | 27.99 | - | - | - | - | - | - | - | - | - | - | - | - | - | - | - | 27.01 |
| 140 | 23.09.25 | Female | 70 | - | - | - | 27.5 | 27.45 | - | - | - | - | - | - | - | - | - | - | - | 26.25 | - | - | 26.75 |
| 141 | 23.09.25 | Female | 30 | - | - | 28.73 | 28.96 | - | - | - | - | - | - | - | - | - | - | - | - | - | 28.89 | - | 28.68 |
| 142 | 23.09.25 | Female | 25 | - | - | 29.91 | 29.16 | - | - | - | - | - | - | - | - | - | - | - | - | - | 33.76 | - | 29.71 |
| 143 | 23.09.25 | Female | 61 | - | - | - | 29.38 | - | - | - | - | - | - | - | - | - | - | - | - | - | - | - | 28.61 |
| 144 | 23.09.25 | Male | 36 | - | - | 25.43 | 26.05 | - | - | - | - | - | - | - | - | - | - | - | - | - | 25.43 | - | 25.77 |
| 145 | 23.09.25 | Female | 35 | - | - | - | 26.86 | - | - | - | - | - | - | - | - | - | - | - | - | - | - | - | 25.86 |
| 146 | 23.09.25 | Male | 21 | - | - | - | 27.7 | - | - | - | - | - | - | - | - | - | - | - | - | - | - | - | 27.02 |
| 147 | 23.09.25 | Male | 27 | - | - | - | 32.5 | - | - | - | - | - | - | - | - | - | - | - | - | - | - | - | 31.91 |
| 148 | 23.09.25 | Female | 22 | - | - | - | 30.89 | - | - | - | - | - | - | - | - | - | - | - | - | - | - | - | 29.74 |
| 149 | 23.09.25 | Male | 52 | - | - | - | 31.97 | - | - | - | - | - | - | - | - | - | - | - | - | - | - | - | 31.68 |
| 150 | 23.09.25 | Male | 16 | - | - | - | 30.83 | - | - | - | - | - | - | - | - | - | - | - | - | - | - | - | 29.77 |
| 151 | 23.09.25 | Female | 74 | - | - | - | 28.11 | - | - | - | - | - | - | - | - | - | - | - | - | - | - | - | 27.59 |
| 152 | 23.09.26 | Male | 54 | - | - | - | 30.89 | - | - | - | - | - | - | - | - | - | - | - | - | - | - | - | 30.97 |
| 153 | 23.09.26 | Female | 29 | - | - | - | 27.89 | - | - | - | 31 | - | - | - | - | - | - | - | - | - | - | - | 27.82 |
| 154 | 23.09.26 | Male | 75 | - | - | - | 25.57 | 25.51 | - | - | - | - | - | - | - | - | - | - | - | 26.87 | - | - | 26.34 |
| 155 | 23.09.27 | Female | 43 | - | - | - | 27.29 | - | - | - | - | - | - | - | - | - | - | - | - | - | - | - | 27.17 |
| 156 | 23.09.27 | Female | 46 | - | - | - | 26.51 | 25.04 | - | - | - | - | - | - | - | - | - | - | - | 24.28 | - | - | 27.46 |
| 157 | 23.09.27 | Female | 26 | - | - | - | 30.07 | - | - | - | - | - | - | - | - | - | - | - | - | - | - | - | 30.81 |
| 158 | 23.09.27 | Female | 23 | - | - | - | 26.96 | - | - | - | - | - | - | - | - | - | - | - | - | - | - | - | 27.04 |
| 159 | 23.09.27 | Male | 26 | - | - | - | 33.08 | - | - | - | - | - | - | - | - | - | - | - | - | - | - | - | 32.2 |
| 160 | 23.09.27 | Male | 17 | - | - | - | 29.24 | - | - | - | - | 28.26 | - | - | - | - | - | - | - | - | - | - | 29.85 |
| 161 | 23.09.27 | Female | 18 | - | - | 27.89 | 28.65 | - | - | - | - | - | - | - | - | - | - | - | - | - | 29.82 | - | 30.27 |
| 162 | 23.09.27 | Male | 15 | - | - | - | 30 | 28.34 | - | - | - | - | - | - | - | - | - | - | - | 27.32 | - | - | 31.35 |
| 163 | 23.09.27 | Male | 20 | - | - | - | 29.71 | - | - | - | - | 29.63 | - | - | - | - | - | - | - | - | - | - | 29 |
| 164 | 23.09.27 | Male | 10 | - | - | 30.51 | 32.14 | - | - | - | - | - | - | - | - | - | - | - | - | - | 32.23 | - | 33.42 |
| 165 | 23.09.27 | Male | 34 | - | - | - | 26.42 | - | - | - | - | - | - | - | - | - | - | - | - | - | - | - | 27.65 |
| 166 | 23.09.28 | Female | 78 | - | - | - | 27.69 | 27.47 | - | - | - | - | - | - | - | - | - | - | - | 28.6 | - | - | 27.93 |
| 167 | 23.10.07 | Female | 28 | - | - | - | 28.23 | - | - | - | 31.13 | - | - | - | - | - | - | - | - | - | - | - | 27.88 |
| 168 | 23.10.08 | Male | 27 | - | - | 26 | 29.84 | - | - | - | - | - | - | - | - | - | - | - | 31.52 | - | 36.36 | - | 31.43 |
| 169 | 23.10.08 | Male | 17 | - | - | - | 30.13 | - | - | - | - | - | - | - | - | - | - | - | - | - | - | - | 32.15 |
| 170 | 23.10.08 | Female | 37 | - | - | - | 28.14 | - | - | - | - | - | - | - | - | - | - | - | - | - | - | - | 28.65 |
| 171 | 23.10.08 | Female | 42 | - | - | - | 25.14 | - | - | - | - | - | - | - | - | - | - | - | - | - | - | - | 25.11 |
| 172 | 23.10.08 | Female | 35 | - | - | 26.13 | 26.02 | - | - | - | - | - | - | - | - | - | - | - | - | - | 29.3 | - | 27.18 |
| 173 | 23.10.09 | Male | 33 | - | - | - | 23.44 | - | - | - | - | - | - | - | - | - | - | - | - | - | - | - | 25.04 |
| 174 | 23.10.09 | Female | 17 | - | - | - | 25.7 | - | - | - | - | - | - | - | - | - | - | - | - | - | - | - | 26.53 |
| 175 | 23.10.09 | Female | 50 | - | - | - | 29.24 | - | - | - | - | - | - | - | - | - | - | - | 28.06 | - | - | - | 29.06 |
| 176 | 23.10.09 | Female | 62 | - | - | - | 24.52 | 28.64 | - | - | - | - | - | - | - | - | - | - | - | 28.7 | - | - | 25.66 |
| 177 | 23.10.09 | Female | 20 | - | - | - | 28.78 | 27.54 | - | - | - | - | - | - | - | - | - | - | - | 25.95 | - | - | 28.77 |
| 178 | 23.10.10 | Male | 37 | - | - | - | 25.86 | - | - | - | - | - | - | - | - | - | - | - | - | - | - | - | 27.24 |
| 179 | 23.10.10 | Male | 45 | - | - | - | 28.12 | - | - | - | - | - | - | - | - | - | - | - | - | - | - | - | 27.33 |
| 180 | 23.10.10 | Female | 26 | - | - | - | 29.06 | - | - | - | - | - | - | - | - | - | - | - | - | - | - | - | 29.49 |
| 181 | 23.10.10 | Male | 33 | - | - | - | 27.86 | - | - | - | 26.7 | - | - | - | - | - | - | - | - | - | - | - | 27.91 |
| 182 | 23.10.10 | Female | 46 | - | - | - | 28.99 | - | - | - | - | - | - | - | - | - | - | - | - | - | - | - | 29.61 |
| 183 | 23.10.11 | Male | 18 | - | - | 32.85 | 31.82 | - | - | - | - | - | - | - | - | - | - | - | - | - | 33.32 | - | 31.58 |
| 184 | 23.10.11 | Male | 31 | - | - | - | 26.97 | - | - | - | - | - | - | - | - | - | - | - | 27.24 | - | - | - | 27.14 |
| 185 | 23.10.11 | Male | 16 | - | - | - | 31.18 | - | - | - | - | - | - | 31.09 | - | - | - | - | - | - | - | - | 31.07 |
| 186 | 23.10.11 | Female | 26 | - | - | - | 34.46 | - | - | - | - | - | - | - | - | - | - | - | - | - | - | - | 34.28 |
| 187 | 23.10.11 | Male | 52 | - | - | - | 25.86 | - | - | - | - | 28.56 | - | - | - | - | - | - | - | - | - | - | 26.65 |
| 188 | 23.10.11 | Male | 45 | - | - | - | 33.09 | - | - | - | - | - | - | - | - | - | - | - | - | - | - | - | 32.37 |
| 189 | 23.10.12 | Male | 7 | 31.8 | - | - | 30.84 | - | - | - | - | - | - | - | - | - | - | - | - | - | - | 36.09 | 30.15 |
| 190 | 23.10.12 | Male | 14 | - | - | - | 31.22 | - | - | - | - | 29.55 | - | - | - | - | - | - | - | - | - | - | 32.17 |
| 191 | 23.10.12 | Female | 28 | - | - | 29.84 | 26.95 | - | - | - | - | - | - | - | - | - | - | - | - | - | 34.15 | - | 27.32 |
| 192 | 23.10.12 | Female | 52 | - | - | - | 32 | - | - | - | - | - | - | - | - | - | - | - | 31.65 | - | - | - | 31.22 |
| 193 | 23.10.12 | Male | 13 | - | - | - | 33.16 | - | - | - | - | - | - | - | - | - | - | - | - | - | - | - | 32.21 |
| 194 | 23.10.12 | Female | 8 | - | - | - | 30.46 | - | - | - | - | - | - | - | - | - | - | - | - | - | - | - | 31.61 |
| 195 | 23.10.13 | Female | 54 | - | - | - | 27.97 | - | - | - | - | - | - | - | - | - | - | - | - | - | - | - | 28.76 |
| 196 | 23.10.13 | Female | 22 | - | - | - | 32.68 | - | - | - | - | - | - | - | - | - | - | - | - | - | - | - | 32.15 |
| 197 | 23.10.13 | Female | 31 | - | - | - | 28.97 | - | - | 29.67 | - | - | - | - | - | - | - | - | - | - | - | - | 28.75 |
| 198 | 23.10.13 | Male | 31 | - | - | - | 30.73 | - | - | - | - | - | - | - | - | - | - | - | 22.93 | - | - | - | 30.67 |
| 199 | 23.10.13 | Female | 32 | - | - | - | 27.11 | - | - | - | - | - | - | - | - | - | - | - | - | - | - | - | 27.57 |
| 200 | 23.10.13 | Female | 27 | - | - | - | 30.92 | - | - | - | - | - | - | - | - | - | - | - | - | - | - | - | 31.51 |
| 201 | 23.10.16 | Male | 72 | - | - | - | 29.1 | 24.53 | - | - | - | - | - | - | - | - | - | - | - | 22.38 | - | - | 28.46 |
| 202 | 23.10.16 | Male | 4 | - | - | - | 32.17 | - | - | - | - | - | - | - | - | - | - | - | - | - | - | - | 33.43 |
| 203 | 23.10.16 | Male | 2 | - | - | - | 30.78 | - | - | - | - | - | - | - | - | - | - | - | - | - | - | - | 33.14 |
| 204 | 23.10.16 | Male | 10 | 26.79 | - | - | 30.21 | - | - | - | - | - | - | - | - | - | - | - | - | - | - | 29.38 | 31.81 |
| 205 | 23.10.17 | Male | 20 | - | - | 30.17 | 29.05 | - | - | - | - | - | - | - | - | - | - | - | - | - | 34.14 | - | 30.22 |
| 206 | 23.10.17 | Female | 6 | - | - | - | 29.16 | - | - | 22.65 | - | 24.69 | - | - | - | - | - | - | - | - | - | - | 30.36 |
| 207 | 23.10.17 | Male | 14 | - | - | - | 25.96 | - | - | - | - | - | - | - | - | - | - | - | - | - | - | - | 29.21 |
| 208 | 23.10.17 | Female | 9 | - | - | - | 26.53 | - | - | - | - | - | - | - | - | - | - | - | - | - | - | - | 30.4 |
| 209 | 23.10.17 | Male | 19 | - | - | - | 29.23 | - | - | - | - | - | - | - | - | - | - | - | - | - | - | - | 31.27 |
| 210 | 23.10.17 | Male | 9 | - | - | - | 28.77 | - | - | - | - | - | - | - | - | - | - | - | - | - | - | - | 32.07 |
| 211 | 23.10.17 | Female | 44 | - | - | - | 26.65 | - | - | - | - | - | - | - | - | - | - | - | - | - | - | - | 27.06 |
| 212 | 23.10.19 | Male | 7 | - | - | - | 32.44 | - | - | - | - | - | - | - | - | - | - | 33.47 | - | - | - | - | 32.66 |
| 213 | 23.10.19 | Male | 21 | - | - | 30.66 | 31.04 | - | - | - | - | - | - | - | - | - | - | - | - | - | 29.88 | - | 31.21 |
| 214 | 23.10.19 | Female | 20 | - | - | 27.53 | 29.91 | - | - | - | - | - | - | - | - | - | - | - | - | - | 29.34 | - | 31.97 |
| 215 | 23.10.19 | Male | 44 | 30.41 | - | - | 31.18 | - | - | - | - | - | - | - | - | - | - | - | 34.81 | - | - | 34.06 | 32.29 |
| 216 | 23.10.19 | Female | 8 | - | - | - | 29.13 | - | - | - | - | - | - | - | - | - | - | - | 30.95 | - | - | - | 29.72 |
| 217 | 23.10.19 | Female | 40 | - | - | - | 29.04 | - | - | - | - | - | - | - | - | - | - | - | - | - | - | - | 28.23 |
| 218 | 23.10.19 | Female | 29 | - | - | - | 29.69 | - | - | - | - | - | - | - | - | - | - | - | - | - | - | - | 30.12 |
| 219 | 23.10.19 | Male | 12 | - | - | - | 32.11 | - | - | - | - | - | - | - | - | - | - | - | - | - | - | - | 34.07 |
| 220 | 23.10.19 | Male | 9 | 27.8 | - | - | 29.75 | - | - | - | - | - | - | - | - | - | - | - | - | - | - | 30.85 | 30.21 |
| 221 | 23.10.19 | Male | 20 | - | - | 28.36 | 30.41 | - | - | - | - | - | - | - | - | - | - | - | - | - | 30.65 | - | 31.73 |
| 222 | 23.10.19 | Female | 10 | - | - | - | 30.21 | - | - | - | - | - | - | - | - | - | - | - | - | - | - | - | 30.86 |
| 223 | 23.10.19 | Female | 23 | - | - | - | 32.21 | - | - | - | - | - | - | - | - | - | - | - | - | - | - | - | 32.65 |
| 224 | 23.10.19 | Male | 57 | - | - | - | 29.59 | - | - | - | - | - | 21.1 | - | - | - | - | - | - | - | - | - | 30.2 |
| 225 | 23.10.20 | Male | 50 | - | - | - | 26.15 | - | - | - | - | - | - | - | - | - | - | - | - | - | - | - | 26.11 |
| 226 | 23.10.20 | Female | 36 | - | - | - | 28.52 | - | - | - | - | - | - | - | - | - | - | - | - | - | - | - | 29.18 |
| 227 | 23.10.20 | Female | 55 | - | - | - | 30.23 | - | - | - | - | - | - | - | - | - | - | - | - | - | - | - | 31.73 |
| 228 | 23.10.20 | Male | 13 | - | - | - | 32.85 | - | - | - | - | - | - | - | - | - | - | - | - | - | - | - | 34.05 |
| 229 | 23.10.23 | Female | 46 | - | - | - | 33.16 | - | - | - | - | - | - | - | - | - | - | - | - | - | - | - | 33.97 |
| 230 | 23.10.23 | Male | 9 | - | - | - | 31.89 | - | - | - | - | - | - | - | - | - | - | - | - | - | - | - | 33.9 |
| 231 | 23.10.23 | Female | 8 | - | - | 30.63 | 30.87 | - | - | - | - | - | - | - | - | - | - | - | - | - | 38.64 | - | 33.46 |
| 232 | 23.10.23 | Female | 59 | - | - | - | 28.62 | - | - | - | - | - | - | - | - | - | - | - | - | - | - | - | 29.45 |
| 233 | 23.10.23 | Male | 36 | 32.78 | - | - | 30.6 | - | - | - | - | - | - | - | - | - | - | - | - | - | - | - | 30.34 |
| 234 | 23.10.23 | Male | 57 | 27.74 | - | - | 29.66 | - | - | - | - | - | - | - | - | - | - | - | - | - | - | 28.85 | 31.25 |
| 235 | 23.10.23 | Female | 21 | - | - | 28.17 | 32.68 | - | - | - | - | - | - | 32.06 | - | - | - | - | - | - | 27.13 | - | 34.31 |
| 236 | 23.10.23 | Female | 36 | - | - | - | 31.17 | - | - | - | - | - | - | - | - | - | - | - | - | - | - | - | 30.6 |
| 237 | 23.10.23 | Female | 25 | - | - | - | 33.68 | - | - | - | - | - | - | - | - | - | - | - | - | - | - | - | 34.85 |
| 238 | 23.10.23 | Female | 12 | - | - | - | 30.82 | - | - | - | - | - | - | - | - | - | - | - | - | - | - | - | 30.91 |
| 239 | 23.10.23 | Female | 30 | - | - | - | 29.08 | - | - | - | - | - | - | - | - | - | - | - | - | - | - | - | 30.57 |
| 240 | 23.10.23 | Female | 20 | - | - | - | 26.87 | - | - | - | - | - | - | - | - | - | - | - | - | - | - | - | 27.11 |
| 241 | 23.10.23 | Female | 17 | - | - | - | 30.66 | - | - | - | - | - | - | - | - | - | - | - | - | - | - | - | 31.58 |
| 242 | 23.10.23 | Male | 20 | - | - | - | 28.05 | - | - | - | - | - | - | - | - | - | - | - | - | - | - | - | 28.1 |
| 243 | 23.10.23 | Male | 49 | - | - | - | 28.1 | - | - | - | - | - | - | - | - | - | - | - | - | - | - | - | 29.06 |
| 244 | 23.10.23 | Male | 27 | - | - | - | 30.48 | - | - | - | - | - | - | - | - | - | - | - | - | - | - | - | 30.11 |
| 245 | 23.10.25 | Male | 6 | - | - | - | 32.26 | - | - | - | - | - | - | - | - | - | - | - | - | - | - | - | 35.01 |
| 246 | 23.10.25 | Male | 25 | 28.97 | - | - | 29.59 | - | - | - | - | - | - | - | - | - | - | - | - | - | - | 29.3 | 30.83 |
| 247 | 23.10.25 | Female | 34 | 28.83 | - | - | 29.84 | - | - | - | - | - | - | - | - | - | - | - | - | - | - | 29.87 | 30.85 |
| 248 | 23.10.25 | Female | 60 | - | - | - | 29.65 | - | - | - | - | - | - | - | - | - | - | - | - | - | - | - | 28.82 |
| 249 | 23.10.25 | Male | 11 | 33.51 | - | - | 30.22 | - | - | - | - | - | - | - | - | - | - | - | - | - | - | 37.03 | 32.29 |
| 250 | 23.10.25 | Female | 14 | - | - | - | 31.48 | - | - | - | - | - | - | - | - | - | - | - | - | - | - | - | 32.35 |
| 251 | 23.10.25 | Male | 60 | - | - | - | 29.98 | - | - | - | - | - | - | - | - | - | - | - | - | - | - | - | 31.33 |
| 252 | 23.10.25 | Male | 16 | - | - | - | 30.88 | - | - | - | - | - | - | - | - | - | - | - | - | - | - | - | 31.13 |
| 253 | 23.10.25 | Male | 3 | - | - | - | 31.4 | - | 28.83 | - | - | - | - | - | - | - | - | - | - | - | - | - | 32.7 |
| 254 | 23.10.25 | Male | 7 | - | - | - | 28.82 | - | - | - | - | - | - | 27.7 | - | - | - | - | - | - | - | - | 29.63 |
| 255 | 23.10.25 | Female | 32 | - | - | - | 25.77 | - | - | - | - | - | - | - | - | - | - | - | - | - | - | - | 26.92 |
| 256 | 23.10.25 | Male | 3 | - | - | - | 33.56 | - | - | 26.73 | - | 28.31 | - | - | - | - | - | - | - | - | - | - | 35.2 |
| 257 | 23.10.25 | Female | 40 | 32.45 | - | - | 34.93 | - | - | - | - | - | - | - | - | - | - | - | - | - | - | 32.48 | 34.41 |
| 258 | 23.10.25 | Male | 20 | 24.74 | - | - | 29.78 | - | - | - | - | - | - | - | - | - | - | - | - | - | - | 24.05 | 31.67 |
| 259 | 23.10.25 | Female | 43 | - | - | - | 28.9 | - | - | - | - | - | - | - | - | - | - | - | - | - | - | - | 28.86 |
| 260 | 23.10.25 | Male | 15 | - | - | - | 29.42 | - | - | - | - | - | - | - | - | - | - | - | - | - | - | - | 29.08 |
| 261 | 23.10.25 | Male | 12 | - | - | - | 34.04 | - | - | - | - | - | - | - | - | - | - | - | - | - | - | - | 35.8 |
| 262 | 23.10.25 | Male | 11 | 26.57 | - | - | 29.55 | - | - | - | - | - | - | - | - | - | - | - | - | - | - | 27.35 | 31.94 |
| 263 | 23.10.25 | Male | 17 | - | - | - | 30.76 | - | - | - | - | - | - | - | - | - | - | - | - | - | - | - | 30.61 |
| 264 | 23.10.25 | Female | 20 | 30.91 | - | - | 33.31 | - | - | - | - | - | - | - | - | - | - | - | - | - | - | 31.34 | 33.69 |
| 265 | 23.10.25 | Female | 3 | 28.53 | - | - | 32.56 | - | - | - | - | - | - | - | - | - | - | - | - | - | - | 29.61 | 33 |
| 266 | 23.10.25 | Male | 14 | - | - | 27.94 | 28.67 | - | - | - | - | - | - | - | - | - | - | - | - | - | 33.91 | - | 29.62 |
| 267 | 23.10.25 | Male | 17 | - | - | 32.5 | 34.49 | - | - | - | - | - | - | - | - | - | - | - | - | - | 32.32 | - | 34.99 |
| 268 | 23.10.25 | Female | 24 | 31.03 | - | - | 29.94 | - | - | - | - | - | - | - | - | - | - | - | - | - | - | 30.88 | 29.53 |
| 269 | 23.10.25 | Male | 9 | - | - | - | 29.84 | - | - | - | - | 26.81 | - | - | - | - | - | - | - | - | - | - | 31.03 |
| 270 | 23.10.26 | Female | 67 | - | - | - | 27.78 | - | - | - | - | - | - | - | - | - | - | - | - | - | - | - | 29.67 |
| 271 | 23.10.26 | Female | 41 | - | - | - | 30.64 | - | - | - | - | - | - | - | - | - | - | - | - | - | - | - | 31.85 |
| 272 | 23.10.26 | Female | 49 | - | - | - | 27.97 | - | - | - | - | - | - | - | - | - | - | - | - | - | - | - | 28.77 |
| 273 | 23.10.26 | Female | 40 | - | - | - | 30.81 | - | - | - | - | - | - | - | - | - | - | - | 29.87 | - | - | - | 30.33 |
| 274 | 23.10.26 | Male | 13 | 24.08 | - | - | 27.14 | - | - | - | - | - | - | - | - | - | - | - | - | - | - | 26.22 | 30.32 |
| 275 | 23.10.26 | Male | 4 | - | - | - | 27.54 | - | - | - | 23.83 | - | - | - | - | - | - | - | - | - | - | - | 31.71 |
| 276 | 23.10.26 | Male | 40 | - | - | - | 30.72 | - | - | - | - | - | - | - | - | - | - | - | - | - | - | - | 31.08 |
| 277 | 23.10.26 | Female | 24 | 33.19 | - | - | 33.09 | - | - | - | - | - | - | - | - | - | - | - | - | - | - | 36.92 | 32.91 |
| 278 | 23.10.26 | Male | 28 | - | - | 27.12 | 26.12 | - | - | - | - | - | - | - | - | - | - | - | - | - | 31.56 | - | 26.77 |
| 279 | 23.10.26 | Male | 7 | - | - | - | 27.95 | - | - | - | - | - | - | - | - | - | - | - | - | - | - | - | 27.79 |
| 280 | 23.10.27 | Male | 12 | - | - | - | 31.7 | - | - | - | - | - | - | - | - | - | - | - | - | - | - | - | 33.53 |
| 281 | 23.10.27 | Male | 25 | - | - | 29.81 | 30.14 | - | - | - | - | - | - | - | - | - | - | - | - | - | 35.67 | - | 32.27 |
| 282 | 23.10.27 | Male | 17 | - | - | - | 30.89 | - | - | - | - | 26.65 | - | - | - | - | - | - | - | - | - | - | 32.4 |
| 283 | 23.10.27 | Male | 6 | - | - | - | 30.39 | - | - | - | - | - | - | - | - | - | - | - | - | - | - | - | 31.26 |
| 284 | 23.10.27 | Male | 2 | - | - | - | 29.85 | - | - | - | - | - | - | - | - | - | - | - | - | - | - | - | 30.8 |
| 285 | 23.10.27 | Male | 1 | - | - | 31.3 | 31.97 | - | - | - | - | - | - | - | - | - | - | - | - | - | 33.43 | - | 33.4 |
| 286 | 23.10.27 | Male | 57 | - | - | - | 30.84 | - | - | - | - | - | - | - | - | - | - | - | - | - | - | - | 31.81 |
| 287 | 23.10.27 | Male | 15 | - | - | - | 31.55 | - | - | - | - | - | - | - | - | - | - | - | - | - | - | - | 32.47 |
| 288 | 23.10.27 | Female | 7 | - | - | - | 31.63 | - | - | - | - | - | - | - | - | - | - | - | - | - | - | - | 32.23 |
| 289 | 23.10.27 | Female | 3 | - | - | 31.62 | 30.42 | - | - | - | - | - | - | - | - | - | - | - | - | - | 35.61 | - | 30.18 |
| 290 | 23.10.27 | Female | 36 | - | - | - | 31.9 | - | - | - | - | - | - | - | - | - | - | - | - | - | - | - | 33.82 |
| 291 | 23.10.27 | Female | 37 | - | - | - | 31.99 | - | - | - | - | - | - | - | - | - | - | - | - | - | - | - | 34.33 |
| 292 | 23.10.27 | Female | 2 | - | - | - | 31.82 | - | - | 28.8 | - | 29.63 | - | - | - | - | - | - | - | - | - | - | 32.73 |
| 293 | 23.10.27 | Male | 19 | - | - | - | 32.28 | - | - | - | - | - | - | - | - | - | - | - | - | - | - | - | 34.71 |
| 294 | 23.10.27 | Male | 5 | - | - | - | 31.61 | - | - | - | - | - | - | - | - | - | - | - | - | - | - | - | 32.99 |
| 295 | 23.10.27 | Male | 50 | - | - | - | 30.59 | - | - | - | - | - | - | - | - | - | - | - | - | - | - | - | 31.48 |
| 296 | 23.10.27 | Female | 5 | - | - | - | 31.48 | - | - | - | - | - | - | - | - | - | 28.61 | - | - | - | - | - | 32.02 |
| 297 | 23.10.27 | Female | 26 | - | - | 33.26 | 31.82 | - | - | - | - | - | - | - | - | - | - | - | - | - | 37.5 | - | 31.7 |
| 298 | 23.10.27 | Female | 33 | - | - | - | 33.19 | - | - | - | - | - | - | - | - | - | - | - | - | - | - | - | 35.32 |
| 299 | 23.10.27 | Female | 13 | - | - | - | 30.65 | - | - | - | - | - | - | - | - | - | - | - | - | - | - | - | 31.09 |
| 300 | 23.10.27 | Male | 4 | - | - | - | 30.78 | - | - | - | - | - | - | - | - | - | - | - | - | - | - | - | 32.1 |
| 301 | 23.10.27 | Male | 17 | - | - | 29.79 | 31.09 | - | - | - | - | - | - | - | - | - | - | - | - | - | 30.36 | - | 32.39 |
| 302 | 23.10.27 | Male | 14 | - | - | - | 28.39 | - | - | - | - | - | - | - | - | - | - | - | - | - | - | - | 28.98 |
| 303 | 23.10.27 | Male | 48 | - | - | 29.8 | 28.89 | - | - | - | - | - | - | - | - | - | - | - | - | - | 38.13 | - | 29.99 |
| 304 | 23.10.27 | Female | 64 | - | - | - | 26.35 | - | - | - | 26.42 | - | - | - | - | - | - | - | - | - | - | - | 26.14 |
| 305 | 23.10.27 | Male | 1 | - | - | 28.01 | 30.62 | - | - | - | - | - | - | - | - | - | - | - | - | - | 28.89 | - | 31.67 |
| 306 | 23.10.27 | Male | 20 | - | - | - | 28.84 | - | - | - | - | - | - | - | - | - | - | - | - | - | - | - | 28.97 |
| 307 | 23.10.30 | Female | 6 | - | - | - | 29.5 | - | - | - | - | - | - | - | - | - | - | - | - | - | - | - | 30.99 |
| 308 | 23.10.30 | Female | 12 | - | - | - | 33.37 | - | - | - | - | - | - | - | - | - | - | - | - | - | - | - | 34.63 |
| 309 | 23.10.30 | Female | 9 | - | - | - | 33.64 | - | - | - | - | - | - | - | - | - | - | - | - | - | - | - | 33.89 |
| 310 | 23.10.30 | Female | 7 | - | - | - | 30.05 | - | - | - | - | 26.23 | - | - | - | - | - | - | - | - | - | - | 31.7 |
| 311 | 23.10.30 | Male | 7 | - | - | - | 30.76 | - | - | - | - | - | - | 28.64 | - | - | - | - | - | - | - | - | 32.06 |
| 312 | 23.10.30 | Female | 7 | - | - | - | 32.5 | - | - | 27.39 | - | 29.5 | 34.18 | - | - | - | - | - | - | - | - | - | 33.06 |
| 313 | 23.10.30 | Female | 3 | - | - | - | 30.06 | - | - | 30.38 | - | 27.66 | - | - | - | - | - | - | - | - | - | - | 29.62 |
| 314 | 23.10.30 | Female | 8 | - | - | - | 28.19 | - | - | - | - | - | - | - | - | - | - | - | - | - | - | - | 29.41 |
| 315 | 23.10.30 | Female | 8 | 28.99 | - | - | 29.79 | - | - | - | - | - | - | - | - | - | - | - | - | - | - | 31.41 | 29.35 |
| 316 | 23.10.30 | Male | 3 | - | - | - | 29.15 | - | - | - | - | - | - | - | - | - | - | - | - | - | - | - | 31.25 |
| 317 | 23.10.30 | Female | 12 | - | - | - | 32.52 | - | - | - | - | 31 | - | - | - | - | - | - | - | - | - | - | 33.84 |
| 318 | 23.10.30 | Male | 11 | - | - | - | 32.69 | - | - | - | - | - | - | - | - | - | - | - | - | - | - | - | 32.29 |
| 319 | 23.10.30 | Female | 6 | - | - | - | 32.78 | - | - | - | - | - | - | - | - | - | - | - | - | - | - | - | 33.07 |
| 320 | 23.10.30 | Male | 7 | - | - | - | 27.64 | - | - | - | - | - | - | - | - | - | - | - | - | - | - | - | 29.93 |
| 321 | 23.10.30 | Female | 4 | - | - | - | 30.62 | - | - | - | 31.69 | - | - | - | - | - | - | - | - | - | - | - | 32.88 |
| 322 | 23.10.30 | Male | 37 | 28.65 | - | - | 26.1 | - | - | - | - | - | - | - | - | - | - | - | - | - | - | 31.91 | 25.62 |
| 323 | 23.10.30 | Male | 16 | - | - | - | 30.81 | - | - | - | - | - | - | - | - | - | - | - | - | - | - | - | 33.69 |
| 324 | 23.10.30 | Female | 6 | - | - | - | 31.61 | - | - | - | - | 28.89 | - | - | - | 26.01 | - | - | - | - | - | - | 33.06 |
| 325 | 23.10.30 | Female | 31 | - | - | - | 32.37 | - | - | - | - | - | - | - | - | - | - | - | - | - | - | - | 32.11 |
| 326 | 23.10.30 | Male | 30 | - | - | - | 27.62 | - | - | - | - | - | - | - | - | - | - | - | - | - | - | - | 27.58 |
| 327 | 23.10.30 | Female | 44 | 32.5 | - | - | 30.97 | - | - | - | - | - | - | - | - | - | - | - | - | - | - | - | 32.18 |
| 328 | 23.10.31 | Male | 15 | - | - | - | 32.78 | - | - | - | - | - | - | - | - | - | - | - | - | - | - | - | 33.26 |
| 329 | 23.10.31 | Female | 10 | 30.38 | - | - | 31.12 | - | - | - | - | - | - | - | - | - | - | - | - | - | - | 36.75 | 32.07 |
| 330 | 23.10.31 | Female | 30 | - | - | - | 27.84 | - | - | - | - | - | - | 26 | - | - | - | - | - | - | - | - | 29.37 |
| 331 | 23.10.31 | Female | 37 | 29.68 | - | - | 29.78 | - | - | - | - | - | - | - | - | - | - | - | - | - | - | 30.86 | 30.76 |
| 332 | 23.10.31 | Female | 34 | - | - | - | 28.04 | - | - | - | 28.51 | - | - | - | - | - | - | - | - | - | - | - | 27.96 |
| 333 | 23.10.31 | Male | 6 | - | - | 32.11 | 31.85 | - | - | - | - | - | - | - | - | - | - | - | - | - | 32.11 | - | 31.7 |
| 334 | 23.10.31 | Male | 45 | - | - | - | 32.58 | - | - | - | - | - | - | - | - | - | - | - | 32.94 | - | - | - | 34.04 |
| 335 | 23.10.31 | Female | 22 | - | - | - | 33.15 | - | - | - | - | - | - | - | - | - | - | - | - | - | - | - | 33.34 |
| 336 | 23.10.31 | Female | 21 | - | - | - | 31.97 | - | - | - | - | - | - | - | - | - | - | - | - | - | - | - | 33.85 |
| 337 | 23.10.31 | Female | 8 | - | - | - | 32.52 | - | - | - | - | - | - | - | - | - | - | - | - | - | - | - | 33.05 |
| 338 | 23.10.31 | Male | 10 | 30.73 | - | - | 32.06 | - | - | - | - | - | - | - | - | - | - | - | - | - | - | 31.9 | 32.94 |
| 339 | 23.10.31 | Female | 20 | - | - | - | 29.88 | - | - | - | - | - | - | - | - | - | - | - | 30.62 | - | - | - | 30.73 |
| 340 | 23.10.31 | Female | 33 | 32.14 | - | - | 31.58 | - | - | - | - | - | - | - | - | - | - | - | - | - | - | 39.23 | 32.1 |
| 341 | 23.10.31 | Male | 69 | - | - | - | 28.13 | - | - | - | - | - | - | - | - | - | - | - | - | - | - | - | 28.23 |
| 342 | 23.10.31 | Male | 6 | - | - | - | 27.91 | - | - | - | - | - | - | - | - | - | - | - | - | - | - | - | 29.55 |
| 343 | 23.10.31 | Male | 15 | - | - | - | 27.96 | - | - | - | - | - | - | - | - | - | - | - | - | - | - | - | 29.1 |
| 344 | 23.10.31 | Male | 5 | 27.2 | - | - | 29.77 | - | - | - | - | - | - | 27.98 | - | - | - | - | - | - | - | 28.7 | 32.69 |
| 345 | 23.10.31 | Male | 65 | - | - | - | 29.49 | - | - | - | - | - | - | - | - | - | - | - | 27.2 | - | - | - | 29.05 |
| 346 | 23.10.31 | Male | 14 | - | - | - | 28.92 | - | - | - | - | - | - | - | - | - | - | - | - | - | - | - | 30.03 |
| 347 | 23.10.31 | Female | 5 | - | - | - | 29.12 | - | - | 26.99 | - | 26.49 | - | - | - | - | - | - | - | - | - | - | 30.96 |
| 348 | 23.10.31 | Male | 14 | - | - | - | 30.98 | - | - | - | - | - | - | 28.57 | - | - | - | - | - | - | - | - | 31.39 |
| 349 | 23.10.31 | Male | 8 | 28.25 | - | - | 31.97 | - | - | - | - | - | - | - | - | - | - | - | - | - | - | 29.3 | 33.58 |
| 350 | 23.10.31 | Female | 8 | - | - | - | 30.65 | - | - | - | - | - | - | - | - | - | - | - | - | - | - | - | 33.01 |
| 351 | 23.10.31 | Female | 13 | - | - | - | 30.02 | - | - | - | - | - | - | 28.35 | - | - | - | - | 30.87 | - | - | - | 32.28 |
| 352 | 23.10.31 | Male | 21 | 29.22 | - | - | 30.2 | - | - | - | - | - | - | - | - | - | - | - | - | - | - | 32.28 | 31.72 |
| 353 | 23.10.31 | Male | 12 | 29.71 | - | - | 28.8 | - | - | - | - | - | - | - | - | - | - | - | - | - | - | 30.2 | 28.82 |
| 354 | 23.10.31 | Male | 9 | - | - | - | 31.71 | - | - | - | - | - | - | - | - | - | - | - | - | - | - | - | 32.29 |
| 355 | 23.11.01 | Female | 8 | - | - | - | 27.67 | - | - | - | - | - | 34.41 | - | - | - | - | - | - | - | - | - | 28.32 |
| 356 | 23.11.01 | Female | 6 | - | 29.88 | - | 31.71 | - | - | - | - | - | - | - | - | - | - | - | - | - | - | - | 32.63 |
| 357 | 23.11.01 | Female | 8 | - | - | - | 28.15 | - | - | - | - | - | - | 28.7 | - | - | - | - | - | - | - | - | 29.2 |
| 358 | 23.11.01 | Female | 11 | 33.6 | - | - | 32.57 | - | - | - | - | - | - | - | - | - | - | - | - | - | - | - | 32.38 |
| 359 | 23.11.01 | Male | 5 | - | - | 28.25 | 26.65 | - | - | - | - | - | - | - | - | - | - | - | - | - | 31.73 | - | 27.76 |
| 360 | 23.11.01 | Female | 14 | - | - | - | 29.99 | - | - | - | - | - | - | - | - | - | - | - | - | - | - | - | 30.3 |
| 361 | 23.11.01 | Male | 11 | - | - | - | 30.1 | - | - | - | - | - | - | - | - | - | - | - | - | - | - | - | 29.68 |
| 362 | 23.11.01 | Female | 7 | - | - | 28.65 | 29.84 | - | - | - | - | - | - | - | - | - | - | - | - | - | 36.41 | - | 31.57 |
| 363 | 23.11.03 | Male | 39 | - | - | - | 28.04 | - | - | - | - | - | - | - | - | - | - | - | - | - | - | - | 28.13 |
| 364 | 23.11.03 | Male | 1 | - | - | - | 28.62 | - | - | - | - | - | - | - | - | - | - | - | - | - | - | - | 30.87 |
| 365 | 23.11.03 | Male | 31 | - | - | - | 29.61 | - | - | - | - | - | - | - | - | - | - | - | - | - | - | - | 28.91 |
| 366 | 23.11.03 | Male | 15 | - | - | - | 29.62 | - | - | - | - | - | - | - | - | - | - | - | - | - | - | - | 31.22 |
| 367 | 23.11.03 | Male | 10 | - | - | - | 31.59 | - | - | - | - | 30.09 | - | - | - | - | - | - | - | - | - | - | 32.17 |
| 368 | 23.11.03 | Male | 10 | - | - | - | 29.24 | - | - | - | - | - | - | - | - | - | - | - | - | - | - | - | 29.43 |
| 369 | 23.11.03 | Female | 6 | - | - | - | 33.55 | - | - | - | - | - | - | - | - | - | - | - | - | - | - | - | 33.53 |
| 370 | 23.11.03 | Male | 46 | - | - | - | 26.59 | - | - | - | - | - | - | - | - | 29.55 | - | - | - | - | - | - | 27.1 |
| 371 | 23.11.03 | Male | 6 | - | - | - | 31.43 | - | - | - | - | - | - | - | - | - | - | - | - | - | - | - | 32.11 |
| 372 | 23.11.06 | Female | 20 | - | - | - | 26.9 | 19.66 | - | - | - | - | - | - | - | - | - | - | - | 16.17 | - | - | 26.41 |
| 373 | 23.11.06 | Female | 10 | 31.74 | - | - | 30.13 | - | - | - | - | - | - | - | - | - | - | - | - | - | - | 33.85 | 29.62 |
| 374 | 23.11.06 | Male | 17 | - | - | - | 29.61 | - | - | - | - | - | - | - | - | - | - | - | - | - | - | - | 29.84 |
| 375 | 23.11.06 | Male | 7 | - | - | - | 28.78 | - | - | - | - | - | - | 28.26 | - | - | - | - | - | - | - | - | 29.14 |
| 376 | 23.11.06 | Female | 32 | - | - | - | 26.36 | - | - | - | - | - | - | - | - | - | - | - | - | - | - | - | 26.02 |
| 377 | 23.11.06 | Female | 39 | - | - | - | 26.44 | - | - | - | - | - | - | - | - | - | - | - | - | - | - | - | 26.25 |
| 378 | 23.11.07 | Male | 3 | - | - | - | 30.13 | - | - | - | - | 27.7 | - | - | - | - | - | - | - | - | - | - | 32.13 |
| 379 | 23.11.07 | Male | 5 | - | - | - | 26.38 | - | - | 24.24 | - | 24.42 | - | - | - | - | - | - | - | - | - | - | 27.04 |
| 380 | 23.11.07 | Male | 32 | - | - | - | 31.88 | - | - | - | - | - | - | - | - | - | - | - | - | - | - | - | 31.96 |
| 381 | 23.11.07 | Female | 1 | - | - | - | 31.45 | - | - | - | - | - | - | - | - | - | - | - | - | - | - | - | 33.17 |
| 382 | 23.11.07 | Male | 4 | - | - | - | 32.34 | - | - | - | - | - | - | - | - | - | - | - | - | - | - | - | 33.78 |
| 383 | 23.11.07 | Female | 4 | - | - | - | 30.18 | - | - | - | - | - | - | - | - | - | - | - | - | - | - | - | 31.57 |
| 384 | 23.11.07 | Male | 9 | - | - | - | 30.02 | - | - | - | - | - | - | - | - | - | - | - | - | - | - | - | 30.01 |
| 385 | 23.11.07 | Male | 4 | - | - | - | 30.69 | - | - | - | - | - | - | - | - | - | - | - | - | - | - | - | 28.03 |
| 386 | 23.11.07 | Female | 7 | - | - | - | 30.01 | - | - | - | - | - | - | - | - | 32.92 | - | - | - | - | - | - | 30.96 |
| 387 | 23.11.07 | Female | 5 | - | - | - | 30.67 | - | - | - | - | - | - | - | - | - | - | - | - | - | - | - | 31.61 |
| 388 | 23.11.07 | Male | 11 | 25.84 | - | - | 28.85 | - | - | - | - | - | - | - | - | - | - | - | - | - | - | 26.95 | 28.84 |
| 389 | 23.11.07 | Male | 5 | 31.39 | - | - | 31.16 | - | - | - | - | - | - | 28.87 | - | - | - | - | - | - | - | 32.23 | 30.89 |
| 390 | 23.11.07 | Female | 9 | 26.31 | - | - | 30.24 | - | - | - | - | - | - | - | - | - | - | - | - | - | - | 32.9 | 31.26 |
| 391 | 23.11.07 | Male | 3 | - | - | - | 30.7 | - | - | - | - | - | - | - | - | - | - | - | - | - | - | - | 32.1 |
| 392 | 23.11.07 | Female | 7 | 30.6 | - | - | 31.9 | - | - | - | - | - | - | - | - | - | - | - | - | - | - | - | 33.31 |
| 393 | 23.11.07 | Male | 14 | - | - | - | 31.88 | - | - | - | - | - | - | - | - | - | - | - | - | - | - | - | 32.11 |
| 394 | 23.11.07 | Female | 5 | - | - | - | 30.09 | - | - | - | - | - | - | - | - | - | - | - | - | - | - | - | 31.07 |
| 395 | 23.11.07 | Female | 35 | - | - | - | 29.66 | - | - | - | - | - | - | - | - | - | - | - | - | - | - | - | 30.75 |
| 396 | 23.11.07 | Female | 16 | - | - | - | 30.16 | - | - | - | - | - | - | - | - | - | - | - | - | - | - | - | 31.12 |
| 397 | 23.11.08 | Female | 8 | - | - | - | 30.49 | - | - | - | - | - | - | - | - | - | - | - | - | - | - | - | 32.23 |
| 398 | 23.11.08 | Female | 36 | - | - | - | 29.2 | - | - | 24.99 | - | 26.95 | - | - | - | - | - | - | - | - | - | - | 34.37 |
| 399 | 23.11.08 | Female | 10 | - | - | - | 31.83 | - | - | - | - | - | - | - | - | - | - | - | - | - | - | - | 33.04 |
| 400 | 23.11.08 | Male | 17 | - | - | - | 28.9 | - | - | - | - | - | - | - | - | - | - | - | - | - | - | - | 30.2 |
| 401 | 23.11.08 | Female | 9 | 27.14 | - | - | 27.31 | - | - | - | - | - | - | - | - | - | - | - | - | - | - | 28.48 | 27.17 |
| 402 | 23.11.08 | Male | 6 | - | - | - | 29.94 | - | - | - | - | - | - | - | - | - | - | - | - | - | - | - | 31.55 |
| 403 | 23.11.08 | Female | 13 | - | - | - | 28.13 | - | - | - | 29.18 | - | - | - | - | - | - | - | - | - | - | - | 28.76 |
| 404 | 23.11.08 | Female | 3 | - | - | - | 29.29 | - | - | - | - | - | - | - | - | - | - | 29.1 | - | - | - | - | 31.45 |
| 405 | 23.11.08 | Female | 35 | 29.31 | - | - | 27.47 | - | - | - | - | - | - | - | - | - | - | - | - | - | - | 36.95 | 29.11 |
| 406 | 23.11.08 | Male | 13 | - | - | - | 25.96 | - | - | - | - | - | - | - | - | - | - | - | - | - | - | - | 28.36 |
| 407 | 23.11.08 | Male | 15 | - | - | - | 28.83 | - | - | - | - | - | - | - | - | - | - | - | - | - | - | - | 31.58 |
| 408 | 23.11.08 | Female | 41 | - | - | - | 30.2 | - | - | - | - | - | - | - | - | - | - | - | 33.75 | - | - | - | 30.55 |
| 409 | 23.11.08 | Female | 17 | - | - | - | 32.45 | - | - | - | - | 30.2 | - | - | - | - | - | - | - | - | - | - | 31.07 |
| 410 | 23.11.08 | Female | 25 | - | - | - | 32.53 | - | - | - | - | - | - | - | - | - | - | - | - | - | - | - | 33.03 |
| 411 | 23.11.08 | Female | 37 | - | - | - | 26.74 | - | - | - | - | - | - | - | - | - | - | - | - | - | - | - | 27.93 |
| 412 | 23.11.08 | Male | 12 | 27.78 | - | - | 28.46 | - | - | - | - | - | - | - | - | - | - | - | - | - | - | 29.73 | 29.26 |
| 413 | 23.11.09 | Female | 6 | - | - | - | 33.43 | - | - | - | - | 32 | 37.68 | - | - | - | - | - | - | - | - | - | 32.94 |
| 414 | 23.11.09 | Female | 68 | - | - | 29.94 | 30.91 | - | - | - | - | - | - | - | - | - | - | - | - | - | 30.74 | - | 30.94 |
| 415 | 23.11.09 | Female | 10 | 32.83 | - | - | 32.05 | - | - | - | - | - | - | - | - | - | - | - | - | - | - | 37.17 | 32.55 |
| 416 | 23.11.09 | Female | 2 | - | - | - | 32.8 | - | - | - | - | - | - | - | - | - | - | - | - | - | - | - | 34.17 |
| 417 | 23.11.09 | Female | 6 | - | - | - | 32.93 | - | - | 28.08 | - | - | - | - | - | - | - | - | - | - | - | - | 34.6 |
| 418 | 23.11.09 | Female | 10 | - | - | - | 30.22 | - | - | - | - | - | - | 29.1 | - | - | - | - | - | - | - | - | 31.57 |
| 419 | 23.11.09 | Male | 10 | - | - | - | 31.99 | - | - | - | - | - | - | 30.74 | - | - | - | - | - | - | - | - | 33.72 |
| 420 | 23.11.09 | Female | 2 | - | - | - | 32.78 | - | - | - | - | - | - | 32.75 | - | - | - | - | - | - | - | - | 34.94 |
| 421 | 23.11.09 | Male | 36 | - | - | - | 28.7 | - | - | - | - | - | - | - | - | - | - | 29.52 | - | - | - | - | 28.07 |
| 422 | 23.11.09 | Male | 9 | - | - | - | 30.26 | - | - | - | - | - | - | 29.33 | - | - | - | - | - | - | - | - | 30.78 |
| 423 | 23.11.09 | Male | 34 | - | - | - | 29.04 | - | - | - | - | - | - | - | - | - | - | - | - | - | - | - | 31.46 |
| 424 | 23.11.09 | Female | 20 | - | - | - | 30.84 | - | - | - | - | - | - | - | - | - | - | - | 32.01 | - | - | - | 31.54 |
| 425 | 23.11.09 | Male | 57 | - | - | - | 28.91 | - | - | - | 28.03 | - | - | - | - | - | - | - | - | - | - | - | 29.52 |
| 426 | 23.11.09 | Male | 11 | - | - | - | 31.58 | - | - | - | - | - | - | - | - | - | - | - | - | - | - | - | 31.68 |
| 427 | 23.11.09 | Male | 6 | 39.04 | - | - | 32.42 | - | - | - | - | - | - | - | - | - | - | - | - | - | - | 37.74 | 32 |
| 428 | 23.11.09 | Male | 13 | - | - | - | 31.54 | - | - | - | - | - | - | - | - | - | - | - | - | - | - | - | 31.29 |
| 429 | 23.11.09 | Male | 6 | 33.16 | - | - | 32.99 | - | - | - | - | - | - | - | - | - | - | - | - | - | - | - | 32.8 |
| 430 | 23.11.09 | Male | 13 | 29.71 | - | - | 31.49 | - | - | - | - | - | - | - | - | - | - | - | - | - | - | 31.23 | 33.67 |
| 431 | 23.11.09 | Male | 1 | - | - | 31.69 | 32.03 | - | - | - | - | - | - | - | - | - | - | - | - | - | 31.92 | - | 32.44 |
| 432 | 23.11.09 | Male | 11 | - | - | - | 32.34 | - | - | - | 28.83 | - | - | - | - | - | - | - | - | - | - | - | 33.02 |
| 433 | 23.11.09 | Male | 62 | 31.74 | - | - | 29.59 | - | - | - | - | - | - | - | - | - | - | - | - | - | - | - | 29.79 |
| 434 | 23.11.09 | Female | 5 | - | - | - | 31.04 | - | - | - | - | - | - | 29.67 | - | - | - | - | - | - | - | - | 31.57 |
| 435 | 23.11.09 | Male | 7 | 28.54 | - | - | 31.21 | - | - | - | - | - | - | - | - | - | - | - | - | - | - | 31.65 | 30.6 |
| 436 | 23.11.09 | Male | 17 | - | - | - | 29.92 | - | - | - | - | - | - | - | - | - | - | - | - | - | - | - | 30.73 |
| 437 | 23.11.09 | Male | 19 | - | - | 30.61 | 31.15 | - | - | - | - | - | - | - | - | - | - | - | - | - | 35.65 | - | 32.4 |
| 438 | 23.11.09 | Female | 27 | - | - | - | 29.68 | - | - | - | - | - | - | 28.77 | - | - | - | - | - | - | - | - | 30.37 |
| 439 | 23.11.09 | Female | 34 | - | - | - | 31.11 | - | - | - | - | - | - | - | - | - | - | - | - | - | - | - | 31.17 |
| 440 | 23.11.09 | Male | 20 | - | - | - | 29.33 | - | - | - | - | - | - | - | - | - | - | - | - | - | - | - | 30.75 |
| 441 | 23.11.10 | Male | 5 | - | - | - | 29.83 | - | - | - | - | - | - | - | - | - | - | - | - | - | - | - | 31.05 |
| 442 | 23.11.10 | Female | 7 | - | - | - | 30.71 | - | - | - | - | - | - | - | - | - | - | - | - | - | - | - | 31.06 |
| 443 | 23.11.10 | Female | 3 | - | - | - | 26.56 | - | - | 25 | 22.59 | - | - | - | - | - | - | - | - | - | - | - | 26.34 |
| 444 | 23.11.10 | Female | 6 | - | - | - | 30.7 | - | - | - | - | - | - | - | - | - | - | - | - | - | - | - | 31.51 |
| 445 | 23.11.10 | Female | 16 | - | - | - | 32.03 | - | - | - | - | - | - | - | - | - | - | - | - | - | - | - | 33.22 |
| 446 | 23.11.10 | Female | 5 | - | - | - | 30.07 | - | - | - | - | - | - | - | - | - | - | - | - | - | - | - | 31.6 |
| 447 | 23.11.10 | Male | 1 | - | - | - | 32.58 | - | - | - | - | - | - | - | - | - | - | - | - | - | - | - | 34.81 |
| 448 | 23.11.10 | Male | 39 | - | - | - | 29.47 | - | - | - | - | - | - | - | - | - | - | - | - | - | - | - | 29.55 |
| 449 | 23.11.10 | Male | 10 | - | - | - | 33.2 | - | - | - | - | - | - | - | - | - | - | - | - | - | - | - | 33.01 |
| 450 | 23.11.10 | Male | 16 | - | - | - | 32.97 | - | - | - | - | - | - | - | - | - | - | - | 30.83 | - | - | - | 33.02 |
| 451 | 23.11.10 | Male | 8 | 30.36 | - | - | 29.79 | - | - | - | - | - | - | - | - | - | - | - | - | - | - | 32.64 | 32.75 |
| 452 | 23.11.10 | Female | 5 | - | - | - | 30.28 | - | - | - | - | - | - | 27.43 | - | - | - | - | - | - | - | - | 31.12 |
| 453 | 23.11.10 | Female | 21 | - | - | - | 31.46 | - | - | - | - | - | - | - | - | - | - | - | - | - | - | - | 33.27 |
| 454 | 23.11.10 | Male | 15 | - | - | - | 32.19 | - | - | - | - | - | - | - | - | - | - | - | - | - | - | - | 33.04 |
| 455 | 23.11.10 | Male | 2 | - | - | - | 25.54 | - | - | - | - | - | - | - | - | - | - | - | - | - | - | - | 26.86 |
| 456 | 23.11.10 | Male | 10 | 31.85 | - | - | 30.62 | - | - | - | - | - | - | - | - | - | - | - | - | - | - | 36.41 | 31.07 |
| 457 | 23.11.10 | Male | 36 | - | - | - | 26.72 | - | - | - | - | - | - | - | - | - | - | - | - | - | - | - | 27.29 |
| 458 | 23.11.10 | Male | 7 | - | - | - | 31.48 | - | - | - | - | - | - | 30.86 | - | - | - | - | - | - | - | - | 31.64 |
| 459 | 23.11.10 | Female | 22 | 31.97 | - | - | 31.63 | - | - | - | - | - | - | - | - | - | - | - | - | - | - | 34.91 | 33.75 |
| 460 | 23.11.10 | Male | 7 | - | - | - | 30.82 | - | - | - | - | - | - | 28.57 | - | - | - | - | - | - | - | - | 30.96 |
| 461 | 23.11.10 | Female | 38 | - | - | - | 29.1 | - | - | - | - | - | - | - | - | - | - | - | - | - | - | - | 28.95 |
| 462 | 23.11.10 | Male | 6 | - | - | - | 29.87 | - | - | - | - | - | - | - | - | - | - | - | - | - | - | - | 30.82 |
| 463 | 23.11.13 | Female | 9 | 27.13 | - | - | 28.99 | - | - | - | - | - | - | - | - | - | - | - | - | - | - | 36.03 | 29.74 |
| 464 | 23.11.13 | Male | 43 | - | - | - | 31.12 | - | - | - | - | - | - | - | - | - | - | - | - | - | - | - | 30.96 |
| 465 | 23.11.13 | Male | 7 | - | - | - | 30.43 | - | - | - | - | - | - | - | - | - | - | - | - | - | - | - | 29.69 |
| 466 | 23.11.13 | Male | 4 | - | - | - | 30.7 | - | - | - | - | 25.07 | - | - | - | - | - | - | - | - | - | - | 32.15 |
| 467 | 23.11.13 | Male | 10 | 33.57 | - | - | 32.97 | - | - | - | - | - | - | - | - | - | - | - | - | - | - | 37.59 | 33.08 |
| 468 | 23.11.13 | Female | 5 | - | - | - | 31.84 | - | - | - | - | - | - | - | - | 29.85 | - | - | - | - | - | - | 33.72 |
| 469 | 23.11.13 | Female | 5 | - | - | - | 32.71 | - | - | - | 30.94 | - | - | 26.45 | - | - | - | - | - | - | - | - | 34.41 |
| 470 | 23.11.13 | Male | 8 | - | - | - | 30.15 | - | - | - | - | - | - | 26.73 | - | - | - | - | - | - | - | - | 30.75 |
| 471 | 23.11.13 | Male | 8 | - | - | - | 30.04 | - | - | - | - | - | - | - | - | - | - | - | - | - | - | - | 32.06 |
| 472 | 23.11.13 | Female | 8 | - | - | - | 30.5 | - | - | - | - | - | - | - | - | - | - | - | - | - | - | - | 30.84 |
| 473 | 23.11.13 | Female | 35 | 32.87 | - | - | 30.68 | - | - | - | - | - | - | - | - | - | - | - | - | - | - | - | 31.09 |
| 474 | 23.11.13 | Female | 13 | - | - | 34.75 | 33.04 | - | - | - | - | - | - | - | - | - | - | - | - | - | 38.26 | - | 33.7 |
| 475 | 23.11.13 | Male | 1 | - | - | - | 30.43 | - | - | - | - | - | - | - | - | - | - | - | - | - | - | - | 31.77 |
| 476 | 23.11.13 | Female | 7 | - | 28.27 | - | 31.67 | - | - | - | - | - | - | 32.23 | - | - | - | - | - | - | - | - | 32.55 |
| 477 | 23.11.13 | Female | 33 | - | - | - | 29.43 | - | - | - | - | - | - | - | - | - | - | - | - | - | - | - | 28.63 |
| 478 | 23.11.13 | Male | 6 | - | - | - | 31.71 | - | - | - | - | - | - | - | - | - | - | - | - | - | - | - | 32.3 |
| 479 | 23.11.13 | Male | 4 | - | 29.74 | - | 30.83 | - | - | - | - | - | - | - | - | - | - | - | - | - | - | - | 30.75 |
| 480 | 23.11.13 | Male | 5 | 34.06 | - | - | 32.94 | - | - | - | - | - | - | - | - | - | - | - | - | - | - | 35.52 | 30.01 |
| 481 | 23.11.13 | Male | 8 | - | - | - | 32.87 | - | - | - | - | - | - | 30.88 | - | - | - | - | - | - | - | - | 29.09 |
| 482 | 23.11.13 | Male | 6 | - | - | - | 29.47 | - | - | - | - | - | - | - | - | 30.63 | - | - | - | - | - | - | 30.29 |
| 483 | 23.11.14 | Male | 6 | - | - | - | 30.58 | - | - | - | - | - | 29.58 | - | - | - | - | - | - | - | - | - | 31.66 |
| 484 | 23.11.14 | Female | 7 | - | - | - | 32.01 | - | - | - | - | - | - | 29.12 | - | - | - | - | - | - | - | - | 32.77 |
| 485 | 23.11.14 | Male | 9 | - | - | - | 29.86 | - | - | - | - | - | - | - | - | - | - | - | - | - | - | - | 29.37 |
| 486 | 23.11.14 | Male | 1 | - | - | - | 25.27 | - | - | - | - | - | - | - | - | - | - | - | - | - | - | - | 31.22 |
| 487 | 23.11.14 | Male | 4 | - | 26.8 | - | 29.03 | - | - | - | - | - | - | - | - | - | - | - | - | - | - | - | 30.59 |
| 488 | 23.11.14 | Male | 4 | - | - | - | 31.15 | - | - | - | - | - | - | - | - | - | - | - | - | - | - | - | 32.63 |
| 489 | 23.11.14 | Male | 13 | 32.54 | - | - | 33.79 | - | 35.65 | - | - | - | - | - | - | - | - | - | - | - | - | 34.16 | 34.65 |
| 490 | 23.11.14 | Female | 5 | - | - | - | 28.91 | - | - | - | - | - | - | 25.58 | - | - | - | - | - | - | - | - | 30.79 |
| 491 | 23.11.14 | Female | 35 | - | - | - | 30.67 | - | - | - | - | - | - | - | - | - | - | - | - | - | - | - | 32.22 |
| 492 | 23.11.15 | Male | 12 | 24.71 | - | - | 30.13 | - | - | - | - | - | 27.97 | - | - | - | - | - | - | - | - | 25.6 | 31.72 |
| 493 | 23.11.15 | Male | 8 | - | - | - | 32.88 | - | - | 29.97 | - | 28.31 | - | - | - | - | - | - | - | - | - | - | 35.78 |
| 494 | 23.11.15 | Male | 3 | - | - | - | 31.01 | - | - | - | - | - | - | - | - | - | - | - | - | - | - | - | 35.55 |
| 495 | 23.11.15 | Female | 5 | - | - | - | 31.4 | - | - | - | - | - | - | - | - | - | - | - | - | - | - | - | 34.02 |
| 496 | 23.11.15 | Female | 11 | 28.93 | - | - | 30.66 | - | - | - | - | - | - | - | - | - | - | - | - | - | - | 29.46 | 31.43 |
| 497 | 23.11.15 | Male | 17 | - | - | - | 34.73 | - | - | - | - | - | - | - | - | - | - | - | - | - | - | - | 34.84 |
| 498 | 23.11.15 | Male | 15 | 32.57 | - | - | 33.05 | - | - | - | - | - | - | - | - | - | - | - | 31.9 | - | - | 33.5 | 33.9 |
| 499 | 23.11.15 | Female | 17 | - | - | - | 35.01 | - | - | - | - | - | - | - | - | - | - | - | - | - | - | - | 36.44 |
| 500 | 23.11.15 | Male | 6 | 24.28 | - | - | 32.04 | - | - | - | - | - | - | - | - | - | - | - | - | - | - | 25.89 | 32.18 |
| 501 | 23.11.15 | Female | 29 | - | - | - | 34.64 | - | - | - | - | - | - | - | - | - | - | - | - | - | - | - | 34.81 |
| 502 | 23.11.15 | Female | 46 | 31.67 | - | - | 31.96 | - | - | - | - | - | - | - | - | - | - | - | - | - | - | 32.08 | 32.24 |
| 503 | 23.11.15 | Female | 31 | - | - | - | 32.08 | - | - | - | - | - | - | - | - | - | - | - | - | - | - | - | 33.81 |
| 504 | 23.11.15 | Female | 4 | - | - | - | 31.72 | - | - | - | - | - | - | - | - | - | - | - | - | - | - | - | 33.23 |
| 505 | 23.11.15 | Male | 41 | 32.97 | - | - | 32.16 | - | - | - | - | - | - | - | - | - | - | - | - | - | - | - | 30.98 |
| 506 | 23.11.15 | Male | 16 | 32 | - | - | 32.54 | - | - | - | - | - | - | - | - | - | - | - | - | - | - | 32.81 | 32.82 |
| 507 | 23.11.15 | Female | 17 | - | - | - | 33.07 | - | - | - | - | - | - | - | - | 32.45 | - | - | - | - | - | - | 34.44 |
| 508 | 23.11.15 | Female | 68 | - | - | - | 28.1 | - | - | - | - | - | - | - | - | - | - | - | - | - | - | - | 28.15 |
| 509 | 23.11.15 | Male | 28 | - | - | - | 32.61 | - | - | - | - | - | - | - | - | - | - | - | - | - | - | - | 32.67 |
| 510 | 23.11.15 | Female | 4 | - | - | - | 31.77 | - | - | - | - | - | - | - | - | - | - | - | - | - | - | - | 34.55 |
| 511 | 23.11.15 | Female | 3 | - | - | - | 29.62 | - | - | - | - | - | - | - | - | - | - | - | - | - | - | - | 31.07 |
| 512 | 23.11.15 | Female | 50 | - | - | - | 27.94 | - | - | - | - | - | - | - | - | - | - | - | - | - | - | - | 27.85 |
| 513 | 23.11.15 | Female | 26 | - | - | - | 31.77 | - | - | - | - | - | - | - | - | - | - | - | - | - | - | - | 32.91 |
| 514 | 23.11.15 | Female | 8 | 33.63 | - | - | 32.19 | - | - | - | - | - | - | - | - | - | - | - | - | - | - | - | 32.78 |
| 515 | 23.11.15 | Male | 9 | - | - | - | 31.53 | - | - | - | 29.52 | - | 31.74 | - | - | - | - | - | - | - | - | - | 32.12 |
| 516 | 23.11.15 | Female | 8 | - | - | - | 30.49 | - | - | - | - | - | - | - | - | - | - | - | - | - | - | - | 31.19 |
| 517 | 23.11.15 | Female | 45 | - | - | - | 29.72 | - | - | - | - | - | - | - | - | - | - | - | - | - | - | - | 31.53 |
| 518 | 23.11.16 | Male | 31 | - | 28.93 | - | 29.5 | - | - | - | - | - | - | - | - | - | - | - | - | - | - | - | 29.98 |
| 519 | 23.11.16 | Female | 33 | - | - | - | 30.03 | - | - | - | - | - | - | - | - | - | - | - | - | - | - | - | 30.69 |
| 520 | 23.11.16 | Female | 29 | - | - | - | 32.24 | - | - | - | - | 28.37 | - | - | - | - | - | - | - | - | - | - | 32.53 |
| 521 | 23.11.16 | Male | 5 | - | - | - | 30.93 | - | - | - | 29.48 | - | - | - | - | - | - | - | - | - | - | - | 31.02 |
| 522 | 23.11.16 | Male | 7 | - | - | - | 30.77 | - | - | - | - | - | - | - | - | - | - | - | - | - | - | - | 31.38 |
| 523 | 23.11.16 | Female | 7 | - | - | - | 29.18 | - | - | - | - | - | - | - | - | - | - | - | - | - | - | - | 29.25 |
| 524 | 23.11.16 | Male | 9 | - | - | - | 30.48 | - | - | - | - | - | - | - | - | - | - | - | - | - | - | - | 30.15 |
| 525 | 23.11.17 | Male | 8 | - | - | - | 34.29 | - | - | - | - | - | - | 31.15 | - | - | - | - | - | - | - | - | 34.13 |
| 526 | 23.11.17 | Female | 7 | 30.18 | - | - | 30.54 | - | - | - | - | - | - | - | - | - | - | - | - | - | - | 31.27 | 30.97 |
| 527 | 23.11.17 | Female | 16 | - | - | - | 30.55 | - | - | - | - | - | - | - | - | - | - | - | - | - | - | - | 34.59 |
| 528 | 23.11.17 | Female | 6 | - | - | - | 30.4 | - | - | - | - | - | - | - | - | - | - | - | - | - | - | - | 30.22 |
| 529 | 23.11.17 | Female | 7 | - | - | - | 31.93 | - | - | - | - | - | - | - | - | - | - | - | - | - | - | - | 31.98 |
| 530 | 23.11.20 | Female | 5 | - | - | - | 33.05 | - | - | - | - | - | - | - | - | - | - | - | - | - | - | - | 34.12 |
| 531 | 23.11.20 | Female | 9 | 25.47 | - | - | 26.92 | - | - | - | - | - | - | - | - | - | - | - | - | - | - | 25.93 | 28.59 |
| 532 | 23.11.20 | Male | 9 | 29.13 | - | - | 29.9 | - | - | - | 22 | - | - | - | - | - | - | - | - | - | - | 31.26 | 30.88 |
| 533 | 23.11.20 | Male | 3 | - | - | - | 30.8 | - | - | - | - | - | 32.13 | - | - | - | - | - | - | - | - | - | 31.16 |
| 534 | 23.11.20 | Female | 5 | - | - | - | 30.06 | - | 28.15 | - | - | 28.15 | - | - | - | - | - | - | - | - | - | - | 31.14 |
| 535 | 23.11.20 | Male | 5 | - | - | - | 28.46 | - | - | - | - | - | - | - | - | - | - | - | - | - | - | - | 29.37 |
| 536 | 23.11.20 | Male | 4 | - | - | - | 27.82 | - | - | - | - | - | - | - | - | - | - | - | - | - | - | - | 28.19 |
| 537 | 23.11.20 | Female | 7 | - | - | - | 33.15 | - | - | - | - | - | - | - | - | - | - | - | - | - | - | - | 33.22 |
| 538 | 23.11.20 | Male | 6 | - | - | - | 30.69 | - | - | - | - | - | - | - | - | - | - | - | - | - | - | - | 32.15 |
| 539 | 23.11.20 | Female | 9 | - | - | - | 33.31 | - | - | - | - | - | - | - | - | - | - | - | - | - | - | - | 35.18 |
| 540 | 23.11.20 | Female | 39 | - | - | - | 32.6 | - | - | - | - | - | - | - | - | - | - | - | 32.99 | - | - | - | 32.79 |
| 541 | 23.11.20 | Male | 11 | - | - | - | 32.53 | - | - | - | - | - | - | - | - | - | - | - | - | - | - | - | 33.17 |
| 542 | 23.11.20 | Female | 9 | - | - | - | 28.85 | - | - | - | - | - | - | - | - | - | - | - | - | - | - | - | 30.01 |
| 543 | 23.11.20 | Male | 31 | - | - | - | 30.94 | - | - | - | - | - | - | 30.01 | - | - | - | - | - | - | - | - | 31.77 |
| 544 | 23.11.20 | Female | 4 | - | - | - | 31.67 | - | - | - | - | - | - | 28.99 | - | - | - | - | - | - | - | - | 32.41 |
| 545 | 23.11.20 | Female | 68 | - | - | - | 31.25 | - | - | - | - | - | - | - | - | - | - | - | - | - | - | - | 30.7 |
| 546 | 23.11.20 | Male | 11 | 30.53 | - | - | 31.06 | - | - | - | - | - | - | 29.78 | - | - | - | - | - | - | - | 32.78 | 31.74 |
| 547 | 23.11.20 | Female | 43 | - | - | - | 33.68 | - | - | - | - | - | - | - | - | - | - | - | - | - | - | - | 33.33 |
| 548 | 23.11.20 | Male | 8 | 29.16 | - | - | 30.29 | - | - | - | - | 28.16 | - | 29.93 | - | - | - | - | - | - | - | 34.06 | 29.78 |
| 549 | 23.11.20 | Female | 5 | 33.29 | - | - | 30.74 | - | - | - | - | - | - | - | - | - | - | - | - | - | - | 34.03 | 30.35 |
| 550 | 23.11.20 | Female | 6 | - | - | - | 28.67 | - | - | - | - | - | - | - | - | - | - | - | - | - | - | - | 29.58 |
| 551 | 23.11.20 | Male | 12 | - | - | - | 32.48 | - | - | 29.98 | - | 30.01 | - | - | - | - | - | - | - | - | - | - | 31.12 |
| 552 | 23.11.20 | Male | 9 | - | - | - | 32.55 | - | - | - | - | - | - | - | - | - | - | - | - | - | - | - | 33.16 |
| 553 | 23.11.20 | Female | 31 | - | - | - | 31.54 | - | - | - | - | - | - | - | - | - | - | - | - | - | - | - | 31.62 |
| 554 | 23.11.20 | Female | 5 | - | - | - | 32.2 | - | - | - | - | - | - | 30.03 | - | - | - | - | - | - | - | - | 32.39 |
| 555 | 23.11.20 | Female | 8 | 31.62 | - | - | 32.75 | - | - | - | - | - | - | - | - | - | - | - | - | - | - | 32.65 | 33.21 |
| 556 | 23.11.20 | Female | 4 | - | - | - | 32.33 | - | - | 27.1 | - | 29.22 | - | - | - | - | - | - | 32.98 | - | - | - | 33.34 |
| 557 | 23.11.20 | Male | 13 | 30.46 | - | - | 31.71 | - | - | - | - | - | - | - | - | - | - | - | - | - | - | 29.79 | 33.09 |
| 558 | 23.11.20 | Female | 9 | 34.75 | - | - | 33.26 | - | - | - | - | - | - | - | - | - | - | - | - | - | - | 36 | 33.84 |
| 559 | 23.11.20 | Male | 10 | 31.05 | - | - | 32.56 | - | - | - | - | - | - | - | - | - | - | - | - | - | - | 33.6 | 32.46 |
| 560 | 23.11.20 | Female | 10 | - | - | - | 29.07 | - | - | - | - | - | - | 26.7 | - | - | - | - | - | - | - | - | 29.78 |
| 561 | 23.11.20 | Male | 7 | 26.9 | - | - | 26.12 | - | - | - | - | - | - | - | - | - | - | - | - | - | - | 27.25 | 27.85 |
| 562 | 23.11.20 | Male | 17 | - | - | - | 30.75 | - | - | - | - | - | - | - | - | - | - | - | - | - | - | - | 31.47 |
| 563 | 23.11.20 | Male | 7 | 31.7 | - | - | 33.11 | - | - | - | - | - | - | 34.64 | - | - | - | - | - | - | - | 32.15 | 31.74 |
| 564 | 23.11.20 | Male | 12 | - | - | - | 31.9 | - | - | - | - | - | - | - | - | - | - | - | - | - | - | - | 33.2 |
| 565 | 23.11.20 | Male | 3 | - | 28.27 | - | 29.12 | - | - | 24.6 | - | 25.76 | - | - | - | - | - | - | - | - | - | - | 30.62 |
| 566 | 23.11.20 | Male | 7 | - | - | - | 26.89 | - | - | - | - | - | - | - | - | - | - | - | - | - | - | - | 28.08 |
| 567 | 23.11.20 | Female | 6 | - | - | - | 29.99 | - | - | - | - | - | - | 28.81 | - | - | - | - | - | - | - | - | 29.95 |
| 568 | 23.11.20 | Female | 19 | - | - | - | 31.68 | - | - | - | - | - | - | - | - | - | - | - | - | - | - | - | 33.31 |
| 569 | 23.11.22 | Male | 15 | - | - | - | 31.16 | - | - | - | - | - | - | - | - | - | - | - | - | - | - | - | 31.72 |
| 570 | 23.11.22 | Male | 11 | - | - | - | 32.62 | - | - | - | - | - | - | - | - | - | - | - | - | - | - | - | 32.65 |
| 571 | 23.11.22 | Male | 37 | - | - | - | 32.71 | - | - | - | - | - | - | - | - | - | - | - | - | - | - | - | 32.15 |
| 572 | 23.11.22 | Male | 5 | - | - | - | 29.67 | - | - | - | - | - | - | - | - | - | - | - | - | - | - | - | 30.68 |
| 573 | 23.11.22 | Female | 8 | - | - | - | 32.95 | - | - | 29.43 | - | - | - | - | - | - | - | - | - | - | - | - | 35.26 |
| 574 | 23.11.22 | Male | 3 | - | - | - | 33.46 | - | - | - | - | - | - | - | - | - | - | - | - | - | - | - | 34.25 |
| 575 | 23.11.22 | Female | 5 | - | - | - | 31.46 | - | - | - | - | - | - | - | - | - | - | - | - | - | - | - | 31.08 |
| 576 | 23.11.22 | Female | 8 | - | - | - | 30.27 | - | - | - | - | - | - | - | - | - | - | - | - | - | - | - | 35.19 |
| 577 | 23.11.22 | Male | 7 | 30.85 | - | - | 31.45 | - | - | - | - | - | - | - | - | - | - | - | - | - | - | 32.56 | 33.98 |
| 578 | 23.11.22 | Male | 5 | 29.05 | - | - | 28.51 | - | - | - | - | - | - | - | - | - | - | - | - | - | - | 33.76 | 28.85 |
| 579 | 23.11.22 | Female | 13 | - | - | - | 28.02 | - | - | - | - | - | - | - | - | - | - | - | - | - | - | - | 27.79 |
| 580 | 23.11.22 | Male | 6 | 33.22 | - | - | 29.98 | - | - | - | - | - | - | - | - | - | - | - | - | - | - | 33.31 | 30.41 |
| 581 | 23.11.22 | Female | 6 | - | - | - | 30.61 | - | - | - | - | - | - | - | - | - | - | - | - | - | - | - | 32.02 |
| 582 | 23.11.22 | Female | 6 | - | - | - | 31.15 | - | - | - | - | - | - | - | - | - | - | - | - | - | - | - | 33.5 |
| 583 | 23.11.22 | Female | 26 | - | - | - | 32.96 | - | - | - | - | - | - | 31.59 | - | - | - | - | - | - | - | - | 33.66 |
| 584 | 23.11.22 | Female | 8 | 31.61 | - | - | 32.91 | - | - | - | - | - | - | - | - | - | - | - | - | - | - | 32.45 | 33.18 |
| 585 | 23.11.22 | Male | 16 | 32.01 | - | - | 32.86 | - | - | - | - | - | - | 35.11 | - | - | - | - | - | - | - | 31.85 | 34.21 |
| 586 | 23.11.22 | Male | 6 | - | 30.15 | - | 32.45 | - | - | - | - | - | - | - | - | - | - | - | - | - | - | - | 32.26 |
| 587 | 23.11.22 | Female | 24 | - | - | - | 30.94 | - | - | - | - | - | - | - | - | - | - | - | - | - | - | - | 30.25 |
| 588 | 23.11.22 | Female | 25 | - | - | - | 31.18 | - | - | - | - | - | - | - | - | - | - | - | - | - | - | - | 32.65 |
| 589 | 23.11.22 | Male | 10 | 30.98 | - | - | 31.18 | - | - | - | - | - | - | - | - | - | - | - | - | - | - | 31.03 | 32.69 |
| 590 | 23.11.23 | Female | 8 | - | - | - | 29.63 | - | - | 24.6 | - | 24.96 | 30.26 | - | - | - | - | - | - | - | - | - | 30.1 |
| 591 | 23.11.23 | Female | 14 | 32.32 | - | - | 32.82 | - | - | - | - | - | - | 32.07 | - | - | - | - | - | - | - | 34.27 | 32.69 |
| 592 | 23.11.23 | Female | 38 | - | - | - | 27.42 | - | - | - | - | - | - | - | - | - | - | - | - | - | - | - | 27.66 |
| 593 | 23.11.23 | Male | 10 | 31.51 | - | - | 30.11 | - | - | - | - | - | - | 38.82 | - | - | - | - | - | - | - | 38.73 | 32.09 |
| 594 | 23.11.23 | Female | 3 | - | - | - | 29.48 | - | - | - | - | - | - | 33.34 | - | - | - | - | - | - | - | - | 29.74 |
| 595 | 23.11.23 | Female | 3 | - | - | - | 30.73 | - | - | - | - | - | - | - | - | - | - | - | - | - | - | - | 31.99 |
| 596 | 23.11.23 | Male | 34 | - | - | 31.72 | 31.95 | - | - | - | - | - | - | - | - | - | - | - | - | - | 32.89 | - | 32.08 |
| 597 | 23.11.23 | Male | 16 | - | - | - | 31.58 | - | - | - | - | - | - | - | - | - | - | - | 29.27 | - | - | - | 31.31 |
| 598 | 23.11.23 | Female | 6 | - | - | - | 30.33 | - | - | - | - | - | - | - | - | - | - | - | - | - | - | - | 32.85 |
| 599 | 23.11.23 | Male | 25 | - | - | - | 30.68 | - | - | - | - | - | - | - | - | - | - | - | - | - | - | - | 31.16 |
| 600 | 23.11.23 | Male | 6 | - | - | - | 28.81 | - | - | - | - | - | - | - | - | - | - | - | - | - | - | - | 29.2 |
| 601 | 23.11.23 | Male | 4 | - | 32.01 | - | 31.59 | - | - | - | - | - | - | - | - | - | - | - | - | - | - | - | 31.17 |
| 602 | 23.11.23 | Female | 5 | - | - | - | 30.58 | - | - | - | - | - | - | - | - | - | - | - | 31.18 | - | - | - | 30.46 |
| 603 | 23.11.24 | Female | 8 | - | - | - | 28.5 | - | - | - | 32.05 | - | - | - | - | - | - | - | - | - | - | - | 28.62 |
| 604 | 23.11.24 | Male | 16 | - | - | - | 32.09 | - | - | - | - | - | - | - | - | - | - | - | - | - | - | - | 31.61 |
| 605 | 23.11.24 | Male | 35 | - | - | - | 27.79 | - | - | - | - | - | - | - | - | - | - | - | - | - | - | - | 27.52 |
| 606 | 23.11.24 | Female | 36 | - | - | - | 26.78 | - | - | - | - | - | - | - | - | - | - | - | - | - | - | - | 27.24 |
| 607 | 23.11.24 | Male | 10 | 31.15 | - | - | 29.02 | - | - | - | - | - | - | - | - | - | - | - | - | - | - | 34.89 | 29.94 |
| 608 | 23.11.24 | Male | 6 | - | - | - | 30.05 | - | - | - | - | - | - | - | - | - | - | - | - | - | - | - | 29.63 |
| 609 | 23.11.24 | Male | 22 | - | - | 32.79 | 30.95 | - | - | - | - | - | - | - | - | - | - | - | - | - | 34.51 | - | 30.72 |
| 610 | 23.11.24 | Male | 1 | - | - | - | 27.42 | - | - | - | - | - | - | - | - | - | - | - | - | - | - | - | 29.14 |
| 611 | 23.11.24 | Male | 31 | 29.54 | - | - | 27.86 | - | - | - | - | - | - | - | - | - | - | - | - | - | - | 34.34 | 28.22 |
| 612 | 23.11.24 | Female | 10 | - | - | - | 30.92 | - | - | - | - | - | - | 27.47 | - | - | - | - | - | - | - | - | 31.2 |
| 613 | 23.11.24 | Male | 3 | - | - | - | 28.8 | - | - | - | - | - | - | - | - | - | - | - | - | - | - | - | 28.5 |
| 614 | 23.11.24 | Male | 20 | - | - | - | 28.93 | - | - | - | - | - | - | - | - | - | - | - | 31.39 | - | - | - | 30.09 |
| 615 | 23.11.24 | Female | 44 | 29.07 | - | - | 27.17 | - | - | - | - | - | - | - | - | - | - | - | - | - | - | 31.02 | 28.71 |
| 616 | 23.11.27 | Male | 33 | - | - | - | 27.45 | - | - | - | - | - | - | 28.23 | - | - | - | - | - | - | - | - | 27.16 |
| 617 | 23.11.27 | Female | 37 | - | - | - | 29.84 | - | - | 29.42 | - | 29.81 | - | - | - | - | - | - | - | - | - | - | 29.29 |
| 618 | 23.11.27 | Male | 16 | - | - | - | 31.06 | - | - | - | - | - | - | - | - | - | - | - | - | - | - | - | 31.17 |
| 619 | 23.11.27 | Female | 69 | 29.71 | - | - | 26.44 | - | - | - | - | - | - | - | - | - | - | - | - | - | - | 33.06 | 26.69 |
| 620 | 23.11.27 | Female | 29 | - | - | - | 31.05 | - | - | - | - | - | - | - | - | - | - | - | - | - | - | - | 33.58 |
| 621 | 23.11.27 | Male | 67 | - | - | - | 28.67 | - | - | - | - | - | - | - | - | - | - | - | - | - | - | - | 27.36 |
| 622 | 23.11.27 | Female | 5 | - | - | - | 28.59 | - | - | - | - | 24.13 | - | 23.93 | - | - | - | - | - | - | - | - | 29.23 |
| 623 | 23.11.27 | Female | 25 | - | - | - | 27.04 | - | - | - | - | - | - | 28.22 | - | - | - | - | - | - | - | - | 27.14 |
| 624 | 23.11.27 | Male | 11 | - | - | - | 29.64 | - | - | 27.48 | - | 28.6 | - | 28.06 | - | - | - | - | - | - | - | - | 29.33 |
| 625 | 23.11.27 | Female | 42 | - | - | 27.92 | 31.17 | - | - | - | - | - | - | - | - | - | - | - | - | - | 27.19 | - | 31.07 |
| 626 | 23.11.27 | Female | 8 | - | - | - | 28.96 | - | - | - | - | - | - | - | - | - | - | - | - | - | - | - | 29.11 |
| 627 | 23.11.27 | Female | 17 | - | - | - | 30.93 | - | - | - | - | - | - | - | - | - | - | - | - | - | - | - | 30.68 |
| 628 | 23.11.27 | Female | 28 | - | - | - | 30.23 | - | - | - | - | - | - | - | - | - | - | - | - | - | - | - | 30.1 |
| 629 | 23.11.27 | Female | 13 | - | - | - | 33.43 | - | - | - | - | 29.22 | - | - | - | - | - | - | - | - | - | - | 33.22 |
| 630 | 23.11.27 | Male | 13 | - | - | - | 31.14 | - | - | - | - | - | - | - | - | - | - | - | - | - | - | - | 31.6 |
| 631 | 23.11.27 | Male | 10 | - | - | - | 27.9 | - | - | 26.75 | - | 26.86 | - | - | - | - | - | - | - | - | - | - | 28.36 |
| 632 | 23.11.27 | Male | 15 | 29.84 | - | - | 29.99 | - | - | - | - | - | - | - | - | - | - | - | - | - | - | 33.36 | 29.81 |
| 633 | 23.11.27 | Female | 6 | - | 30.31 | - | 29.88 | - | - | - | - | - | - | - | - | - | - | - | - | - | - | - | 29.49 |
| 634 | 23.11.27 | Male | 15 | - | - | - | 28.52 | - | - | - | - | - | - | - | - | - | - | - | - | - | - | - | 29.36 |
| 635 | 23.11.27 | Male | 19 | - | - | 29.64 | 28.53 | - | 35.54 | - | - | - | - | - | - | - | - | - | - | - | 33.61 | - | 29.34 |
| 636 | 23.11.27 | Female | 55 | - | - | - | 29.55 | - | - | - | - | - | - | - | - | - | - | - | - | - | - | - | 29.14 |
| 637 | 23.11.27 | Male | 5 | - | - | - | 29.55 | - | - | - | - | - | - | 29.33 | - | 31.65 | - | - | - | - | - | - | 28.75 |
| 638 | 23.11.27 | Female | 36 | - | - | - | 29.58 | - | - | - | - | - | - | - | - | - | - | - | - | - | - | - | 29.94 |
| 639 | 23.11.27 | Male | 11 | - | - | - | 30.75 | - | - | - | - | - | - | - | - | - | - | - | - | - | - | - | 32.14 |
| 640 | 23.11.28 | Male | 31 | - | - | - | 30.06 | - | - | - | - | - | 26.13 | - | - | - | - | - | - | - | - | - | 30.25 |
| 641 | 23.11.28 | Male | 11 | - | - | - | 31.72 | - | - | - | - | - | 33.55 | - | - | - | - | - | - | - | - | - | 31.4 |
| 642 | 23.11.28 | Male | 13 | 36.38 | - | - | 30.09 | - | - | - | - | - | - | - | - | - | - | - | - | - | - | - | 30.72 |
| 643 | 23.11.28 | Male | 22 | - | - | - | 30.12 | - | - | - | - | - | - | - | - | - | - | - | - | - | - | - | 32.74 |
| 644 | 23.11.28 | Female | 5 | - | - | - | 29.59 | - | - | - | - | - | - | 26.7 | - | - | - | - | - | - | - | - | 29.69 |
| 645 | 23.11.28 | Female | 62 | - | - | - | 31.27 | - | - | - | 30.31 | - | - | - | - | - | - | - | - | - | - | - | 30.44 |
| 646 | 23.11.28 | Female | 7 | - | - | - | 29.65 | - | - | - | - | - | - | - | - | - | - | - | - | - | - | - | 31.77 |
| 647 | 23.11.28 | Female | 42 | - | - | - | 29.06 | - | - | - | - | - | 29.32 | - | - | - | - | - | - | - | - | - | 29.12 |
| 648 | 23.11.28 | Male | 6 | 29.49 | - | - | 28.67 | - | - | - | - | - | - | - | - | - | - | - | - | - | - | - | 31.17 |
| 649 | 23.11.28 | Female | 11 | - | - | 31.63 | 31.68 | - | - | - | - | - | - | - | - | - | - | - | - | - | 34.28 | - | 32.81 |
| 650 | 23.11.28 | Male | 31 | - | - | - | 28.81 | - | - | - | - | - | - | - | - | - | - | - | - | - | - | - | 29.13 |
| 651 | 23.11.28 | Female | 77 | - | - | - | 30.9 | - | - | - | - | - | - | - | - | - | - | - | - | - | - | - | 29.8 |
| 652 | 23.11.28 | Female | 4 | - | - | - | 31.99 | - | - | 27.42 | - | 29.72 | - | - | - | - | - | - | - | - | - | - | 32.45 |
| 653 | 23.11.28 | Female | 13 | - | - | - | 31.61 | - | - | - | - | - | - | - | - | - | - | - | - | - | - | - | 31.73 |
| 654 | 23.11.28 | Female | 3 | - | - | - | 28.85 | - | - | 28.81 | - | 31 | - | - | - | - | - | - | - | - | - | - | 28.83 |
| 655 | 23.11.28 | Male | 14 | - | - | - | 31.08 | - | - | - | - | - | - | - | - | - | - | - | - | - | - | - | 30.3 |
| 656 | 23.11.28 | Male | 36 | - | - | - | 29.6 | - | - | - | - | - | - | - | - | - | - | - | - | - | - | - | 30.05 |
| 657 | 23.11.28 | Female | 27 | - | - | - | 30.4 | - | - | - | - | 29.81 | - | - | - | - | - | - | - | - | - | - | 30.29 |
| 658 | 23.11.28 | Male | 10 | 31.68 | - | - | 31.56 | - | - | - | - | - | - | - | - | - | - | - | - | - | - | 38.47 | 32.34 |
| 659 | 23.11.28 | Male | 13 | - | - | - | 28.99 | - | - | - | - | - | - | - | - | - | - | - | - | - | - | - | 28.61 |
| 660 | 23.11.28 | Male | 9 | - | - | - | 28.25 | - | - | - | - | - | - | - | - | - | - | - | - | - | - | - | 29.03 |
| 661 | 23.11.28 | Male | 30 | - | - | - | 25.54 | - | - | - | - | - | - | - | - | - | - | - | 26.82 | - | - | - | 26.62 |
| 662 | 23.11.28 | Male | 13 | - | - | - | 25.42 | - | - | - | - | - | - | - | - | - | - | - | - | - | - | - | 25.49 |
| 663 | 23.11.28 | Female | 60 | - | - | - | 29.93 | - | - | - | - | - | - | - | - | - | - | - | - | - | - | - | 29.72 |
| 664 | 23.11.28 | Female | 48 | - | - | - | 28.09 | - | - | - | - | - | - | - | - | - | - | - | - | - | - | - | 28.66 |
| 665 | 23.11.28 | Female | 24 | - | - | - | 26.43 | - | - | - | - | - | - | - | - | - | - | - | - | - | - | - | 26.25 |
| 666 | 23.11.28 | Female | 14 | - | - | - | 33.16 | - | - | - | - | - | - | - | - | - | - | - | - | - | - | - | 33.57 |
| 667 | 23.11.28 | Male | 19 | - | - | - | 31.56 | - | - | - | - | - | - | - | - | - | - | - | - | - | - | - | 30.83 |
| 668 | 23.11.29 | Female | 40 | - | - | - | 26.83 | - | - | - | - | - | - | - | - | - | - | - | - | - | - | - | 26.97 |
| 669 | 23.11.29 | Male | 7 | 29.82 | - | - | 29.94 | - | - | - | - | - | - | - | - | - | - | - | - | - | - | 30.16 | 30.02 |
| 670 | 23.11.29 | Male | 50 | - | - | - | 30.12 | - | - | - | - | - | - | - | - | - | - | - | - | - | - | - | 30.09 |
| 671 | 23.11.29 | Female | 53 | - | - | - | 30.23 | - | - | - | - | - | - | 27.54 | - | - | - | - | - | - | - | - | 29.4 |
| 672 | 23.11.29 | Male | 5 | - | - | - | 32.54 | - | - | - | - | - | - | - | - | - | - | - | 35.16 | - | - | - | 33.29 |
| 673 | 23.11.29 | Female | 13 | - | - | - | 29.47 | - | - | - | - | - | - | - | - | - | - | - | - | - | - | - | 30.17 |
| 674 | 23.11.29 | Female | 20 | - | - | - | 31.78 | - | - | - | - | - | - | - | - | - | - | - | - | - | - | - | 32.37 |
| 675 | 23.11.29 | Male | 7 | - | - | - | 32.7 | - | - | - | - | - | 32 | - | - | - | - | - | - | - | - | - | 33.49 |
| 676 | 23.11.29 | Male | 6 | - | - | 31.3 | 32.15 | - | - | - | - | - | - | - | - | - | - | - | - | - | 35.2 | - | 33.51 |
| 677 | 23.11.29 | Female | 5 | - | - | - | 31.1 | - | - | - | - | - | - | - | - | - | - | - | - | - | - | - | 32.46 |
| 678 | 23.11.29 | Female | 14 | - | - | - | 29.48 | - | - | - | - | - | - | - | - | - | - | - | - | - | - | - | 29.95 |
| 679 | 23.11.29 | Female | 12 | 26.89 | 27.31 | - | 30.58 | - | - | - | - | - | - | - | - | - | - | - | - | - | - | 27.32 | 29.88 |
| 680 | 23.11.29 | Male | 34 | - | - | - | 28.53 | - | - | - | - | - | - | - | - | - | - | - | - | - | - | - | 28.69 |
| 681 | 23.11.29 | Male | 9 | 29.69 | - | - | 28.81 | - | - | - | - | - | - | 28.64 | - | - | - | - | - | - | - | 35.36 | 29.66 |
| 682 | 23.11.29 | Female | 7 | - | - | - | 30.74 | - | - | - | - | - | - | - | - | - | - | - | - | - | - | - | 31.59 |
| 683 | 23.11.29 | Male | 9 | - | - | - | 33.64 | - | - | - | - | - | - | 34.13 | - | - | - | - | - | - | - | - | 35.37 |
| 684 | 23.11.29 | Female | 14 | 31.93 | - | - | 29.61 | - | - | - | - | - | - | - | - | - | - | - | - | - | - | 34.83 | 30.36 |
| 685 | 23.11.29 | Female | 14 | - | - | - | 30.66 | - | - | - | - | - | - | - | - | - | - | - | - | - | - | - | 31.69 |
| 686 | 23.11.29 | Female | 25 | 30.22 | - | - | 28.8 | - | - | - | - | - | - | - | - | - | - | - | - | - | - | 32.71 | 28.96 |
| 687 | 23.11.29 | Female | 4 | - | - | - | 31.92 | - | - | - | - | - | - | - | - | - | - | - | - | - | - | - | 31.11 |
| 688 | 23.11.29 | Female | 17 | - | - | - | 32.76 | - | - | - | - | - | - | - | - | - | - | - | - | - | - | - | 33.67 |
| 689 | 23.11.29 | Male | 6 | - | - | - | 29.92 | - | - | - | - | - | - | 29.49 | - | - | - | - | - | - | - | - | 30.58 |
| 690 | 23.11.29 | Female | 41 | - | - | - | 30.76 | - | - | - | - | - | - | - | - | - | - | - | - | - | - | - | 30.59 |
| 691 | 23.11.29 | Male | 19 | - | - | - | 32.83 | - | - | - | - | - | - | - | - | - | - | - | - | - | - | - | 32.56 |
| 692 | 23.11.29 | Male | 10 | - | - | - | 31.5 | - | - | - | - | - | - | - | - | - | - | - | - | - | - | - | 31.82 |
| 693 | 23.11.29 | Male | 14 | - | - | - | 31.86 | - | - | - | - | - | - | - | - | - | - | - | - | - | - | - | 32.35 |
| 694 | 23.11.29 | Female | 11 | - | - | - | 30.53 | - | - | - | - | - | - | - | - | - | - | - | - | - | - | - | 30.33 |
| 695 | 23.11.30 | Female | 41 | - | - | 28.71 | 29.16 | - | - | - | - | - | - | - | - | - | - | - | - | - | 30.3 | - | 30.64 |
| 696 | 23.11.30 | Male | 11 | - | - | - | 32.56 | - | - | - | - | - | - | - | - | - | - | - | - | - | - | - | 34.49 |
| 697 | 23.11.30 | Male | 12 | - | - | - | 31.83 | - | - | - | - | - | - | - | - | - | - | - | - | - | - | - | 33.57 |
| 698 | 23.11.30 | Male | 14 | - | - | - | 29.3 | - | - | - | - | - | - | - | - | - | - | - | - | - | - | - | 30.79 |
| 699 | 23.11.30 | Male | 45 | - | - | - | 29.96 | - | - | - | - | - | - | - | - | - | - | - | - | - | - | - | 31.32 |
| 700 | 23.11.30 | Female | 33 | - | - | 32.34 | 32.73 | - | - | - | - | - | - | - | - | - | - | - | - | - | 39.89 | - | 33.12 |
| 701 | 23.11.30 | Female | 8 | - | - | 33.57 | 32.29 | - | - | - | - | - | - | - | - | - | - | - | - | - | 34.17 | - | 31.74 |
| 702 | 23.11.30 | Female | 13 | 33.89 | - | - | 31.08 | - | - | - | - | - | - | - | - | - | - | - | 32.25 | - | - | 37.53 | 32.49 |
| 703 | 23.11.30 | Female | 8 | - | - | - | 31.5 | - | - | - | - | - | - | - | - | - | - | - | - | - | - | - | 31.22 |
| 704 | 23.11.30 | Female | 26 | - | - | - | 30.56 | - | - | - | - | 30.05 | - | - | - | - | - | - | - | - | - | - | 30.1 |
| 705 | 23.11.30 | Female | 60 | - | - | - | 27.4 | - | - | - | - | - | - | - | - | - | - | - | - | - | - | - | 27.25 |
| 706 | 23.11.30 | Male | 13 | - | - | 28.97 | 29.34 | - | - | - | - | - | - | - | - | - | - | - | - | - | 32.6 | - | 30.89 |
| 707 | 23.11.30 | Female | 15 | - | - | - | 29.69 | - | - | - | - | - | - | - | - | - | - | - | - | - | - | - | 30.94 |
| 708 | 23.11.30 | Male | 29 | - | - | - | 30.24 | - | - | - | - | - | - | - | - | - | - | - | - | - | - | - | 32.54 |
| 709 | 23.11.30 | Male | 5 | - | - | - | 30.5 | - | - | - | - | - | - | - | - | - | - | - | - | - | - | - | 32.02 |
| 710 | 23.11.30 | Male | 17 | - | - | - | 28.8 | - | - | - | - | - | - | - | - | - | - | - | - | - | - | - | 29.81 |
| 711 | 23.11.30 | Female | 10 | - | - | - | 29.67 | - | - | - | - | - | 29.77 | - | - | - | - | - | - | - | - | - | 30.29 |
| 712 | 23.11.30 | Male | 8 | - | - | - | 30.03 | - | - | - | - | - | - | - | - | - | - | - | - | - | - | - | 31.27 |
| 713 | 23.11.30 | Male | 8 | - | - | - | 30.51 | - | - | - | - | - | - | - | - | - | - | - | - | - | - | - | 30.04 |
| 714 | 23.11.30 | Male | 2 | - | - | - | 30.06 | - | - | - | - | - | - | - | - | - | - | - | - | - | - | - | 31.35 |
| 715 | 23.12.01 | Female | 8 | - | - | - | 30.22 | - | - | - | - | - | - | - | - | - | - | - | - | - | - | - | 31.61 |
| 716 | 23.12.01 | Male | 16 | - | - | - | 29.88 | - | - | - | 27.72 | - | - | - | - | - | - | - | - | - | - | - | 30.31 |
| 717 | 23.12.01 | Male | 13 | - | - | - | 30.8 | - | - | - | - | - | - | - | - | - | - | - | - | - | - | - | 30.07 |
| 718 | 23.12.01 | Female | 31 | - | - | - | 30.75 | - | - | - | - | - | - | 28.87 | - | - | - | - | - | - | - | - | 31.45 |
| 719 | 23.12.01 | Male | 5 | - | - | 28.68 | 30.98 | - | - | - | - | - | - | - | - | - | - | - | - | - | 29.52 | - | 32.44 |
| 720 | 23.12.01 | Female | 38 | - | - | - | 29.14 | - | - | - | - | - | - | - | - | - | - | - | - | - | - | - | 28.93 |
| 721 | 23.12.01 | Female | 32 | - | - | - | 28.2 | - | - | - | - | 26.96 | - | - | - | - | - | - | - | - | - | - | 28.23 |
| 722 | 23.12.01 | Male | 3 | - | - | - | 29.96 | - | - | - | - | - | - | - | - | - | - | - | - | - | - | - | 31.24 |
| 723 | 23.12.01 | Male | 27 | - | - | - | 28.95 | - | - | 26.64 | - | 26.48 | - | - | - | - | - | - | - | - | - | - | 29.6 |
| 724 | 23.12.01 | Female | 39 | - | - | - | 29.5 | - | - | - | - | - | - | - | - | - | - | - | - | - | - | - | 29.41 |
| 725 | 23.12.01 | Male | 31 | - | - | - | 27.74 | - | - | - | - | - | - | - | - | - | - | - | - | - | - | - | 27.5 |
| 726 | 23.12.01 | Male | 2 | - | - | - | 30.18 | - | - | - | - | - | - | 26.22 | - | - | - | - | - | - | - | - | 32.7 |
| 727 | 23.12.04 | Male | 6 | - | - | - | 30.03 | - | - | - | - | - | - | 27 | - | - | - | - | - | - | - | - | 31.93 |
| 728 | 23.12.04 | Female | 4 | - | - | - | 27.87 | - | - | - | - | - | - | - | - | - | - | - | - | - | - | - | 28.11 |
| 729 | 23.12.04 | Male | 4 | - | - | - | 32 | - | - | - | - | - | - | 27.6 | - | - | - | - | - | - | - | - | 32.69 |
| 730 | 23.12.04 | Male | 33 | 33.42 | - | - | 29.79 | - | - | - | - | - | - | - | - | - | - | - | - | - | - | 35.86 | 30.43 |
| 731 | 23.12.04 | Female | 9 | - | - | - | 30.04 | - | - | - | - | - | - | 28.7 | - | - | - | - | - | - | - | - | 30.39 |
| 732 | 23.12.04 | Male | 2 | - | - | - | 32.14 | - | - | - | - | - | - | - | - | - | - | - | - | - | - | - | 32.75 |
| 733 | 23.12.04 | Male | 6 | - | - | - | 27.98 | - | - | - | - | - | - | 27.82 | - | - | - | - | - | - | - | - | 27.63 |
| 734 | 23.12.04 | Female | 5 | - | - | - | 26.11 | - | 24.26 | - | - | - | - | - | - | - | - | - | - | - | - | - | 26.77 |
| 735 | 23.12.04 | Male | 4 | - | - | - | 29.58 | - | - | - | - | - | - | - | - | - | - | - | - | - | - | - | 30.9 |
| 736 | 23.12.04 | Female | 47 | - | - | - | 30.73 | - | - | - | - | - | - | - | - | - | - | - | - | - | - | - | 30.94 |
| 737 | 23.12.04 | Male | 5 | - | - | - | 30.86 | - | - | - | - | - | 25.17 | - | - | - | - | - | - | - | - | - | 31.48 |
| 738 | 23.12.04 | Male | 10 | - | - | 30 | 29.02 | - | - | - | - | - | - | - | - | - | - | - | - | - | 34.73 | - | 30.19 |
| 739 | 23.12.04 | Female | 6 | - | - | - | 29.4 | - | 28.71 | - | - | - | 30.78 | 30.96 | - | - | - | - | 28.64 | - | - | - | 29.42 |
| 740 | 23.12.05 | Male | 7 | - | - | - | 30.61 | - | - | - | - | - | 30.87 | - | - | - | - | - | - | - | - | - | 30.28 |
| 741 | 23.12.05 | Male | 13 | - | - | - | 32.16 | - | - | - | - | - | - | 34.17 | - | - | - | - | - | - | - | - | 32.37 |
| 742 | 23.12.05 | Male | 46 | - | - | - | 29.85 | - | - | - | - | - | - | 30.14 | - | - | - | - | - | - | - | - | 29.69 |
| 743 | 23.12.05 | Male | 34 | - | - | 30.78 | 32.81 | - | - | - | - | - | - | 32.21 | - | - | - | - | - | - | 31.85 | - | 33.9 |
| 744 | 23.12.05 | Male | 5 | - | - | - | 32.34 | - | - | - | - | - | - | - | - | - | - | - | - | - | - | - | 33.05 |
| 745 | 23.12.05 | Male | 9 | - | - | 30.13 | 31.03 | - | - | - | - | - | - | - | - | - | - | - | - | - | 36.75 | - | 31.71 |
| 746 | 23.12.05 | Female | 21 | - | - | - | 31.62 | - | - | - | - | - | - | - | - | - | - | - | - | - | - | - | 34.01 |
| 747 | 23.12.05 | Male | 7 | - | - | - | 31.45 | - | - | - | - | - | - | 33.13 | - | - | - | - | - | - | - | - | 30.79 |
| 748 | 23.12.05 | Male | 44 | - | - | - | 28.67 | - | - | - | - | - | - | - | - | - | - | - | - | - | - | - | 28.57 |
| 749 | 23.12.05 | Female | 16 | 27.96 | - | - | 28.46 | - | - | - | - | - | - | - | - | - | - | - | - | - | - | 29.08 | 30.71 |
| 750 | 23.12.05 | Female | 42 | - | - | 30.72 | 31.01 | - | - | - | - | - | - | - | - | - | - | - | - | - | 33.61 | - | 31.63 |
| 751 | 23.12.05 | Male | 20 | - | - | - | 28.96 | - | - | - | - | - | - | - | - | - | - | - | - | - | - | - | 29.78 |
| 752 | 23.12.05 | Female | 7 | - | - | - | 30.04 | - | - | - | - | - | - | - | - | - | - | - | - | - | - | - | 30.01 |
| 753 | 23.12.05 | Female | 7 | - | - | - | 30.7 | - | - | - | - | - | - | - | - | - | - | - | - | - | - | - | 30.78 |
| 754 | 23.12.05 | Female | 25 | - | - | - | 27.59 | - | - | - | - | - | - | 27.14 | - | - | - | - | - | - | - | - | 28.48 |
| 755 | 23.12.05 | Female | 21 | - | - | - | 27.55 | - | - | 28.13 | - | 27.61 | - | - | - | - | - | - | - | - | - | - | 27.85 |
| 756 | 23.12.05 | Female | 53 | - | - | - | 29.81 | - | - | - | - | - | - | - | - | - | - | - | - | - | - | - | 30 |
| 757 | 23.12.05 | Female | 45 | - | - | - | 27.32 | - | - | - | - | - | - | - | - | - | - | - | - | - | - | - | 26.67 |
| 758 | 23.12.05 | Female | 41 | - | - | - | 27.5 | - | - | - | - | - | - | - | - | - | - | - | - | - | - | - | 27.88 |
| 759 | 23.12.05 | Female | 58 | - | - | - | 28.96 | - | - | - | - | - | 27.94 | - | - | - | - | - | - | - | - | - | 30.84 |
| 760 | 23.12.05 | Male | 3 | - | - | - | 27.92 | - | - | - | - | - | - | - | - | - | - | - | - | - | - | - | 28.22 |
| 761 | 23.12.05 | Male | 8 | - | - | 35.31 | 34.15 | - | - | - | - | - | - | - | - | - | - | - | - | - | - | - | 33.22 |
| 762 | 23.12.05 | Female | 8 | - | - | - | 28.94 | - | - | - | 24.59 | - | - | - | - | - | - | - | - | - | - | - | 30.94 |
| 763 | 23.12.05 | Female | 16 | 31.32 | - | - | 30.83 | - | - | - | - | - | - | - | - | - | - | - | - | - | - | 33.84 | 32.07 |
| 764 | 23.12.06 | Female | 11 | - | - | - | 31.62 | - | - | - | - | - | - | - | - | - | - | - | - | - | - | - | 31.22 |
| 765 | 23.12.06 | Female | 38 | - | - | - | 32.61 | - | - | - | - | - | - | - | - | - | - | - | - | - | - | - | 32.44 |
| 766 | 23.12.06 | Male | 39 | - | - | - | 27.9 | - | - | - | - | - | - | - | - | - | - | - | - | - | - | - | 30.13 |
| 767 | 23.12.06 | Female | 5 | - | - | - | 27.05 | - | - | - | - | - | - | 30.24 | - | - | - | - | - | - | - | - | 27.65 |
| 768 | 23.12.06 | Female | 32 | - | - | - | 32.76 | - | 28.74 | - | - | - | - | - | - | - | - | - | - | - | - | - | 33.08 |
| 769 | 23.12.06 | Female | 6 | - | - | - | 31.44 | - | - | - | - | - | - | - | - | - | - | - | 30.53 | - | - | - | 31.08 |
| 770 | 23.12.06 | Male | 23 | - | - | - | 31.75 | - | - | - | - | - | - | - | - | - | - | - | - | - | - | - | 33.19 |
| 771 | 23.12.06 | Male | 20 | - | - | - | 28.97 | - | - | - | - | - | - | - | - | - | - | - | - | - | - | - | 31 |
| 772 | 23.12.06 | Male | 52 | - | - | 27.66 | 28.28 | - | - | - | - | - | - | - | - | - | - | - | - | - | 32.6 | - | 29.77 |
| 773 | 23.12.06 | Female | 45 | - | - | - | 29.18 | - | - | - | - | - | - | - | - | - | - | - | - | - | - | - | 29.52 |
| 774 | 23.12.06 | Female | 8 | - | - | - | 27.71 | - | - | - | - | - | - | - | - | - | - | - | - | - | - | - | 28.68 |
| 775 | 23.12.06 | Male | 11 | - | - | - | 29.03 | - | - | - | - | - | - | - | - | - | - | - | - | - | - | - | 31.76 |
| 776 | 23.12.06 | Female | 35 | - | - | - | 27.78 | - | - | - | - | - | - | - | - | - | - | - | - | - | - | - | 29.69 |
| 777 | 23.12.06 | Female | 16 | - | - | - | 30.1 | - | - | - | - | - | - | - | - | - | - | - | - | - | - | - | 30.34 |
| 778 | 23.12.06 | Female | 41 | 29.15 | - | - | 29.75 | - | - | - | - | - | - | - | - | - | - | - | - | - | - | 32.31 | 31.63 |
| 779 | 23.12.06 | Female | 24 | - | - | 31.45 | 29.49 | - | - | - | - | - | - | - | - | - | - | - | - | - | 38.61 | - | 30.61 |
| 780 | 23.12.06 | Female | 37 | 30.2 | - | - | 30.81 | - | - | - | - | - | - | - | - | - | - | - | - | - | - | 32 | 30.83 |
| 781 | 23.12.06 | Female | 21 | - | - | - | 29.47 | - | - | - | - | - | - | - | - | - | - | - | - | - | - | - | 28.87 |
| 782 | 23.12.06 | Male | 34 | - | - | - | 29 | - | - | - | - | - | - | - | - | - | - | - | - | - | - | - | 29.76 |
| 783 | 23.12.06 | Male | 31 | - | - | - | 30.55 | - | - | - | - | - | - | - | - | - | - | - | - | - | - | - | 30.81 |
| 784 | 23.12.07 | Male | 15 | - | - | 30.84 | 32.66 | - | - | - | 31.82 | - | - | 37.62 | - | - | - | - | - | - | 29.57 | - | 32.43 |
| 785 | 23.12.07 | Female | 39 | 29.91 | - | - | 31.87 | - | - | - | - | - | - | - | - | - | - | - | - | - | - | 32.55 | 31.73 |
| 786 | 23.12.07 | Male | 6 | - | - | - | 30.62 | - | - | - | - | - | - | - | - | - | - | - | - | - | - | - | 31.94 |
| 787 | 23.12.07 | Male | 4 | - | - | - | 30.75 | - | - | - | - | - | - | - | - | - | - | - | 35.4 | - | - | - | 33.18 |
| 788 | 23.12.07 | Female | 18 | - | - | - | 32.16 | - | - | - | - | - | - | - | - | - | - | - | - | - | - | - | 33.11 |
| 789 | 23.12.07 | Male | 22 | - | - | - | 34.27 | - | - | - | - | - | - | 35.67 | - | - | - | - | - | - | - | - | 34.76 |
| 790 | 23.12.07 | Female | 24 | - | - | 27.84 | 29.53 | - | - | - | - | - | - | - | - | - | - | - | - | - | 27.06 | - | 30.79 |
| 791 | 23.12.07 | Male | 54 | - | - | - | 32.73 | - | - | - | - | 31.06 | - | - | - | - | - | - | - | - | - | - | 33.2 |
| 792 | 23.12.07 | Male | 39 | - | - | - | 29.8 | - | - | - | - | - | - | - | - | - | - | - | - | - | - | - | 29.18 |
| 793 | 23.12.07 | Male | 62 | 26.94 | - | - | 28.88 | - | - | - | - | - | - | - | - | - | - | - | - | - | - | 27.72 | 28.31 |
| 794 | 23.12.07 | Female | 21 | - | - | - | 31.07 | - | - | - | - | - | - | - | - | - | - | - | - | - | - | - | 31.78 |
| 795 | 23.12.07 | Male | 22 | - | - | - | 29.98 | - | - | - | - | - | - | - | - | - | - | - | - | - | - | - | 30.68 |
| 796 | 23.12.07 | Male | 9 | - | - | - | 33.55 | - | - | - | - | - | - | - | - | - | - | - | - | - | - | - | 34.33 |
| 797 | 23.12.07 | Male | 38 | - | - | - | 33.63 | - | - | 32.16 | - | 32.96 | - | - | - | - | - | - | - | - | - | - | 33.15 |
| 798 | 23.12.07 | Male | 13 | - | - | - | 30.49 | - | - | - | - | - | - | - | - | - | - | - | - | - | - | - | 30.5 |
| 799 | 23.12.07 | Male | 7 | - | - | - | 28.99 | - | - | - | - | - | - | - | - | - | - | - | - | - | - | - | 29.62 |
| 800 | 23.12.07 | Female | 14 | - | - | - | 33.44 | - | - | 31.66 | - | 30.73 | - | - | - | - | - | - | - | - | - | - | 34.36 |
| 801 | 23.12.07 | Female | 8 | - | - | 32 | 32.07 | - | - | - | - | - | - | - | - | - | - | - | - | - | 31.47 | - | 31.09 |
| 802 | 23.12.07 | Female | 15 | - | - | 30.73 | 33.52 | - | - | - | - | - | - | - | - | - | - | - | - | - | 31.33 | - | 34.39 |
| 803 | 23.12.08 | Male | 7 | - | - | - | 29.92 | - | - | 24.99 | - | 28.64 | - | 28.86 | - | - | - | - | - | - | - | - | 32.16 |
| 804 | 23.12.08 | Male | 50 | - | - | - | 28.82 | - | - | - | - | - | - | - | - | - | - | - | - | - | - | - | 29.19 |
| 805 | 23.12.08 | Male | 24 | - | - | - | 34.67 | - | - | - | - | - | - | - | - | - | - | - | - | - | - | - | 34.26 |
| 806 | 23.12.08 | Male | 10 | 28.29 | - | - | 28.59 | - | - | - | - | - | - | - | - | - | - | - | - | - | - | 28.14 | 29.31 |
| 807 | 23.12.08 | Male | 33 | - | - | - | 31.28 | - | - | - | - | - | - | - | - | - | - | - | - | - | - | - | 35.03 |
| 808 | 23.12.08 | Female | 55 | - | - | - | 29.46 | - | - | - | - | - | - | - | - | - | - | - | - | - | - | - | 30.63 |
| 809 | 23.12.08 | Male | 20 | - | - | 33.54 | 30.21 | - | - | - | - | - | - | - | - | - | - | - | - | - | 34.71 | - | 30.1 |
| 810 | 23.12.08 | Female | 35 | - | - | - | 29.29 | - | - | - | - | - | - | - | - | - | - | - | - | - | - | - | 29.75 |
| 811 | 23.12.08 | Male | 27 | - | - | - | 32.12 | - | - | - | - | - | - | - | - | - | - | - | - | - | - | - | 32.74 |
| 812 | 23.12.08 | Male | 67 | - | - | - | 30.62 | - | - | - | - | - | - | - | - | - | - | - | - | - | - | - | 31.35 |
| 813 | 23.12.08 | Male | 12 | - | - | - | 34.75 | - | - | - | - | - | - | - | - | - | - | - | - | - | - | - | 34.78 |
| 814 | 23.12.08 | Male | 25 | - | - | - | 31.72 | - | - | - | - | 28.55 | - | - | - | - | - | - | - | - | - | - | 32.18 |
| 815 | 23.12.08 | Female | 7 | - | - | - | 28.13 | - | - | - | - | 26.28 | - | 26.03 | - | - | - | - | - | - | - | - | 29.22 |
| 816 | 23.12.08 | Female | 15 | - | - | - | 29.75 | - | - | - | - | - | - | - | - | - | - | - | - | - | - | - | 30.03 |
| 817 | 23.12.08 | Female | 22 | - | - | - | 29.66 | - | - | - | - | 27.52 | - | - | - | - | - | - | - | - | - | - | 29.71 |
| 818 | 23.12.08 | Female | 36 | - | - | - | 29.75 | - | - | - | - | - | - | - | - | - | - | - | - | - | - | - | 31.43 |
| 819 | 23.12.08 | Female | 15 | 27.52 | - | - | 30.68 | - | - | - | - | - | - | - | - | - | - | - | - | - | - | 25.82 | 32.1 |
| 820 | 23.12.11 | Female | 10 | - | - | 30.7 | 31.24 | - | - | - | - | - | - | - | - | - | - | - | - | - | 38.05 | - | 32.97 |
| 821 | 23.12.11 | Female | 2 | - | - | - | 31.67 | - | - | - | 33.62 | - | - | - | - | - | - | - | - | - | - | - | 32.84 |
| 822 | 23.12.11 | Female | 47 | - | - | - | 26.9 | - | - | - | - | - | - | - | - | - | - | - | - | - | - | - | 27.18 |
| 823 | 23.12.11 | Female | 3 | - | - | - | 28.72 | - | - | - | - | - | - | - | - | - | - | - | - | - | - | - | 32.39 |
| 824 | 23.12.11 | Female | 79 | - | - | - | 30.95 | - | - | - | - | - | - | - | - | - | - | - | - | - | - | - | 33.57 |
| 825 | 23.12.11 | Male | 10 | - | - | 28.77 | 30.28 | - | - | - | - | - | - | - | - | - | - | - | - | - | 29.09 | - | 31.94 |
| 826 | 23.12.11 | Male | 73 | - | - | - | 28.73 | - | - | - | - | - | - | - | - | - | - | - | - | - | - | - | 30.29 |
| 827 | 23.12.11 | Male | 18 | - | - | 30.77 | 31.26 | - | - | - | - | - | - | - | - | - | - | - | - | - | 32.34 | - | 32.17 |
| 828 | 23.12.11 | Male | 13 | 32.22 | - | - | 32.05 | - | - | - | - | - | - | - | - | - | - | - | - | - | - | 38.93 | 32.07 |
| 829 | 23.12.11 | Female | 5 | - | - | - | 34.42 | - | - | - | - | - | - | 30.9 | - | - | - | - | - | - | - | - | 36.09 |
| 830 | 23.12.11 | Male | 35 | - | - | - | 28.71 | - | - | - | - | - | - | - | - | - | - | - | - | - | - | - | 29.54 |
| 831 | 23.12.11 | Male | 25 | - | - | - | 30.7 | - | - | - | - | - | - | - | - | - | - | - | 31.88 | - | - | - | 32.65 |
| 832 | 23.12.11 | Female | 56 | - | - | 28.93 | 28.88 | - | - | - | - | - | - | - | - | - | - | - | - | - | 33.18 | - | 29.11 |
| 833 | 23.12.11 | Female | 25 | - | - | - | 29.64 | - | - | - | - | - | - | - | - | - | - | - | - | - | - | - | 28.91 |
| 834 | 23.12.11 | Female | 8 | - | - | - | 28.82 | - | - | - | - | - | - | - | - | - | - | - | - | - | - | - | 29.38 |
| 835 | 23.12.11 | Male | 7 | - | - | - | 30.65 | - | - | - | - | - | - | - | - | - | - | - | - | - | - | - | 31.99 |
| 836 | 23.12.11 | Male | 11 | - | - | 28.72 | 31.09 | - | - | - | - | - | - | - | - | - | - | - | - | - | 30.53 | - | 32.23 |
| 837 | 23.12.11 | Male | 6 | - | - | - | 32.97 | - | - | - | - | - | - | - | - | - | - | - | - | - | - | - | 35.03 |
| 838 | 23.12.11 | Male | 31 | - | - | - | 27.06 | - | - | - | - | - | - | - | - | - | - | - | - | - | - | - | 27.54 |
| 839 | 23.12.11 | Female | 4 | - | - | - | 30.57 | - | - | - | - | - | - | 31.08 | - | - | - | - | - | - | - | - | 31.76 |
| 840 | 23.12.11 | Female | 28 | - | - | 28.05 | 27.59 | - | - | - | - | - | - | - | - | - | - | - | - | - | 36.9 | - | 27.61 |
| 841 | 23.12.11 | Female | 10 | - | - | - | 28 | - | - | - | - | - | - | - | - | - | - | - | - | - | - | - | 29.16 |
| 842 | 23.12.11 | Female | 41 | - | - | - | 30.51 | - | - | - | - | - | - | - | - | - | - | - | - | - | - | - | 30.22 |
| 843 | 23.12.11 | Female | 42 | - | - | - | 26.16 | - | - | - | - | - | - | - | - | - | - | - | - | - | - | - | 26.94 |
| 844 | 23.12.11 | Female | 49 | - | - | - | 27.2 | - | - | - | - | - | - | - | - | - | - | - | 29.71 | - | - | - | 27.43 |
| 845 | 23.12.11 | Female | 53 | - | - | - | 29.81 | - | - | - | - | - | - | - | - | - | - | - | - | - | - | - | 30.58 |
| 846 | 23.12.11 | Female | 5 | 29.2 | - | - | 29.7 | - | - | - | - | - | - | - | - | - | - | - | - | - | - | 30.25 | 33.73 |
| 847 | 23.12.11 | Female | 48 | - | - | - | 26.83 | - | - | - | - | - | - | - | - | - | - | - | - | - | - | - | 27.95 |
| 848 | 23.12.11 | Female | 24 | - | - | - | 28.77 | - | - | - | - | 28.17 | - | - | - | - | - | - | - | - | - | - | 30.18 |
| 849 | 23.12.11 | Female | 10 | - | - | - | 29.16 | - | - | - | - | - | - | 27.72 | - | - | - | - | - | - | - | - | 30.19 |
| 850 | 23.12.12 | Male | 7 | - | - | 29.88 | 30.77 | - | - | - | - | - | - | - | - | - | - | - | - | - | 36.13 | - | 31.16 |
| 851 | 23.12.12 | Female | 12 | - | - | - | 33.52 | - | - | - | - | - | - | - | - | - | - | - | - | - | - | - | 33.55 |
| 852 | 23.12.12 | Female | 2 | - | - | - | 32.62 | - | - | - | - | - | - | - | - | - | - | - | - | - | - | - | 33.79 |
| 853 | 23.12.12 | Female | 14 | - | - | - | 28.44 | - | - | - | - | - | - | - | - | - | - | - | - | - | - | - | 32.16 |
| 854 | 23.12.12 | Female | 38 | - | - | 26.57 | 27.85 | - | - | - | - | - | - | - | - | - | - | - | - | - | 27.72 | - | 29.07 |
| 855 | 23.12.12 | Male | 23 | 27.9 | - | - | 30.91 | - | - | - | - | - | - | - | - | - | - | - | - | - | - | 28.19 | 31.4 |
| 856 | 23.12.12 | Female | 7 | 35.49 | - | - | 32.04 | - | - | - | - | - | - | - | - | - | - | - | - | - | - | 35.79 | 33.07 |
| 857 | 23.12.12 | Male | 9 | - | - | - | 29.75 | - | 27.18 | - | - | - | - | - | - | - | - | - | - | - | - | - | 31.07 |
| 858 | 23.12.12 | Female | 21 | - | - | - | 30.03 | - | - | - | - | 28.72 | - | - | - | - | - | - | - | - | - | - | 30.11 |
| 859 | 23.12.13 | Female | 9 | - | - | - | 34.55 | - | - | - | - | - | - | - | - | - | - | - | - | - | - | - | 34.59 |
| 860 | 23.12.13 | Female | 39 | - | - | 31.57 | 30.21 | - | - | - | - | - | - | - | - | - | - | - | - | - | 36.97 | - | 30.75 |
| 861 | 23.12.13 | Female | 34 | 30.64 | - | - | 30.12 | - | - | - | - | - | - | - | - | - | - | - | - | - | - | 34.33 | 34.04 |
| 862 | 23.12.13 | Male | 66 | - | - | - | 25.66 | - | - | - | - | - | - | - | - | - | - | - | - | - | - | - | 27.71 |
| 863 | 23.12.13 | Female | 42 | - | - | - | 25.85 | - | - | - | - | - | - | 26.63 | - | - | - | - | - | - | - | - | 24.3 |
| 864 | 23.12.13 | Male | 44 | 32.13 | - | - | 29.78 | - | - | - | - | - | - | - | - | - | - | - | - | - | - | 34.22 | 29.25 |
| 865 | 23.12.13 | Female | 37 | - | - | - | 24.86 | - | - | - | - | - | - | - | - | - | - | - | - | - | - | - | 26.12 |
| 866 | 23.12.13 | Male | 20 | - | - | - | 28.66 | - | - | - | - | - | - | - | - | - | - | - | - | - | - | - | 29.21 |
| 867 | 23.12.13 | Male | 29 | - | - | 30.84 | 31.9 | - | - | - | - | - | - | - | - | - | - | - | - | - | - | - | 32.68 |
| 868 | 23.12.13 | Male | 18 | - | - | 26.79 | 28.67 | - | - | - | - | - | - | - | - | - | - | - | - | - | 29.71 | - | 30.79 |
| 869 | 23.12.13 | Male | 14 | 29.44 | - | - | 30.94 | - | - | - | - | - | - | - | - | - | - | - | - | - | - | 31.36 | 33.67 |
| 870 | 23.12.13 | Male | 14 | 26.31 | - | - | 27.7 | - | - | - | - | - | - | - | - | - | - | - | - | - | - | 29.11 | 31.12 |
| 871 | 23.12.13 | Male | 12 | - | - | - | 31.65 | - | - | - | - | - | - | - | - | - | - | - | - | - | - | - | 35.31 |
| 872 | 23.12.13 | Female | 9 | - | - | - | 27.84 | - | - | - | - | - | - | - | - | - | - | - | - | - | - | - | 29.73 |
| 873 | 23.12.13 | Female | 4 | - | - | - | 26.49 | - | - | - | - | - | - | - | - | - | - | - | - | - | - | - | 29.44 |
| 874 | 23.12.13 | Male | 34 | 28.85 | - | - | 29.1 | - | - | - | - | - | - | - | - | - | - | - | - | - | - | 33.95 | 30 |
| 875 | 23.12.13 | Female | 21 | - | - | - | 30.45 | - | - | - | - | - | - | - | - | - | - | - | - | - | - | - | 32.19 |
| 876 | 23.12.14 | Male | 6 | - | - | - | 31.1 | - | - | - | - | - | - | - | - | - | - | - | - | - | - | - | 34.17 |
| 877 | 23.12.14 | Male | 13 | - | - | - | 30.89 | - | - | - | - | - | - | - | - | - | - | - | - | - | - | - | 33.4 |
| 878 | 23.12.14 | Male | 13 | 32.07 | - | - | 31.72 | - | - | - | - | - | - | - | - | - | - | - | - | - | - | 39.04 | 32.32 |
| 879 | 23.12.14 | Male | 14 | - | - | - | 31.86 | - | - | - | - | - | - | - | - | - | - | - | - | - | - | - | 32.7 |
| 880 | 23.12.14 | Male | 5 | - | - | - | 30.86 | - | - | - | - | - | - | - | - | - | - | - | - | - | - | - | 34.63 |
| 881 | 23.12.14 | Female | 2 | - | - | - | 29.99 | - | - | - | - | - | - | - | - | - | - | - | - | - | - | - | 34.4 |
| 882 | 23.12.14 | Female | 41 | - | - | - | 31.49 | - | - | - | - | - | - | - | - | - | - | - | - | - | - | - | 31.53 |
| 883 | 23.12.14 | Male | 15 | - | - | - | 33 | - | - | - | - | - | - | - | - | - | - | - | - | - | - | - | 33.07 |
| 884 | 23.12.14 | Female | 52 | 32.75 | - | - | 26.66 | - | - | - | - | - | - | - | - | - | - | - | - | - | - | 34.5 | 27.2 |
| 885 | 23.12.14 | Female | 42 | - | - | - | 26.11 | - | - | - | - | - | - | - | - | - | - | - | - | - | - | - | 28.87 |
| 886 | 23.12.14 | Male | 6 | - | - | 26.57 | 28.34 | - | - | - | - | - | - | - | - | - | - | - | - | - | 28.48 | - | 31.73 |
| 887 | 23.12.14 | Female | 27 | - | - | - | 32.65 | - | - | - | - | - | - | - | - | - | - | - | - | - | - | - | 33.47 |
| 888 | 23.12.14 | Male | 27 | - | - | - | 31.55 | - | - | - | - | - | - | - | - | - | - | - | - | - | - | - | 32.36 |
| 889 | 23.12.14 | Male | 26 | - | - | 30.99 | 29.55 | - | - | - | - | - | - | - | - | - | - | - | - | - | 34.04 | - | 30.22 |
| 890 | 23.12.14 | Female | 7 | - | - | 32.55 | 31.83 | - | - | - | - | - | - | - | - | - | - | - | - | - | 33.81 | - | 31.94 |
| 891 | 23.12.14 | Male | 14 | - | - | - | 27.87 | - | - | - | - | 25.69 | - | - | - | - | - | - | - | - | - | - | 29.14 |
| 892 | 23.12.14 | Male | 5 | - | - | - | 29.08 | - | - | - | - | - | - | - | - | - | - | - | - | - | - | - | 32.71 |
| 893 | 23.12.14 | Male | 21 | - | 29.58 | 29.1 | 28.94 | - | - | - | - | - | - | - | - | - | - | - | - | - | 32.07 | - | 31.05 |
| 894 | 23.12.14 | Male | 33 | - | - | - | 28.54 | - | - | - | - | - | - | - | - | - | - | - | - | - | - | - | 32.33 |
| 895 | 23.12.14 | Female | 11 | - | - | - | 30.48 | - | - | - | - | - | - | - | - | - | - | - | - | - | - | - | 31.03 |
| 896 | 23.12.14 | Male | 36 | - | 33.83 | - | 32.68 | - | - | - | - | - | - | - | - | - | - | - | - | - | - | - | 34.44 |
| 897 | 23.12.15 | Female | 43 | - | - | - | 29.61 | - | - | - | - | - | - | - | - | - | - | - | - | - | - | - | 30.42 |
| 898 | 23.12.15 | Female | 24 | - | - | - | 28.68 | - | - | - | - | - | - | - | - | - | - | - | - | - | - | - | 28.72 |
| 899 | 23.12.15 | Female | 9 | - | - | - | 29.47 | - | - | - | - | - | - | - | - | - | - | - | - | - | - | - | 33.55 |
| 900 | 23.12.15 | Female | 60 | - | - | - | 25.88 | - | - | - | - | - | - | - | - | - | - | - | - | - | - | - | 29.96 |
| 901 | 23.12.15 | Male | 55 | - | - | - | 32.05 | - | - | 28.45 | - | 28.57 | - | - | - | - | - | - | - | - | - | - | 32.73 |
| 902 | 23.12.15 | Female | 34 | - | - | 31 | 32.83 | - | - | - | - | - | - | - | - | - | - | - | - | - | 31.06 | - | 33.66 |
| 903 | 23.12.15 | Female | 46 | - | - | - | 29.5 | - | - | - | - | - | - | - | - | - | - | - | - | - | - | - | 29.35 |
| 904 | 23.12.15 | Female | 31 | - | - | - | 32.99 | - | - | - | - | - | - | - | - | - | - | - | - | - | - | - | 33.63 |
| 905 | 23.12.15 | Female | 60 | - | - | - | 30.2 | - | - | 26.5 | - | 28.46 | - | - | - | - | - | - | - | - | - | - | 30.45 |
| 906 | 23.12.15 | Male | 6 | - | - | - | 31.04 | - | - | - | - | - | - | 31.31 | - | - | - | - | - | - | - | - | 30.31 |
| 907 | 23.12.15 | Male | 17 | - | - | - | 34 | - | - | - | - | - | - | - | - | - | - | - | - | - | - | - | 34.63 |
| 908 | 23.12.15 | Male | 52 | - | - | - | 28.47 | - | - | - | - | - | - | - | - | - | - | - | - | - | - | - | 29.13 |
| 909 | 23.12.15 | Male | 50 | - | - | - | 27.65 | - | - | - | - | 27.85 | - | - | - | - | - | - | - | - | - | - | 27.99 |
| 910 | 23.12.15 | Female | 33 | - | - | - | 33.11 | - | - | - | - | - | - | - | - | - | - | - | - | - | - | - | 33.05 |
| 911 | 23.12.15 | Female | 29 | - | - | - | 29.69 | - | - | - | - | - | - | 27.1 | - | - | - | - | - | - | - | - | 29.21 |
| 912 | 23.12.15 | Male | 51 | - | - | - | 30.84 | - | - | - | - | - | - | - | - | - | - | - | 32.17 | - | - | - | 31.1 |
| 913 | 23.12.15 | Male | 9 | 31.51 | - | - | 29.66 | - | - | - | - | - | - | - | - | - | - | - | - | - | - | 34.34 | 30.69 |
| 914 | 23.12.15 | Female | 50 | 29.51 | - | - | 27.61 | - | - | - | - | - | - | - | - | - | - | - | - | - | - | 31.9 | 27.26 |
| 915 | 23.12.15 | Male | 37 | - | - | - | 33.93 | - | - | - | - | - | - | 30.2 | - | - | - | - | - | - | - | - | 33.29 |
| 916 | 23.12.15 | Female | 20 | 31.2 | - | - | 32.12 | - | - | - | - | - | - | - | - | - | - | - | - | - | - | 33.6 | 33.76 |
| 917 | 23.12.15 | Male | 4 | - | 30.93 | - | 31.48 | - | - | - | - | - | - | - | - | - | - | - | - | - | - | - | 34.28 |
| 918 | 23.12.15 | Female | 32 | 31.54 | - | - | 31.71 | - | - | - | - | - | - | - | - | - | - | - | - | - | - | 37.73 | 33.72 |
| 919 | 23.12.15 | Female | 30 | - | - | - | 31.9 | - | - | - | - | - | - | - | - | - | - | - | - | - | - | - | 34.45 |
| 920 | 23.12.15 | Male | 52 | - | - | - | 26.9 | - | - | - | - | - | - | - | - | - | - | - | - | - | - | - | 27.46 |
| 921 | 23.12.15 | Male | 36 | - | - | - | 27.97 | - | - | - | - | - | - | - | - | - | - | - | - | - | - | - | 30.91 |
| 922 | 23.12.15 | Female | 7 | - | - | - | 28.76 | - | - | - | - | - | - | - | - | - | - | - | - | - | - | - | 29.05 |
| 923 | 23.12.18 | Female | 6 | - | - | - | 27.98 | - | - | - | - | - | - | 28.98 | - | - | - | - | - | - | - | - | 28.76 |
| 924 | 23.12.18 | Male | 8 | - | - | 32.45 | 32.72 | - | - | - | - | - | - | - | - | - | - | - | - | - | 35.33 | - | 33.95 |
| 925 | 23.12.18 | Female | 41 | - | - | - | 31.55 | - | - | - | - | 28.79 | - | - | - | - | - | - | - | - | - | - | 33.6 |
| 926 | 23.12.18 | Male | 3 | - | - | - | 31.3 | - | - | - | - | - | - | - | - | - | - | - | - | - | - | - | 32.52 |
| 927 | 23.12.18 | Male | 6 | - | - | - | 29.88 | - | - | - | 26.88 | - | - | - | - | - | - | 27.97 | - | - | - | - | 31.12 |
| 928 | 23.12.18 | Male | 44 | - | - | - | 30.64 | - | - | - | - | 30.7 | - | 30.54 | - | - | - | - | - | - | - | - | 30.76 |
| 929 | 23.12.19 | Female | 67 | - | - | - | 28.76 | - | - | - | - | - | - | 31.99 | - | - | - | - | - | - | - | - | 29.69 |
| 930 | 23.12.19 | Male | 5 | - | - | 29.05 | 31.07 | - | - | - | - | - | - | - | - | - | - | - | - | - | 29.31 | - | 32.26 |
| 931 | 23.12.19 | Female | 34 | - | - | 32.08 | 31.55 | - | - | - | - | - | - | - | - | - | - | - | - | - | 34.45 | - | 32.09 |
| 932 | 23.12.19 | Female | 23 | - | - | - | 30.99 | - | - | - | - | - | - | - | - | - | - | - | - | - | - | - | 33.29 |
| 933 | 23.12.19 | Male | 11 | - | - | 27.64 | 25.46 | - | - | - | - | - | - | - | - | - | - | - | - | - | 30.75 | - | 25.78 |
| 934 | 23.12.19 | Male | 13 | 30.51 | - | - | 31.92 | - | - | - | - | - | - | - | - | - | - | - | - | - | - | 31.8 | 35.01 |
| 935 | 23.12.19 | Male | 21 | - | - | 31.94 | 31.49 | - | - | - | - | - | - | - | - | - | - | - | - | - | 35.35 | - | 32.37 |
| 936 | 23.12.19 | Male | 15 | - | - | 25.88 | 28.57 | - | - | - | - | - | - | - | - | - | - | - | - | - | 27.08 | - | 30.01 |
| 937 | 23.12.19 | Male | 18 | - | - | 24.11 | 27.97 | - | - | - | - | - | - | - | - | - | - | - | - | - | 23.34 | - | 29.55 |
| 938 | 23.12.19 | Male | 6 | - | - | - | 30.58 | - | - | - | - | - | - | 27.85 | - | - | - | - | - | - | - | - | 33.28 |
| 939 | 23.12.19 | Male | 5 | 27.98 | - | - | 29.35 | - | - | - | - | - | - | - | - | - | - | - | - | - | - | 32.14 | 30.26 |
| 940 | 23.12.19 | Female | 41 | - | - | 26.4 | 27.15 | - | - | - | - | - | - | - | - | - | - | - | - | - | 34.37 | - | 26.93 |
| 941 | 23.12.19 | Female | 41 | - | - | - | 31.77 | - | - | - | - | - | - | 31.72 | - | - | - | - | - | - | - | - | 34.88 |
| 942 | 23.12.19 | Female | 55 | - | - | - | 30.74 | - | - | - | - | - | - | - | - | - | - | - | - | - | - | - | 30.88 |
| 943 | 23.12.19 | Female | 4 | - | - | - | 30.89 | - | - | - | - | - | - | - | - | - | - | - | - | - | - | - | 30.73 |
| 944 | 23.12.19 | Female | 13 | - | - | - | 30.29 | - | - | - | - | - | - | - | - | - | - | - | - | - | - | - | 30.91 |
| 945 | 23.12.20 | Female | 60 | - | - | - | 27.95 | - | - | - | - | - | - | - | - | - | - | - | - | - | - | - | 29.06 |
| 946 | 23.12.20 | Male | 36 | - | - | - | 31 | - | - | - | - | - | - | - | - | - | - | - | - | - | - | - | 31.05 |
| 947 | 23.12.20 | Female | 46 | - | - | - | 29.94 | - | - | - | - | - | - | - | - | - | - | - | - | - | - | - | 28.89 |
| 948 | 23.12.20 | Male | 61 | - | - | - | 32.95 | - | - | - | - | - | - | - | - | - | - | - | - | - | - | - | 33.5 |
| 949 | 23.12.20 | Male | 16 | - | - | - | 29.87 | - | - | - | - | - | - | 31.39 | - | - | - | - | - | - | - | - | 31.23 |
| 950 | 23.12.20 | Male | 40 | - | - | 29.16 | 30.53 | - | - | - | - | - | - | - | - | - | - | - | - | - | 30.62 | - | 33.66 |
| 951 | 23.12.20 | Male | 36 | - | - | - | 31.21 | - | - | - | - | - | - | 37.5 | - | - | - | - | - | - | - | - | 32.04 |
| 952 | 23.12.20 | Female | 32 | - | 31.68 | - | 29.63 | - | - | - | - | - | - | - | - | - | - | - | - | - | - | - | 29.27 |
| 953 | 23.12.20 | Male | 8 | - | 30.16 | - | 31.13 | - | - | - | - | - | - | - | - | - | - | - | - | - | - | - | 30.72 |
| 954 | 23.12.20 | Female | 6 | - | - | - | 26.14 | - | - | - | - | - | - | - | - | - | - | - | - | - | - | - | 26.68 |
| 955 | 23.12.20 | Female | 47 | - | - | - | 26.5 | - | - | - | - | - | - | - | - | - | - | - | - | - | - | - | 26.94 |
| 956 | 23.12.20 | Female | 37 | - | - | - | 28.07 | - | - | - | - | - | - | 28.15 | - | - | - | - | - | - | - | - | 27.85 |
| 957 | 23.12.20 | Male | 6 | - | 32.12 | - | 31.13 | - | - | - | - | - | - | 37 | - | - | - | - | - | - | - | - | 31.28 |
| 958 | 23.12.20 | Male | 13 | - | - | 27.61 | 31.19 | - | - | - | - | - | - | - | - | - | - | - | - | - | 28.11 | - | 32.37 |
| 959 | 23.12.20 | Female | 1 | - | - | - | 26.62 | - | - | - | - | - | - | - | - | - | - | - | - | - | - | - | 28.8 |
| 960 | 23.12.20 | Female | 55 | - | - | - | 27.52 | - | - | - | - | - | - | - | - | - | - | - | - | - | - | - | 27.72 |
| 961 | 23.12.20 | Male | 6 | - | - | 28.14 | 32.4 | - | - | - | - | - | - | - | - | - | - | - | - | - | 26.3 | - | 32.42 |
| 962 | 23.12.20 | Male | 5 | - | - | - | 29.29 | - | - | - | - | - | - | - | - | - | - | - | - | - | - | - | 29.95 |
| 963 | 23.12.20 | Male | 6 | - | - | 27.84 | 27.59 | - | - | - | - | - | - | - | - | - | - | - | - | - | 27.09 | - | 27.97 |
| 964 | 23.12.20 | Male | 6 | - | - | 30.68 | 31.08 | - | - | - | - | - | - | - | - | - | - | - | - | - | 32.54 | - | 32.18 |
| 965 | 23.12.20 | Female | 14 | - | - | 31.79 | 30.81 | - | - | - | - | - | - | - | - | - | - | - | - | - | 35.35 | - | 32.53 |
| 966 | 23.12.21 | Female | 4 | - | - | - | 29.84 | - | - | - | - | - | - | - | - | - | - | - | - | - | - | - | 31.15 |
| 967 | 23.12.21 | Female | 40 | - | - | - | 31.94 | - | - | - | - | - | - | - | - | - | - | - | - | - | - | - | 34.94 |
| 968 | 23.12.21 | Female | 17 | - | - | 31.15 | 31.18 | - | - | - | - | - | - | - | - | - | - | - | - | - | 34.19 | - | 34.67 |
| 969 | 23.12.22 | Male | 31 | - | - | - | 31.77 | - | - | - | - | - | - | - | - | - | - | - | - | - | - | - | 33.42 |
| 970 | 23.12.22 | Female | 5 | - | - | - | 28.56 | - | - | - | - | - | - | - | - | - | - | - | - | - | - | - | 30.78 |
| 971 | 23.12.22 | Male | 33 | - | - | - | 29.71 | - | - | - | - | - | - | - | - | - | - | - | - | - | - | - | 31.24 |
| 972 | 23.12.22 | Female | 7 | - | - | - | 28.94 | - | - | - | - | - | - | 25.45 | - | - | - | - | - | - | - | - | 29.01 |
| 973 | 23.12.22 | Female | 39 | - | - | - | 31.72 | - | - | - | - | - | - | - | - | - | - | - | - | - | - | - | 32.96 |
| 974 | 23.12.22 | Female | 5 | - | - | - | 29.11 | - | - | - | - | - | - | - | - | - | - | - | - | - | - | - | 30.47 |
| 975 | 23.12.22 | Female | 25 | 31.97 | - | - | 31.02 | - | 27.53 | - | - | - | - | - | - | - | - | - | - | - | - | - | 32.88 |
| 976 | 23.12.25 | Male | 48 | 30.52 | - | - | 28.86 | - | - | - | - | - | - | - | - | - | - | - | - | - | - | - | 29.6 |
| 977 | 23.12.25 | Female | 42 | - | - | - | 28.52 | - | - | - | - | - | - | - | - | - | - | - | - | - | - | - | 27.81 |
| 978 | 23.12.25 | Female | 7 | - | - | - | 30.6 | - | - | - | - | - | - | - | - | - | - | - | - | - | - | - | 31.83 |
| 979 | 23.12.25 | Female | 49 | - | - | 29.46 | 30.22 | - | - | - | - | - | - | - | - | - | - | - | - | - | 29.74 | - | 31.19 |
| 980 | 23.12.25 | Female | 10 | - | - | - | 32.32 | - | - | - | - | - | - | - | - | - | - | - | - | - | - | - | 36.21 |
| 981 | 23.12.25 | Male | 8 | - | 30.38 | - | 31.97 | - | - | - | - | - | - | - | - | - | - | - | - | - | - | - | 32.86 |
| 982 | 23.12.25 | Female | 7 | - | - | 31.84 | 31.78 | - | - | - | - | - | - | - | - | - | - | - | - | - | 35.29 | - | 32.69 |
| 983 | 23.12.25 | Male | 16 | - | - | - | 34.18 | - | - | - | - | - | - | - | - | - | - | - | - | - | - | - | 38.41 |
| 984 | 23.12.25 | Female | 42 | - | - | - | 29.07 | - | - | - | - | - | - | - | - | - | - | - | - | - | - | - | 29.2 |
| 985 | 23.12.25 | Female | 41 | 28.78 | - | - | 30.28 | - | - | - | - | - | - | - | - | - | - | - | - | - | - | 32.05 | 30.57 |
| 986 | 23.12.25 | Male | 21 | 23.85 | - | - | 28.01 | - | - | - | - | - | - | - | - | - | - | - | - | - | - | 24.96 | 29.43 |
| 987 | 23.12.25 | Female | 27 | - | - | 30.57 | 30.78 | - | - | - | - | - | - | - | - | - | - | - | - | - | 37.84 | - | 32.65 |
| 988 | 23.12.25 | Female | 26 | - | - | - | 30.8 | - | - | - | - | - | - | - | - | - | - | - | - | - | - | - | 36.1 |
| 989 | 23.12.25 | Male | 5 | - | - | - | 31.61 | - | - | - | - | - | - | 33.03 | - | - | - | - | - | - | - | - | 33.81 |
| 990 | 23.12.25 | Male | 18 | - | - | - | 31.99 | - | - | - | - | - | - | - | - | - | - | - | - | - | - | - | 34.35 |
| 991 | 23.12.25 | Male | 20 | - | - | - | 31.57 | - | - | - | - | - | - | - | - | - | - | - | - | - | - | - | 32.37 |
| 992 | 23.12.25 | Female | 56 | - | - | - | 28.81 | - | - | - | - | - | - | - | - | - | - | - | - | - | - | - | 28.72 |
| 993 | 23.12.25 | Male | 21 | - | - | - | 29.26 | - | - | - | - | - | - | - | - | - | - | - | - | - | - | - | 29.22 |
| 994 | 23.12.25 | Male | 31 | - | - | - | 28.03 | - | - | - | - | - | - | - | - | - | - | - | - | - | - | - | 30.9 |
| 995 | 23.12.25 | Female | 8 | - | - | - | 30.93 | - | - | - | - | - | - | 28.48 | - | - | - | - | - | - | - | - | 33.98 |
| 996 | 23.12.25 | Male | 27 | - | - | - | 30.87 | - | - | - | - | - | - | - | - | - | - | - | - | - | - | - | 32.31 |
| 997 | 23.12.25 | Female | 14 | - | - | - | 33.2 | - | - | - | - | 27.99 | - | - | - | - | - | - | - | - | - | - | 33.39 |
| 998 | 23.12.25 | Male | 36 | - | - | - | 30.29 | - | - | - | - | - | - | - | - | - | - | - | - | - | - | - | 29.7 |
| 999 | 23.12.25 | Male | 5 | - | - | - | 31.61 | - | - | - | - | - | - | - | - | - | - | - | - | - | - | - | 32.62 |
| 1000 | 23.12.25 | Female | 27 | - | - | 30.76 | 32.5 | - | - | - | - | - | - | - | - | - | - | - | - | - | 30.44 | - | 34.36 |
| 1001 | 23.12.25 | Male | 27 | - | - | 28.62 | 27.6 | - | - | - | - | - | - | - | - | - | - | - | - | - | 37.94 | - | 30.92 |
| 1002 | 23.12.26 | Male | 20 | - | - | 28.52 | 30.43 | - | - | - | - | - | - | - | - | - | - | - | - | - | 28.32 | - | 32.44 |
| 1003 | 23.12.26 | Female | 40 | - | - | - | 31.75 | - | - | - | - | - | - | - | - | - | - | - | - | - | - | - | 33.04 |
| 1004 | 23.12.26 | Male | 6 | - | - | - | 32.59 | - | - | - | - | - | - | 40 | - | - | - | - | - | - | - | - | 36.23 |
| 1005 | 23.12.26 | Female | 49 | - | - | 32.9 | 32.16 | - | - | - | - | - | - | - | - | - | - | - | - | - | 36.09 | - | 32.22 |
| 1006 | 23.12.26 | Female | 5 | - | - | - | 31.89 | - | - | - | - | 28.98 | - | - | - | 33.19 | - | - | - | - | - | - | 33.69 |
| 1007 | 23.12.26 | Female | 2 | - | - | 27.45 | 29.58 | - | - | - | - | - | - | - | - | - | - | - | - | - | 27.56 | - | 31.48 |
| 1008 | 23.12.26 | Male | 35 | - | - | - | 29.21 | - | - | - | - | - | - | - | - | - | - | - | - | - | - | - | 29.95 |
| 1009 | 23.12.26 | Female | 51 | - | - | - | 27.01 | - | - | - | - | - | - | - | - | - | - | - | - | - | - | - | 27.55 |
| 1010 | 23.12.26 | Female | 45 | - | - | - | 27.55 | - | - | - | - | - | - | - | - | - | - | - | - | - | - | - | 28.39 |
| 1011 | 23.12.26 | Female | 44 | - | - | - | 30.27 | - | - | - | - | - | - | - | - | - | - | - | - | - | - | - | 32.9 |
| 1012 | 23.12.26 | Female | 33 | - | - | - | 31.52 | - | - | - | - | - | - | 31.38 | - | - | - | - | - | - | - | - | 33.15 |
| 1013 | 23.12.26 | Male | 19 | 33.33 | - | - | 30.72 | - | - | - | - | - | - | - | - | - | - | - | - | - | - | 34.87 | 33.13 |
| 1014 | 23.12.26 | Male | 26 | - | - | - | 31.17 | - | - | - | - | - | - | - | - | - | - | - | - | - | - | - | 33.22 |
| 1015 | 23.12.26 | Female | 26 | - | - | - | 30.74 | - | - | - | - | - | - | - | - | - | - | - | - | - | - | - | 34 |
| 1016 | 23.12.26 | Male | 19 | - | - | - | 28.55 | - | - | - | - | - | - | - | - | - | - | - | - | - | - | - | 29 |
| 1017 | 23.12.26 | Female | 42 | - | - | - | 32.53 | - | - | - | - | 30.12 | - | - | - | - | - | - | - | - | - | - | 33.02 |
| 1018 | 23.12.26 | Male | 46 | - | - | - | 26.92 | - | - | - | - | - | - | - | - | - | - | - | - | - | - | - | 27.5 |
| 1019 | 23.12.26 | Female | 26 | - | - | - | 28.25 | - | - | - | - | - | - | - | - | - | - | - | - | - | - | - | 30.13 |
| 1020 | 23.12.26 | Male | 27 | - | - | - | 30.25 | - | - | - | - | - | - | - | - | - | - | - | - | - | - | - | 33.68 |
| 1021 | 23.12.26 | Male | 40 | - | - | 30.96 | 28.42 | - | - | - | - | - | - | - | - | - | - | - | - | - | 36.16 | - | 29.73 |
| 1022 | 23.12.26 | Male | 70 | - | - | - | 26.74 | - | - | - | - | - | - | - | - | - | - | - | - | - | - | - | 30.06 |
| 1023 | 23.12.26 | Female | 35 | - | - | - | 29.68 | - | - | - | - | - | - | - | - | - | - | - | - | - | - | - | 32.8 |
| 1024 | 23.12.26 | Male | 56 | - | - | - | 26.87 | - | - | - | - | - | - | - | - | - | - | - | - | - | - | - | 28.17 |
| 1025 | 23.12.26 | Female | 44 | - | - | 28.91 | 25.7 | - | - | - | - | - | - | - | - | - | - | - | - | - | 32.29 | - | 25.4 |
| 1026 | 23.12.26 | Female | 46 | - | - | - | 27.49 | - | - | - | - | - | - | - | - | - | - | - | - | - | - | - | 28.28 |
| 1027 | 23.12.26 | Female | 17 | - | - | - | 30.04 | - | - | - | - | - | - | - | - | - | - | - | - | - | - | - | 30.07 |
| 1028 | 23.12.27 | Female | 53 | - | - | - | 29.71 | - | - | - | - | - | - | - | - | - | - | - | - | - | - | - | 29.55 |
| 1029 | 23.12.27 | Female | 23 | - | - | 34.16 | 34.15 | - | - | - | - | - | - | - | - | - | - | - | - | - | 35.36 | - | 36.1 |
| 1030 | 23.12.27 | Male | 28 | - | - | - | 28.48 | - | 25.91 | - | - | - | - | - | - | - | - | - | - | - | - | - | 28.53 |
| 1031 | 23.12.27 | Female | 45 | - | - | 29.47 | 29.3 | - | - | - | - | - | - | - | - | - | - | - | - | - | 31.2 | - | 29.68 |
| 1032 | 23.12.27 | Male | 17 | - | - | - | 30.55 | - | - | 26.15 | - | 27.99 | - | - | - | - | - | - | - | - | - | - | 31.82 |
| 1033 | 23.12.27 | Male | 29 | - | - | - | 28.98 | - | - | - | - | - | - | - | - | - | - | - | - | - | - | - | 28.9 |
| 1034 | 23.12.27 | Female | 21 | - | - | - | 31.64 | - | - | - | - | - | - | - | - | - | - | - | - | - | - | - | 34.86 |
| 1035 | 23.12.27 | Male | 48 | 30.55 | - | - | 29.83 | - | - | - | - | - | - | - | - | - | - | - | - | - | - | 34.32 | 31.08 |
| 1036 | 23.12.27 | Female | 44 | - | - | 30.15 | 31.52 | - | - | - | - | - | - | - | - | - | - | - | - | - | 34.36 | - | 33.24 |
| 1037 | 23.12.28 | Male | 51 | 33.81 | - | - | 30.55 | - | - | - | - | - | - | - | - | - | - | - | - | - | - | 34.5 | 30.22 |
| 1038 | 23.12.28 | Male | 11 | 28.94 | - | 24.79 | 30.34 | - | - | - | - | - | - | - | - | - | - | - | - | - | 24.3 | 30.25 | 32.01 |
| 1039 | 23.12.28 | Female | 52 | - | - | - | 30.71 | - | - | - | - | - | - | - | - | - | - | - | - | - | - | - | 31.75 |
| 1040 | 23.12.28 | Male | 12 | 28.85 | - | - | 28.6 | - | - | - | - | - | - | - | - | - | - | - | - | - | - | 31.55 | 28.99 |
| 1041 | 23.12.28 | Male | 12 | - | - | - | 25.72 | - | - | - | - | - | - | - | - | - | - | - | - | - | - | - | 24.97 |
| 1042 | 23.12.28 | Female | 42 | - | - | - | 30.2 | - | - | - | - | 27.58 | - | - | - | - | - | - | - | - | - | - | 32.45 |
| 1043 | 23.12.28 | Female | 35 | 32.67 | - | - | 30.73 | - | - | - | - | - | - | - | - | - | - | - | - | - | - | 38.15 | 31.61 |
| 1044 | 23.12.28 | Female | 40 | - | - | - | 32.07 | - | - | - | - | - | - | 36.27 | - | - | - | - | - | - | - | - | 32.4 |
| 1045 | 23.12.28 | Female | 35 | - | - | - | 31.76 | - | - | - | - | - | - | - | - | - | - | - | - | - | - | - | 32.89 |
| 1046 | 23.12.28 | Female | 28 | - | - | - | 25.89 | - | - | - | - | - | - | - | - | - | - | - | - | - | - | - | 26.7 |
| 1047 | 23.12.28 | Female | 33 | 30.06 | - | - | 29.73 | - | - | - | - | - | - | - | - | - | - | - | 35.68 | - | - | 36.68 | 32.59 |
| 1048 | 23.12.28 | Female | 5 | - | - | - | 29.95 | - | - | - | - | - | - | - | - | - | - | - | - | - | - | - | 34.39 |
| 1049 | 23.12.28 | Male | 6 | - | - | - | 27.45 | - | - | - | - | 28.05 | - | - | - | - | - | - | - | - | - | - | 33.09 |
| 1050 | 23.12.28 | Male | 53 | - | - | - | 28.97 | - | - | - | - | - | - | - | - | - | - | - | - | - | - | - | 30.16 |
| 1051 | 23.12.28 | Female | 33 | - | - | 32.15 | 31.02 | - | - | - | - | - | - | - | - | - | - | - | - | - | 33.7 | - | 31.35 |
| 1052 | 23.12.28 | Male | 6 | - | - | - | 32.2 | - | - | - | - | - | - | - | - | - | - | - | - | - | - | - | 32.44 |
| 1053 | 23.12.28 | Female | 39 | - | - | 32.17 | 29.83 | - | - | - | - | - | - | - | - | - | - | - | - | - | 33.49 | - | 29.83 |
| 1054 | 23.12.28 | Female | 18 | - | - | 28.01 | 30.17 | - | - | - | - | - | - | - | - | - | - | - | - | - | 30.04 | - | 32.27 |
| 1055 | 23.12.28 | Male | 16 | - | - | 29.16 | 29.23 | - | - | - | - | - | - | - | - | - | - | - | - | - | 38.02 | - | 30.81 |
| 1056 | 23.12.28 | Male | 9 | - | - | - | 30.83 | - | - | - | - | - | - | - | - | - | - | - | - | - | - | - | 32.18 |
| 1057 | 23.12.28 | Male | 51 | 27.93 | - | - | 27.92 | - | - | - | - | - | - | - | - | - | - | - | - | - | - | 26.61 | 27.36 |
| 1058 | 23.12.28 | Male | 20 | - | - | - | 29.45 | - | - | - | - | - | - | - | - | - | - | - | - | - | - | - | 30.7 |
| 1059 | 23.12.28 | Male | 48 | - | - | 30.45 | 28.95 | - | - | - | - | - | - | - | - | - | - | - | - | - | 33.08 | - | 29.56 |
| 1060 | 23.12.28 | Female | 22 | - | - | - | 30.23 | - | - | - | - | - | - | - | - | - | - | 29.48 | - | - | - | - | 32.35 |
| 1061 | 23.12.29 | Female | 41 | - | - | 30.89 | 29.66 | - | - | - | - | - | - | - | - | - | - | - | - | - | 33.64 | - | 29.74 |
| 1062 | 23.12.29 | Male | 51 | - | - | - | 30.36 | - | - | - | - | - | - | - | - | - | - | - | - | - | - | - | 32.58 |
| 1063 | 23.12.29 | Female | 37 | - | - | - | 26.96 | - | - | - | - | - | - | - | - | - | - | - | - | - | - | - | 28.07 |
| 1064 | 23.12.29 | Female | 26 | - | - | 30.27 | 30.62 | - | - | - | - | - | - | - | - | - | - | - | - | - | 32.82 | - | 32.26 |
| 1065 | 23.12.29 | Female | 63 | - | - | - | 30.03 | - | - | - | - | - | - | - | - | - | - | - | - | - | - | - | 31.41 |
| 1066 | 23.12.29 | Female | 42 | - | - | - | 30.76 | - | - | - | - | - | - | - | - | - | - | - | - | - | - | - | 32.03 |
| 1067 | 23.12.29 | Female | 12 | - | - | - | 30.23 | - | - | 25.12 | - | 25.07 | - | - | - | - | - | - | - | - | - | - | 31.97 |
| 1068 | 23.12.29 | Male | 40 | - | - | - | 27.97 | - | - | - | - | - | - | - | - | - | - | - | - | - | - | - | 28.62 |
| 1069 | 23.12.29 | Female | 15 | - | - | 28.54 | 29.01 | - | - | - | - | - | - | - | - | - | - | - | - | - | 31.59 | - | 30.31 |
| 1070 | 23.12.29 | Female | 41 | - | - | - | 29.23 | - | - | - | - | - | - | - | - | - | - | - | - | - | - | - | 30.87 |
| 1071 | 23.12.29 | Female | 11 | - | - | - | 30.23 | - | - | - | - | - | - | - | - | - | - | - | - | - | - | - | 31.73 |
| 1072 | 23.12.29 | Male | 53 | - | - | - | 29.7 | - | - | - | - | - | - | - | - | - | - | - | - | - | - | - | 29.99 |
| 1073 | 23.12.29 | Male | 21 | - | - | - | 28.22 | - | - | - | - | - | - | - | - | - | - | - | - | - | - | - | 28.72 |
| 1074 | 24.01.02 | Male | 17 | - | - | - | 30.81 | - | - | - | - | - | - | - | - | - | - | - | - | - | - | - | 32.09 |
| 1075 | 24.01.02 | Male | 38 | - | - | - | 28.73 | - | - | - | - | - | - | 28.83 | - | - | - | - | - | - | - | - | 30.16 |
| 1076 | 24.01.02 | Male | 19 | 29.21 | - | - | 29.33 | - | - | - | - | - | - | - | - | - | - | - | - | - | - | 34.8 | 30.57 |
| 1077 | 24.01.02 | Female | 38 | 34.85 | - | - | 30.32 | - | - | - | - | - | - | - | - | - | - | - | - | - | - | 37.29 | 29.48 |
| 1078 | 24.01.02 | Male | 40 | - | - | 27.06 | 28.03 | - | - | - | - | - | - | - | - | - | - | - | - | - | 28.08 | - | 29.03 |
| 1079 | 24.01.02 | Female | 38 | - | - | 27.51 | 26.32 | - | - | - | - | - | - | - | - | - | - | - | - | - | 30.55 | - | 27.37 |
| 1080 | 24.01.02 | Male | 24 | 28.98 | - | - | 30.47 | - | - | - | - | - | - | - | - | - | - | - | - | - | - | 30.61 | 32.66 |
| 1081 | 24.01.02 | Male | 50 | - | - | - | 26.76 | - | - | - | - | - | - | - | - | - | - | - | - | - | - | - | 26.31 |
| 1082 | 24.01.03 | Female | 50 | - | - | - | 28.16 | - | - | - | - | - | - | - | - | - | - | - | - | - | - | - | 29.18 |
| 1083 | 24.01.03 | Male | 20 | - | - | - | 29.47 | - | - | - | - | - | - | - | - | - | - | - | - | - | - | - | 31.21 |
| 1084 | 24.01.03 | Male | 26 | - | - | 28.99 | 29.49 | - | - | - | - | - | - | - | - | - | - | - | - | - | 29.5 | - | 29.81 |
| 1085 | 24.01.03 | Female | 45 | - | - | - | 30.2 | - | - | - | - | - | - | - | - | - | - | - | - | - | - | - | 30.91 |
| 1086 | 24.01.03 | Female | 46 | - | - | - | 29.31 | - | - | - | - | - | - | - | - | - | - | - | - | - | - | - | 29.86 |
| 1087 | 24.01.03 | Male | 53 | - | - | - | 30.43 | - | - | - | - | 30.01 | - | - | - | - | - | - | - | - | - | - | 32.73 |
| 1088 | 24.01.03 | Male | 31 | 31.83 | - | - | 30.94 | - | - | - | - | - | - | - | - | - | - | - | - | - | - | 33.03 | 32.64 |
| 1089 | 24.01.03 | Male | 21 | - | - | 27.79 | 28.92 | - | - | - | - | - | - | - | - | - | - | - | - | - | 26.95 | - | 28.92 |
| 1090 | 24.01.03 | Female | 67 | - | - | - | 28.22 | - | - | - | - | - | - | - | - | - | - | 29.68 | - | - | - | - | 29.12 |
| 1091 | 24.01.03 | Male | 35 | - | - | 26.78 | 24.59 | - | - | - | - | - | - | - | - | - | - | - | - | - | 35.9 | - | 25.29 |
| 1092 | 24.01.03 | Male | 4 | - | - | - | 28.83 | - | - | - | 27.67 | - | - | - | - | - | - | - | - | - | - | - | 30.35 |
| 1093 | 24.01.03 | Male | 21 | - | - | - | 28.92 | - | - | - | - | - | - | - | - | - | - | - | - | - | - | - | 28.69 |
| 1094 | 24.01.03 | Female | 12 | - | - | - | 30.71 | - | - | - | - | - | - | - | - | - | - | - | - | - | - | - | 32.18 |
| 1095 | 24.01.03 | Female | 45 | - | - | - | 26.26 | - | - | - | - | 28.43 | - | - | - | - | - | - | - | - | - | - | 27.2 |
| 1096 | 24.01.03 | Female | 27 | - | - | 31.42 | 30.97 | - | - | - | - | - | - | - | - | - | - | - | - | - | 34.01 | - | 32.35 |
| 1097 | 24.01.04 | Male | 9 | - | - | 30.49 | 30.25 | - | - | - | - | - | - | - | - | - | - | - | - | - | 38.06 | - | 31.36 |
| 1098 | 24.01.04 | Male | 23 | - | - | 29.82 | 29.59 | - | - | - | - | - | - | - | - | - | - | - | - | - | 36.15 | - | 30.26 |
| 1099 | 24.01.04 | Male | 24 | 30.63 | - | - | 27.22 | - | - | - | - | - | - | - | - | - | - | - | - | - | - | 38.54 | 28.35 |
| 1100 | 24.01.04 | Female | 20 | - | - | - | 29.99 | - | - | - | - | - | - | - | - | - | - | - | - | - | - | - | 30.9 |
| 1101 | 24.01.04 | Female | 35 | - | - | - | 30.04 | - | - | - | - | - | - | - | - | - | - | - | - | - | - | - | 31.74 |
| 1102 | 24.01.04 | Female | 16 | - | - | - | 32.07 | - | - | - | - | - | - | - | - | - | - | - | - | - | - | - | 32.39 |
| 1103 | 24.01.04 | Male | 36 | - | - | - | 30.06 | - | - | - | - | - | - | - | - | - | - | - | - | - | - | - | 31.18 |
| 1104 | 24.01.04 | Male | 20 | - | - | - | 30.97 | - | 27.26 | - | - | - | - | - | - | - | - | - | - | - | - | - | 31.84 |
| 1105 | 24.01.04 | Female | 39 | - | - | - | 30.91 | - | - | - | - | - | - | - | - | - | - | - | - | - | - | - | 32.19 |
| 1106 | 24.01.08 | Female | 52 | - | - | - | 26.55 | - | - | - | - | - | - | - | - | - | - | - | - | - | - | - | 26.48 |
| 1107 | 24.01.08 | Female | 7 | - | - | - | 29.67 | - | - | - | - | - | - | - | - | - | - | - | - | - | - | - | 30.11 |
| 1108 | 24.01.08 | Female | 29 | - | - | - | 26.06 | - | - | - | - | - | - | - | - | - | - | - | - | - | - | - | 30.32 |
| 1109 | 24.01.08 | Female | 61 | 30.33 | - | - | 29.56 | - | - | - | - | - | - | - | - | - | - | - | - | - | - | 35.26 | 33.05 |
| 1110 | 24.01.08 | Female | 50 | - | - | - | 30.54 | - | - | - | - | - | - | - | - | - | - | - | - | - | - | - | 31.23 |
| 1111 | 24.01.08 | Female | 18 | - | - | - | 31.33 | - | - | - | - | 29.11 | - | - | - | - | - | - | - | - | - | - | 30.98 |
| 1112 | 24.01.08 | Female | 74 | - | - | - | 27.93 | - | - | - | - | 26.73 | - | - | - | - | - | - | - | - | - | - | 28.99 |
| 1113 | 24.01.08 | Female | 44 | - | - | - | 26.67 | - | - | - | - | - | - | - | - | - | - | - | - | - | - | - | 27.71 |
| 1114 | 24.01.08 | Male | 25 | 28.61 | - | - | 28.99 | - | - | - | - | - | - | - | - | - | - | - | - | - | - | 29.68 | 30.38 |
| 1115 | 24.01.08 | Male | 33 | - | - | - | 29.97 | - | - | - | - | - | - | - | - | - | - | - | - | - | - | - | 29.58 |
| 1116 | 24.01.08 | Male | 29 | - | - | - | 30.14 | - | - | - | - | - | - | - | - | - | - | - | - | - | - | - | 29.98 |
| 1117 | 24.01.08 | Male | 43 | - | - | - | 26.33 | - | - | - | - | - | - | - | - | - | - | - | - | - | - | - | 27.58 |
| 1118 | 24.01.08 | Male | 20 | - | - | - | 28.95 | - | - | - | - | - | - | - | - | - | - | - | - | - | - | - | 29.32 |
| 1119 | 24.01.09 | Male | 14 | - | - | - | 29.49 | - | - | - | - | - | - | - | - | - | - | - | - | - | - | - | 30.45 |
| 1120 | 24.01.09 | Female | 50 | - | - | - | 28.62 | - | - | - | - | - | - | - | - | - | - | - | - | - | - | - | 28.85 |
| 1121 | 24.01.09 | Female | 47 | - | - | 27.03 | 28.65 | - | - | - | - | - | - | - | - | - | - | - | - | - | 30.2 | - | 31.68 |
| 1122 | 24.01.09 | Male | 35 | - | - | 28.23 | 27.57 | - | - | - | - | - | - | - | - | - | - | - | - | - | 30.73 | - | 27.73 |
| 1123 | 24.01.09 | Female | 47 | - | - | - | 27.85 | - | - | - | - | - | - | - | - | - | - | - | - | - | - | - | 27.55 |
| 1124 | 24.01.09 | Male | 56 | 24.77 | - | - | 25.27 | - | - | - | - | - | - | - | - | - | - | - | - | - | - | 24.82 | 26.43 |
| 1125 | 24.01.09 | Female | 61 | - | - | - | 29.59 | - | - | - | - | 27.16 | - | - | - | - | - | - | - | - | - | - | 31.56 |
| 1126 | 24.01.09 | Female | 29 | - | - | - | 29.42 | - | - | - | - | 27 | - | - | - | - | - | - | - | - | - | - | 30.04 |
| 1127 | 24.01.09 | Female | 48 | 29.75 | - | - | 27.98 | - | 27.06 | - | - | - | - | - | - | - | - | - | - | - | - | 29.97 | 27.53 |
| 1128 | 24.01.09 | Male | 35 | - | - | - | 31.43 | - | - | - | - | - | - | - | - | - | - | - | - | - | - | - | 33.98 |
| 1129 | 24.01.09 | Male | 54 | - | - | - | 29.05 | - | - | - | - | 29.79 | - | - | - | - | - | - | - | - | - | - | 31.01 |
| 1130 | 24.01.09 | Male | 28 | 30.07 | - | - | 30.14 | - | - | - | - | - | - | - | - | - | - | - | - | - | - | 36.7 | 30.72 |
| 1131 | 24.01.09 | Male | 27 | 28.43 | - | - | 29.8 | - | - | - | - | - | - | - | - | - | - | - | - | - | - | 28.74 | 31.02 |
| 1132 | 24.01.09 | Male | 25 | 28 | - | - | 28.62 | - | - | - | - | - | - | - | - | - | - | - | - | - | - | 31.18 | 30.06 |
| 1133 | 24.01.10 | Male | 39 | - | - | - | 30.03 | - | - | - | - | - | - | - | - | - | - | - | - | - | - | - | 33.51 |
| 1134 | 24.01.10 | Female | 46 | - | - | 28.76 | 28.06 | - | - | - | - | - | - | - | - | - | - | - | - | - | 33.18 | - | 28.2 |
| 1135 | 24.01.10 | Female | 32 | - | - | - | 30.91 | - | - | - | - | - | - | - | - | - | - | - | - | - | - | - | 32.01 |
| 1136 | 24.01.10 | Female | 35 | - | - | - | 27.95 | - | - | - | - | - | - | - | - | - | - | - | - | - | - | - | 29.2 |
| 1137 | 24.01.10 | Female | 66 | - | - | - | 25.48 | - | - | - | - | - | - | - | - | - | - | - | - | - | - | - | 26.59 |
| 1138 | 24.01.10 | Male | 38 | - | - | - | 29.64 | - | - | - | - | - | - | - | - | - | - | - | 29.53 | - | - | - | 30.65 |
| 1139 | 24.01.10 | Female | 3 | - | - | - | 29.95 | - | - | - | - | - | - | - | - | - | - | - | - | - | - | - | 31.12 |
| 1140 | 24.01.11 | Female | 37 | - | - | - | 27.85 | - | - | - | - | - | - | - | - | - | - | - | - | - | - | - | 31.11 |
| 1141 | 24.01.11 | Female | 33 | - | - | - | 31.19 | - | - | - | - | - | - | 30.09 | - | - | - | - | - | - | - | - | 32.47 |
| 1142 | 24.01.11 | Female | 41 | - | - | - | 27.8 | - | - | - | - | 26.92 | - | - | - | - | - | - | - | - | - | - | 27.43 |
| 1143 | 24.01.11 | Male | 78 | - | - | - | 26.24 | - | - | - | - | - | - | - | - | - | - | - | - | - | - | - | 27.62 |
| 1144 | 24.01.11 | Female | 52 | 33.05 | - | - | 28.55 | - | - | - | - | - | - | - | - | - | - | - | - | - | - | 31.59 | 29.82 |
| 1145 | 24.01.11 | Female | 52 | - | - | - | 27.79 | - | - | - | - | - | - | - | - | - | - | - | - | - | - | - | 29.83 |
| 1146 | 24.01.11 | Female | 19 | - | - | - | 30.1 | - | - | - | - | - | - | - | - | - | - | - | - | - | - | - | 33.38 |
| 1147 | 24.01.11 | Male | 53 | 28.86 | - | - | 27.63 | - | - | - | - | - | - | - | - | - | - | - | - | - | - | 32.93 | 29.64 |
| 1148 | 24.01.11 | Male | 19 | - | - | - | 29.82 | - | - | - | - | - | - | - | - | - | - | - | - | - | - | - | 30.93 |
| 1149 | 24.01.11 | Female | 22 | - | - | - | 30.16 | - | - | - | - | - | - | - | - | - | - | - | - | - | - | - | 32.54 |
| 1150 | 24.01.11 | Male | 19 | - | - | - | 30.54 | - | - | - | - | - | - | - | - | - | - | - | - | - | - | - | 33.47 |
| 1151 | 24.01.11 | Male | 19 | - | - | - | 32.28 | - | - | - | - | - | - | - | - | - | - | - | - | - | - | - | 34.68 |
| 1152 | 24.01.11 | Female | 60 | - | - | - | 32.27 | - | - | - | - | - | - | - | - | - | - | - | - | - | - | - | 34.32 |
| 1153 | 24.01.11 | Male | 17 | - | - | 29.18 | 30.73 | - | - | - | - | - | - | - | - | - | - | - | - | - | 29.12 | - | 32.15 |
| 1154 | 24.01.11 | Female | 55 | - | - | - | 29.23 | - | - | - | - | - | - | - | - | - | - | - | - | - | - | - | 32.13 |
| 1155 | 24.01.12 | Male | 38 | 26.55 | - | - | 29.9 | - | - | - | - | - | - | - | - | - | - | - | - | - | - | 26.48 | 31.62 |
| 1156 | 24.01.12 | Male | 57 | - | - | - | 30.02 | - | - | - | - | - | - | - | - | - | - | - | - | - | - | - | 30.61 |
| 1157 | 24.01.12 | Male | 15 | - | - | - | 32.26 | - | - | - | - | - | - | - | - | - | - | - | - | - | - | - | 34.11 |
| 1158 | 24.01.12 | Female | 4 | - | - | - | 29.69 | - | - | - | - | - | - | - | - | - | - | - | - | - | - | - | 30.59 |
| 1159 | 24.01.12 | Female | 54 | - | - | - | 29.83 | - | - | - | - | - | - | - | - | - | - | - | - | - | - | - | 31.16 |
| 1160 | 24.01.12 | Male | 22 | - | - | - | 29.81 | - | - | - | - | - | - | - | - | - | - | - | - | - | - | - | 31.1 |
| 1161 | 24.01.12 | Female | 35 | - | - | - | 30.06 | - | - | - | - | - | - | - | - | - | - | - | - | - | - | - | 31.5 |
| 1162 | 24.01.12 | Male | 11 | - | - | 25.97 | 30.41 | - | - | - | - | - | - | - | - | - | - | - | - | - | 25.84 | - | 34.09 |
| 1163 | 24.01.12 | Female | 12 | - | - | 29.39 | 29.45 | - | - | - | - | - | - | - | - | - | - | - | - | - | 32.39 | - | 30.95 |
| 1164 | 24.01.12 | Female | 11 | - | - | - | 30.53 | - | - | - | - | - | - | - | - | - | - | - | - | - | - | - | 32.8 |
| 1165 | 24.01.12 | Male | 34 | - | - | - | 29.94 | - | - | - | - | - | - | - | - | - | - | - | - | - | - | - | 31.56 |
| 1166 | 24.01.12 | Male | 9 | - | - | 25.87 | 28.75 | - | - | - | - | - | - | - | - | - | - | - | - | - | 27.13 | - | 30.57 |
| 1167 | 24.01.12 | Female | 21 | - | - | - | 29.9 | - | - | - | - | - | - | - | - | - | - | - | - | - | - | - | 30.65 |
| 1168 | 24.01.12 | Male | 11 | - | 29.84 | - | 29.54 | - | - | - | - | - | - | - | - | - | - | - | - | - | - | - | 30.86 |
| 1169 | 24.01.12 | Female | 24 | 28.19 | - | - | 31.25 | - | - | - | - | - | - | - | - | - | - | - | - | - | - | 28.45 | 32.07 |
| 1170 | 24.01.12 | Male | 6 | 29.14 | - | - | 28.66 | - | - | - | - | - | - | - | - | - | - | - | - | - | - | 32.24 | 29.41 |
| 1171 | 24.01.15 | Male | 43 | - | - | - | 26.77 | - | - | - | - | - | - | - | - | - | - | - | - | - | - | - | 27.73 |
| 1172 | 24.01.15 | Female | 10 | - | - | - | 27.88 | - | - | - | - | - | - | - | - | - | - | - | - | - | - | - | 28.15 |
| 1173 | 24.01.15 | Male | 11 | - | - | - | 30.72 | - | - | - | - | - | - | - | - | - | - | - | - | - | - | - | 31.6 |
| 1174 | 24.01.16 | Male | 6 | - | - | - | 30.78 | - | - | - | - | - | - | - | - | - | - | - | - | - | - | - | 32.89 |
| 1175 | 24.01.17 | Female | 47 | - | - | - | 31.66 | - | - | - | - | - | - | - | - | - | - | - | - | - | - | - | 33.38 |
| 1176 | 24.01.17 | Female | 8 | - | - | 32.06 | 31.93 | - | - | - | - | - | - | - | - | - | - | - | - | - | 38.23 | - | 31.54 |
| 1177 | 24.01.17 | Male | 25 | - | - | - | 28.99 | - | - | - | - | 28.47 | - | - | - | - | - | - | - | - | - | - | 30.66 |
| 1178 | 24.01.17 | Male | 48 | 28.59 | - | - | 28.51 | - | - | - | - | - | - | - | - | - | - | - | - | - | - | 30.98 | 29.72 |
| 1179 | 24.01.17 | Female | 35 | - | - | - | 30.89 | - | - | - | - | - | - | - | - | 35.22 | - | - | - | - | - | - | 32.17 |
| 1180 | 24.01.17 | Male | 9 | - | - | - | 27.77 | - | - | - | - | - | - | - | - | - | - | - | - | - | - | - | 27.57 |
| 1181 | 24.01.17 | Female | 8 | - | - | - | 29.12 | - | - | - | - | - | - | - | - | - | - | - | - | - | - | - | 31.19 |
| 1182 | 24.01.18 | Female | 4 | - | - | - | 31.34 | - | - | - | - | - | - | - | - | - | - | - | - | - | - | - | 33.54 |
| 1183 | 24.01.18 | Male | 29 | - | - | - | 29.03 | - | - | - | - | - | - | - | - | - | - | - | - | - | - | - | 31.17 |
| 1184 | 24.01.18 | Male | 27 | - | - | - | 30.52 | - | - | - | - | 28.16 | - | - | - | - | - | - | - | - | - | - | 30.34 |
| 1185 | 24.01.18 | Male | 5 | - | - | 25.87 | 31.23 | - | - | - | - | - | - | - | - | - | - | - | - | - | 23.91 | - | 32.64 |
| 1186 | 24.01.19 | Male | 69 | - | - | - | 26.72 | - | - | - | - | - | - | - | - | - | - | - | - | - | - | - | 26.91 |
| 1187 | 24.01.22 | Female | 37 | - | - | - | 29.01 | - | - | - | - | 28.62 | - | - | - | - | - | - | - | - | - | - | 29.83 |
| 1188 | 24.01.22 | Female | 27 | - | - | - | 30.56 | - | - | - | - | - | - | - | - | - | - | - | - | - | - | - | 31.14 |
| 1189 | 24.01.22 | Female | 34 | - | - | - | 29.38 | - | - | - | - | - | - | - | - | - | - | - | - | - | - | - | 30 |
| 1190 | 24.01.22 | Female | 25 | - | - | - | 27.72 | - | 26.76 | - | - | - | - | - | - | - | - | - | - | - | - | - | 27.63 |
| 1191 | 24.01.22 | Female | 25 | - | - | - | 29.48 | - | - | - | - | - | - | - | - | - | - | - | - | - | - | - | 32.01 |
| 1192 | 24.01.23 | Male | 57 | - | - | - | 29.78 | - | - | - | - | - | 30 | - | - | - | - | - | - | - | - | - | 30.66 |
| 1193 | 24.01.23 | Female | 60 | - | - | - | 30.17 | - | - | - | - | - | - | - | - | - | - | - | - | - | - | - | 33.28 |
| 1194 | 24.01.23 | Female | 41 | - | - | - | 26.57 | - | - | - | - | - | - | - | - | - | - | - | - | - | - | - | 27.41 |
| 1195 | 24.01.23 | Male | 16 | - | - | - | 30.59 | - | - | - | - | 28.76 | - | - | - | - | - | - | - | - | - | - | 33.59 |
| 1196 | 24.01.23 | Female | 49 | - | - | - | 28.08 | - | - | - | - | - | - | - | - | - | - | - | - | - | - | - | 27.93 |
| 1197 | 24.01.23 | Male | 6 | - | - | - | 30 | - | - | 26.68 | - | 26.48 | - | 30.63 | - | - | - | - | - | - | - | - | 29.74 |
| 1198 | 24.01.23 | Female | 35 | - | - | - | 27.59 | - | - | - | - | - | - | - | - | - | - | - | - | - | - | - | 28.32 |
| 1199 | 24.01.23 | Male | 53 | - | - | - | 30.87 | - | - | - | - | - | - | - | - | - | - | - | - | - | - | - | 32.01 |
| 1200 | 24.01.23 | Male | 57 | - | - | - | 30.17 | - | - | - | - | - | - | - | - | - | - | - | - | - | - | - | 30.74 |
| 1201 | 24.01.23 | Female | 55 | - | - | - | 29.21 | - | - | - | 26.87 | - | - | - | - | - | - | - | - | - | - | - | 30.2 |
| 1202 | 24.01.23 | Female | 34 | - | - | - | 28.14 | - | - | - | - | - | - | - | - | - | - | - | - | - | - | - | 27.93 |
| 1203 | 24.01.23 | Male | 44 | - | - | - | 30.21 | - | - | - | - | 29.71 | - | - | - | - | - | - | - | - | - | - | 30.26 |
| 1204 | 24.01.24 | Female | 47 | - | - | - | 27.49 | - | - | - | - | - | - | - | - | - | - | - | - | - | - | - | 28.04 |
| 1205 | 24.01.24 | Female | 29 | - | - | - | 29.5 | - | - | - | - | - | - | - | - | - | - | - | - | - | - | - | 30.58 |
| 1206 | 24.01.24 | Male | 38 | - | - | - | 30.68 | - | - | - | - | - | - | - | - | - | - | - | - | - | - | - | 31.26 |
| 1207 | 24.01.24 | Female | 40 | - | - | - | 29.46 | - | - | - | - | - | - | - | - | - | - | - | - | - | - | - | 29.28 |
| 1208 | 24.01.24 | Male | 47 | - | - | - | 29.43 | - | - | - | - | - | - | - | - | - | - | - | - | - | - | - | 30.96 |
| 1209 | 24.01.24 | Female | 70 | - | - | - | 27.61 | - | - | - | - | - | - | - | - | - | - | - | - | - | - | - | 28.67 |
| 1210 | 24.01.24 | Male | 18 | - | - | - | 32.43 | - | - | - | - | - | - | - | - | - | - | - | - | - | - | - | 35.18 |
| 1211 | 24.01.24 | Female | 74 | - | - | - | 31.04 | - | - | - | - | - | - | - | - | - | - | - | - | - | - | - | 31.56 |
| 1212 | 24.01.24 | Female | 53 | - | - | - | 30.63 | - | - | - | - | - | - | - | - | - | - | - | - | - | - | - | 31.23 |
| 1213 | 24.01.24 | Female | 60 | - | - | - | 26.46 | - | - | - | - | - | - | - | - | - | - | - | - | - | - | - | 27.31 |
| 1214 | 24.01.24 | Female | 27 | - | - | - | 29.21 | - | - | - | - | - | - | - | - | - | - | - | - | - | - | - | 29.64 |
| 1215 | 24.01.25 | Female | 40 | - | - | - | 28.97 | - | - | - | - | - | - | - | - | - | - | - | - | - | - | - | 29.08 |
| 1216 | 24.01.25 | Male | 31 | - | - | - | 30.32 | - | - | - | - | 25.76 | - | - | - | - | - | - | - | - | - | - | 32.36 |
| 1217 | 24.01.25 | Male | 29 | - | 30.51 | - | 27.87 | - | - | - | - | - | - | - | - | - | - | - | - | - | - | - | 27.51 |
| 1218 | 24.01.25 | Female | 63 | - | - | - | 28.55 | - | - | - | - | - | - | - | - | - | - | - | - | - | - | - | 28.67 |
| 1219 | 24.01.25 | Male | 74 | - | - | - | 26.51 | - | - | - | - | - | - | - | - | - | - | - | - | - | - | - | 27.49 |
| 1220 | 24.01.25 | Male | 31 | - | - | - | 29.3 | - | - | - | - | - | - | - | - | - | - | - | - | - | - | - | 30.59 |
| 1221 | 24.01.25 | Female | 24 | - | - | - | 30.59 | - | - | - | - | - | - | - | - | - | - | - | - | - | - | - | 31.28 |
| 1222 | 24.01.25 | Female | 25 | - | - | - | 29.85 | - | - | - | 34.51 | - | - | - | - | - | - | - | - | - | - | - | 30.61 |
| 1223 | 24.01.25 | Male | 65 | - | - | - | 27.8 | - | - | - | - | - | - | - | - | - | - | - | - | - | - | - | 28.55 |
| 1224 | 24.01.25 | Female | 11 | - | - | - | 27.03 | - | - | - | - | 24.76 | - | - | - | - | - | - | - | - | - | - | 28.68 |
| 1225 | 24.01.25 | Male | 55 | - | - | - | 25.25 | - | - | - | - | - | - | - | - | - | - | - | - | - | - | - | 27.11 |
| 1226 | 24.01.25 | Female | 27 | - | - | - | 30.5 | - | - | - | - | - | - | - | - | - | - | - | - | - | - | - | 32.08 |
| 1227 | 24.01.25 | Male | 5 | - | - | - | 30.63 | - | - | - | - | - | - | - | - | - | - | - | - | - | - | - | 32.45 |

Abbreviations: No., number; qRT-PCR, quantitative real-time polymerase chain reaction; Ct, cycle threshold; IBV, influenza B virus; ADV, adenovirus; IAV, influenza A virus; IC, internal control; SARS-CoV-2, severe acute respiratory syndrome coronavirus 2; HSV-1, herpes simplex virus type 1; EV, enterovirus; HMPV, human metapneumovirus; HRV, human rhinovirus; BP, *Bordetella pertussis*; MP, *Mycoplasma pneumoniae*; CP, *Chlamydia pneumoniae*; RSV, respiratory syncytial virus; HBoV, human bocavirus; HPIV, human parainfluenza virus; HCoV, human coronavirus; -, negative.

**Table S8** Comparison of the FQ-8B with Other Rapid Diagnostic Platforms for Respiratory Pathogens

| **Platform** | **Time required** | **Detection throughput** | **Number of targets** | **NA extraction included** | **Reagent openness** | **Amplification characteristics** |
| --- | --- | --- | --- | --- | --- | --- |
| **FQ-8B** (30-min multi-pathogen detection) | 28 min | 2 | 15 | No | Yes | Three ITMs cycling and a time-based algorithm |
| **FilmArray** (respiratory pathogen panel) | 1 h | 1 | 21 (up to 31) | Yes | No | Nested PCR and melting curve analysis |
| **GeneXpert** (SARS-CoV-2, IAV, IBV, and RSV) | 45 min | 1 (depending on instrument models) | 4 | Yes | No | Cartridge; single module independent reaction |
| **GENECUBE** (SARS-CoV-2, IAV, and IBV) | 25 min | 8–24 | 4 | No | No | Dedicated capillary tubes for PCR, QProbe |
| **MONITOR** (microfluidic chip) | 1.5 h | 1 | 8 (up to 32) | Yes | No | NA evenly partitioned into several chambers for PCR |
| **Panther Fusion** (respiratory assays, a complete set of three assays for respiratory viruses) | 2.4 h | 32 | 10 | Yes | No | PCR and TMA, with continuous and random access |

Abbreviations: NA, nucleic acid; ITMs, independent temperature modules.
