# Supplementary figures and images for: Development and clinical application of a rapid qPCR instrument featuring three independent temperature modules and a time-based algorithm for respiratory pathogen diagnosis
Source: Virol J. 2025 Nov 21;22:381. doi: 10.1186/s12985-025-03003-2 (PMC12639651; doi:10.1186/s12985-025-03003-2)

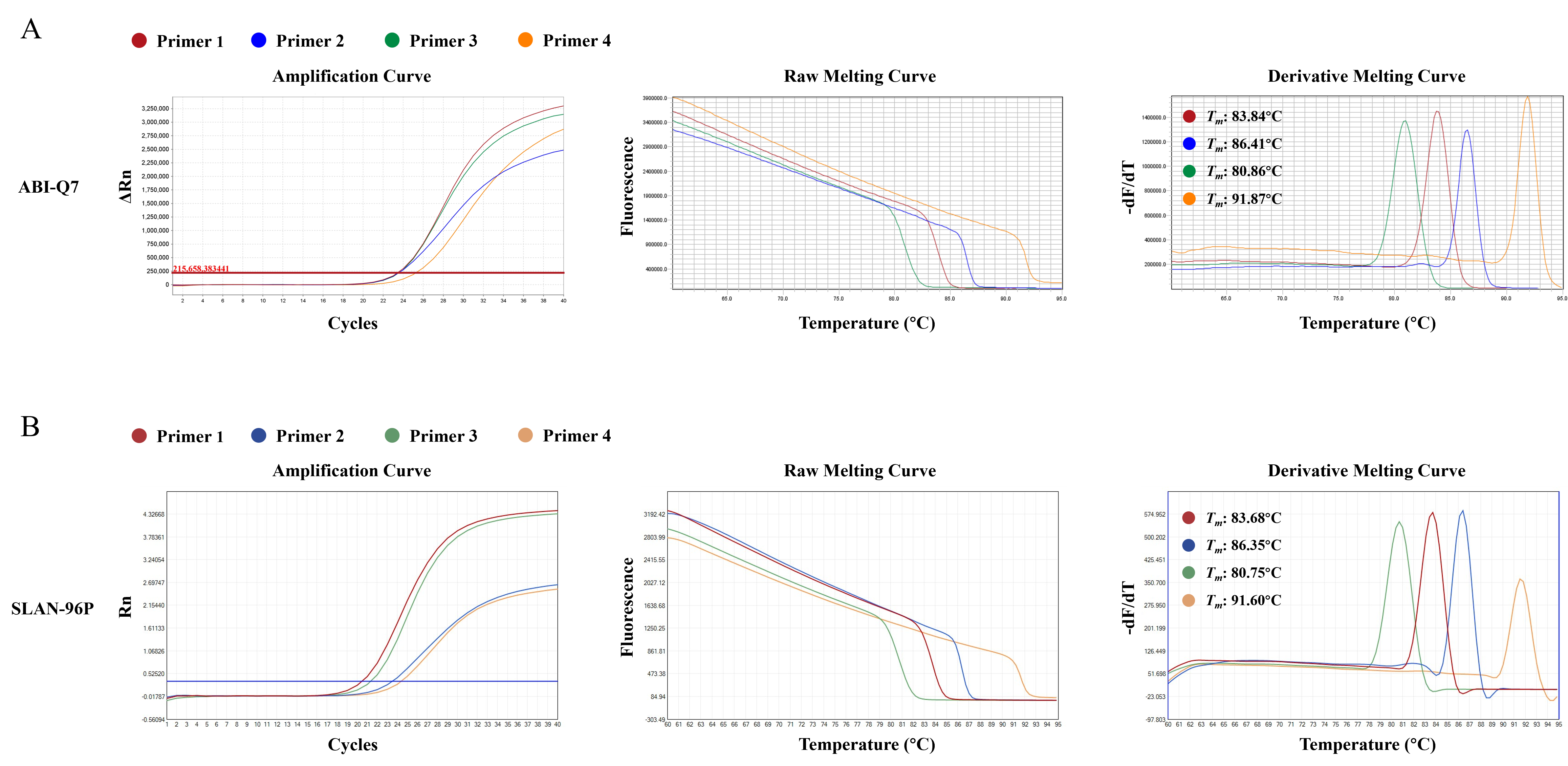

Supplement: Supplementary file 2 — Supplementary Material 2 [file 12985_2025_3003_MOESM2_ESM.tif]

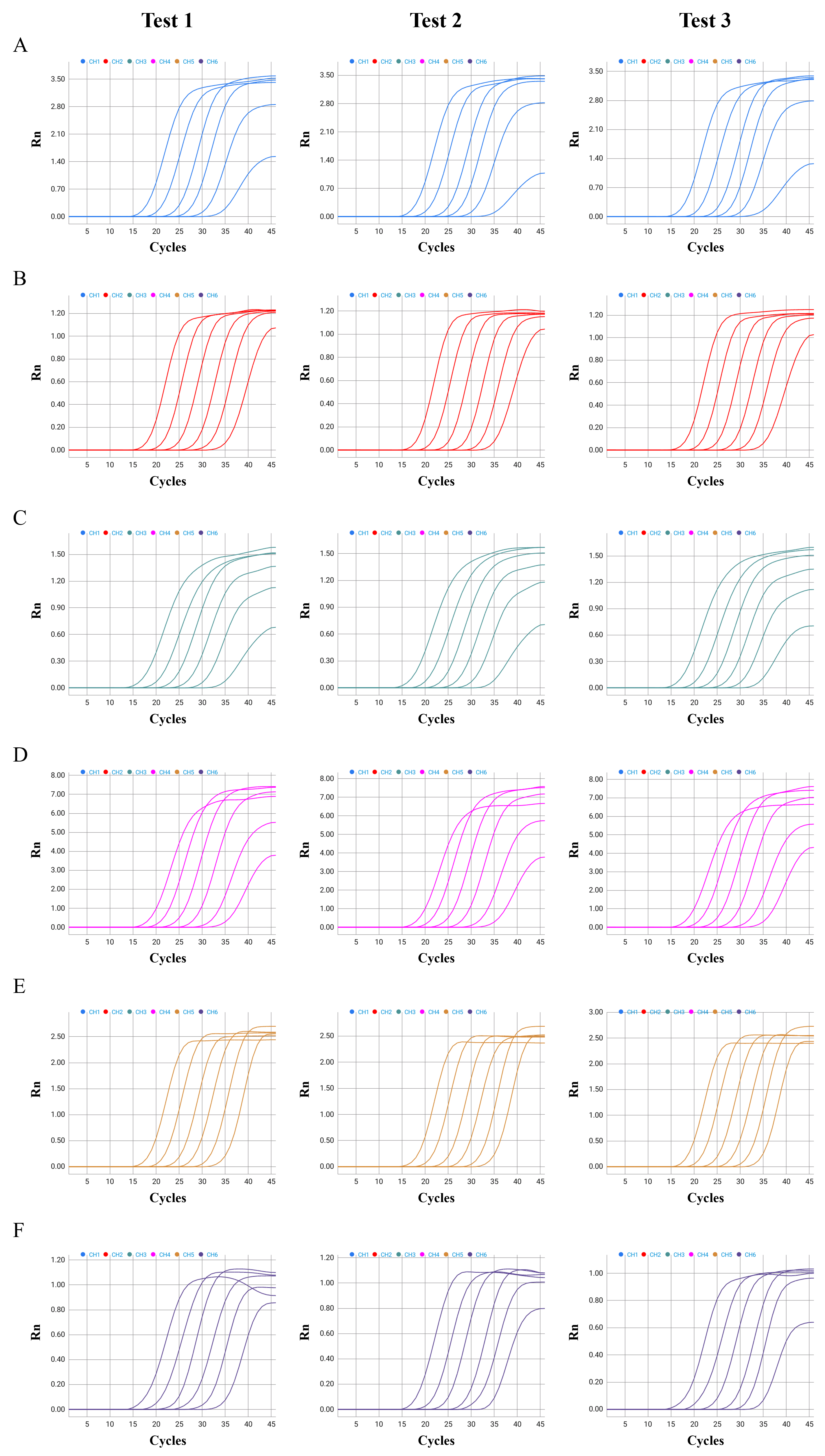

Supplement: Supplementary file 3 — Supplementary Material 3 [file 12985_2025_3003_MOESM3_ESM.tif]

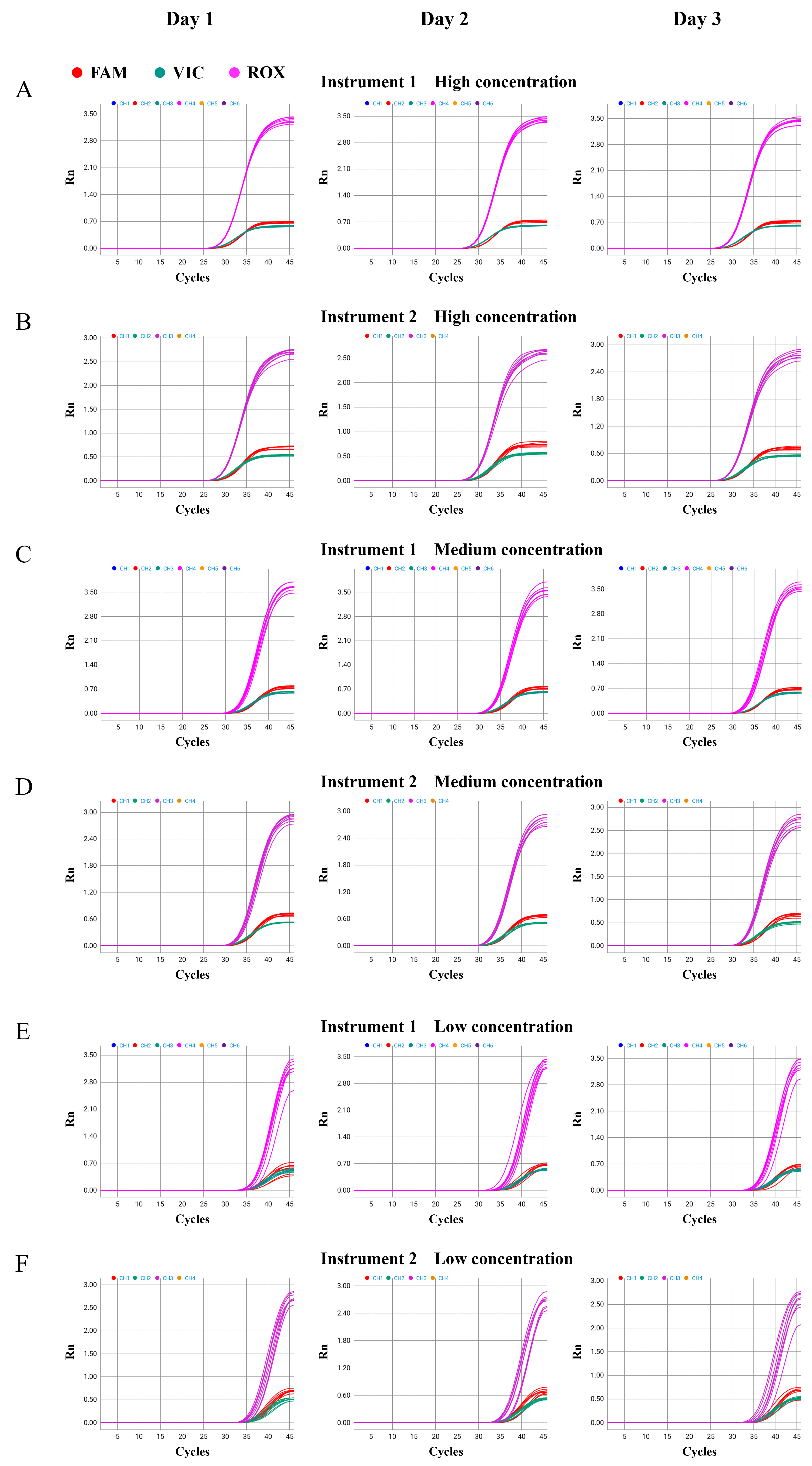

Supplement: Supplementary file 4 — Supplementary Material 4 [file 12985_2025_3003_MOESM4_ESM.tif]

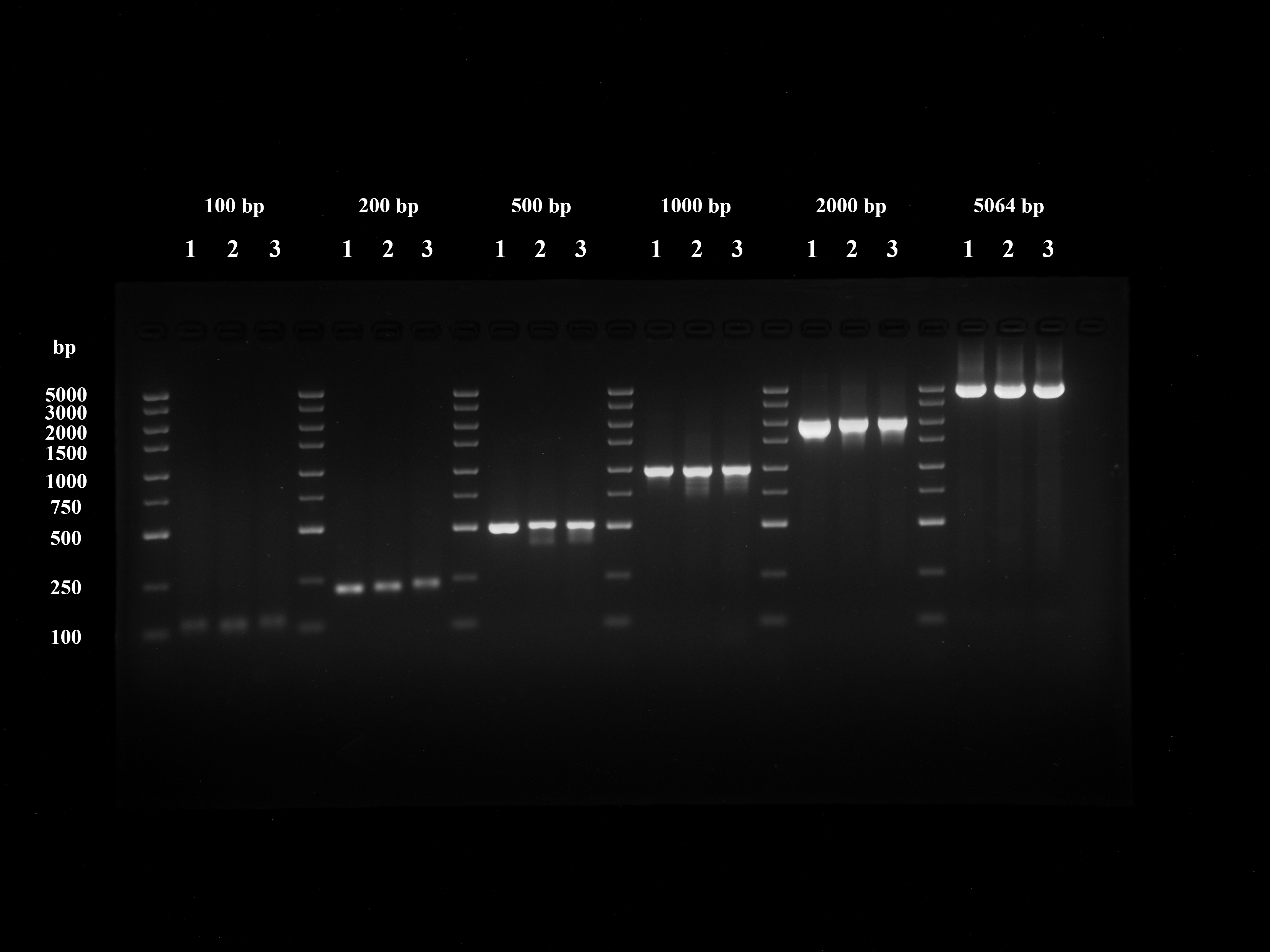

Supplement: Supplementary file 5 — Supplementary Material 5 [file 12985_2025_3003_MOESM5_ESM.tif]
